# A Genetic History of the Near East from an aDNA Time Course Sampling Eight Points in the Past 4,000 Years

**DOI:** 10.1016/j.ajhg.2020.05.008

**Published:** 2020-05-28

**Authors:** Marc Haber, Joyce Nassar, Mohamed A. Almarri, Tina Saupe, Lehti Saag, Samuel J. Griffith, Claude Doumet-Serhal, Julien Chanteau, Muntaha Saghieh-Beydoun, Yali Xue, Christiana L. Scheib, Chris Tyler-Smith

**Affiliations:** 1 Institute of Cancer and Genomic Sciences, University of Birmingham, Birmingham B15 2TT, UK; 2 Centre for Computational Biology, University of Birmingham, Birmingham B15 2TT, UK; 3 Wellcome Sanger Institute, Wellcome Genome Campus, Hinxton CB10 1SA, UK; 4 Institut Français du Proche-Orient, BP 11-1424, Beirut, Lebanon; 5 Institute of Genomics, University of Tartu, Riia 23b, 51010 Tartu, Estonia; 6 Department of Evolutionary Biology, Institute of Cell and Molecular Biology, University of Tartu, Tartu 51010, Estonia; 7 The Sidon Excavation, Saida, Lebanon; 8 Département des Antiquités Orientales, Musée du Louvre, France; 9 Université Libanaise, Rectorat, BP 14-6573, Place du Musée, Beirut, Lebanon

**Keywords:** Lebanon, Beirut, Bronze Age, Iron Age, Classical Antiquity, whole-genome sequences, population genetics, migration, admixture, culture

## Abstract

The Iron and Classical Ages in the Near East were marked by population expansions carrying cultural transformations that shaped human history, but the genetic impact of these events on the people who lived through them is little-known. Here, we sequenced the whole genomes of 19 individuals who each lived during one of four time periods between 800 BCE and 200 CE in Beirut on the Eastern Mediterranean coast at the center of the ancient world’s great civilizations. We combined these data with published data to traverse eight archaeological periods and observed any genetic changes as they arose. During the Iron Age (∼1000 BCE), people with Anatolian and South-East European ancestry admixed with people in the Near East. The region was then conquered by the Persians (539 BCE), who facilitated movement exemplified in Beirut by an ancient family with Egyptian-Lebanese admixed members. But the genetic impact at a population level does not appear until the time of Alexander the Great (beginning 330 BCE), when a fusion of Asian and Near Easterner ancestry can be seen, paralleling the cultural fusion that appears in the archaeological records from this period. The Romans then conquered the region (31 BCE) but had little genetic impact over their 600 years of rule. Finally, during the Ottoman rule (beginning 1516 CE), Caucasus-related ancestry penetrated the Near East. Thus, in the past 4,000 years, three limited admixture events detectably impacted the population, complementing the historical records of this culturally complex region dominated by the elite with genetic insights from the general population.

## Main Text

The ancient Near East has been at the center of interaction between the ancient world’s civilizations and was ruled at different times by the Egyptians, Hittites, Assyrians, Babylonians, Persians, Greeks, Romans, Arabs, Crusaders, Mamluks, and Ottomans, most of whom left a permanent cultural impact on the local population. However, their genetic contribution is not as evident: our previous ancient DNA (aDNA) work showed that people who live in the Near East today derive ∼90% of their ancestry from the local Bronze Age population that preceded all of the aforementioned historical conquests.^1^ These results might appear to challenge the historical records of population movements, colonization, and admixture with the locals throughout history. For example, in 1307 CE, the Mamluks divided Lebanon’s coast among 300 newly introduced Turkoman families, and a few centuries earlier the Romans had declared Beirut and Baalbek in Lebanon as colonies and garrison towns;^2^ additionally the names of Hellenistic army soldiers and their descendants in Lebanon can still be read today from inscriptions on funerary stela found in Sidon.^3^ Similarly, our analysis of aDNA from a Crusader burial site in Lebanon showed that immigration to the Near East and admixture with the locals was common, and for a period, a heterogeneous population of Europeans, locals, and their admixed descendants lived in the Near East.^4^ However, this admixture appears to not have been widespread enough to leave a permanent genetic impact on the local population, and subsequent mixing with people carrying the local ancestry “diluted” the ancestry of the Crusaders in Near Eastern genomes to undetectable levels. The example of the Crusaders might illustrate why, even after numerous conquests and immigrations, the Near Eastern Bronze Age ancestry still dominates present-day Near Eastern genomes. Thus, two outstanding questions emerge from the previous aDNA studies: (1) were transient admixture events a common occurrence in the history of the Near East, or was the Crusaders period an exception, and (2) because present-day Near Easterners derive most but not all of their ancestry from the local Bronze Age population, which post-Bronze Age events contributed to the genetic diversity we observe today in the Near East.

To address these questions, we have now sequenced the genomes of ancient individuals who lived between 800 BCE and 200 CE at one of four different time periods: the Iron Age II (1000–539 BCE), the Iron Age III (539–330 BCE), the Hellenistic period (330–31 BCE), and the early Roman period (31 BCE–200 CE) (Table 1). These data, together with previous data we generated from individuals from the same region from the Middle Bronze Age (2100–1550 BCE), the late Roman period (200–634 CE), the Crusader period (1099–1291 CE), and the present-day provide a genetic representation of the Near East in a time series spanning the past 4,000 years (Table S1).

**Table 1:** Samples Analyzed in this Study

**ENA Number**	**ID**	**Excavation Site**	**Period**	**Date (Calibrated)**	**Mapped Read %**	**Coverage Genomic**	**SNPs Overlapping with Published aDNA Data**
ERS4542976	SFI-56	Beirut SFI-415	Iron Age II	–	15	0.7	568,628
ERS4542991	SFI-55	Beirut SFI-415	Iron Age II	–	8	0.4	408,015
ERS4542962	SFI-43	Beirut SFI-1075	Iron Age III	567 BCE–404 BCE	17	0.5	428,888
ERS4542967	SFI-50	Beirut SFI-1075	Iron Age III	–	31	1	703,041
ERS4542969	SFI-36	Beirut SFI-1075	Iron Age III	–	19	0.8	590,514
ERS4542989	SFI-42	Beirut SFI-1075	Iron Age III	540 BCE–396 BCE	13	0.5	440,585
ERS4542964	SFI-45	Beirut SFI-1075	Iron Age III	–	24	0.6	478,277
ERS4542984	SFI-34	Beirut SFI-1075	Iron Age III	–	27	1.7	933,032
ERS4542983	SFI-35	Beirut SFI-1075	Iron Age III	–	5	0.3	321,527
ERS4542988	SFI-39	Beirut SFI-1075	Iron Age III	–	13	0.7	567,178
ERS4542990	SFI-44	Beirut SFI-1075	Iron Age III	–	41	1.6	889,705
ERS4542987	SFI-47	Beirut SFI-1075	Iron Age III	–	23	1.1	747,390
ERS4542979	SFI-20	Beirut SFI-477	Hellenistic	199 BCE–37 BCE	13	0.8	691,379
ERS4542972	SFI-5	Beirut SFI-477	Hellenistic	234 BCE–92 BCE	3	0.1	140,660
ERS4542974	SFI-12	Beirut SFI-477	Hellenistic	209 BCE–89 BCE	2	0.1	106,051
ERS4542980	SFI-24	Beirut SFI-1106	early Roman	55 BCE–58 CE	39	3.3	1,093,459
ERS4542982	SFI-33	Beirut SFI-1106	early Roman	48 CE–222 CE	43	3.3	1,087,690
ERS4542973	SFI-11	Beirut SFI-477	early Roman	119 BCE–27 CE	2	0.1	132,450
ERS4542977	SFI-15	Beirut SFI-477	early Roman	176 BCE–3 CE	28	1.4	915,901

We sampled the petrous portion of the temporal bones from 67 individuals buried in Beirut (Figures S1 and S2), a city on the Eastern Mediterranean coast that has had continuous settlement dating back 5,000 years and that is the capital of modern-day Lebanon. We extracted DNA and built double-stranded libraries according to published protocols^5^, ^6^, ^7^ and sequenced the libraries on Illumina HiSeq 2500 and HiSeq 4000 platforms with 2 × 75 bp reads. We processed the sequences by using PALEOMIX^8^ as described previously^4^ and mapped the merged sequences to the hs37d5 reference sequence (see Supplemental Methods). We found 19 samples that had 2%–43% endogenous DNA with post-mortem damage patterns typical of ancient DNA (Figure S3), and subsequent sequencing of these libraries resulted in genomic coverage between 0.1× and 3.3× (Table 1). We estimated contamination from the X chromosomes of males and the mtDNA genome of all individuals^9^^,^^10^ and found that the sequence data were minimally contaminated (Tables S2 and S3).

We combined the new data with published ancient and modern data, creating two datasets: set 1 included 2,012 modern humans^1^^,^^11^, ^12^, ^13^, ^14^ and 914 ancient individuals^6^^,^^15^, ^16^, ^17^, ^18^, ^19^, ^20^, ^21^, ^22^, ^23^, ^24^, ^25^, ^26^, ^27^, ^28^, ^29^, ^30^, ^31^, ^32^, ^33^, ^34^, ^35^, ^36^, ^37^ with 815,791 SNPs, and set 2 consisted of 2,788 modern humans^24^^,^^38^^,^^39^ and 914 ancient individuals with 539,766 SNPs (see Supplemental Methods).

**Figure 1:**
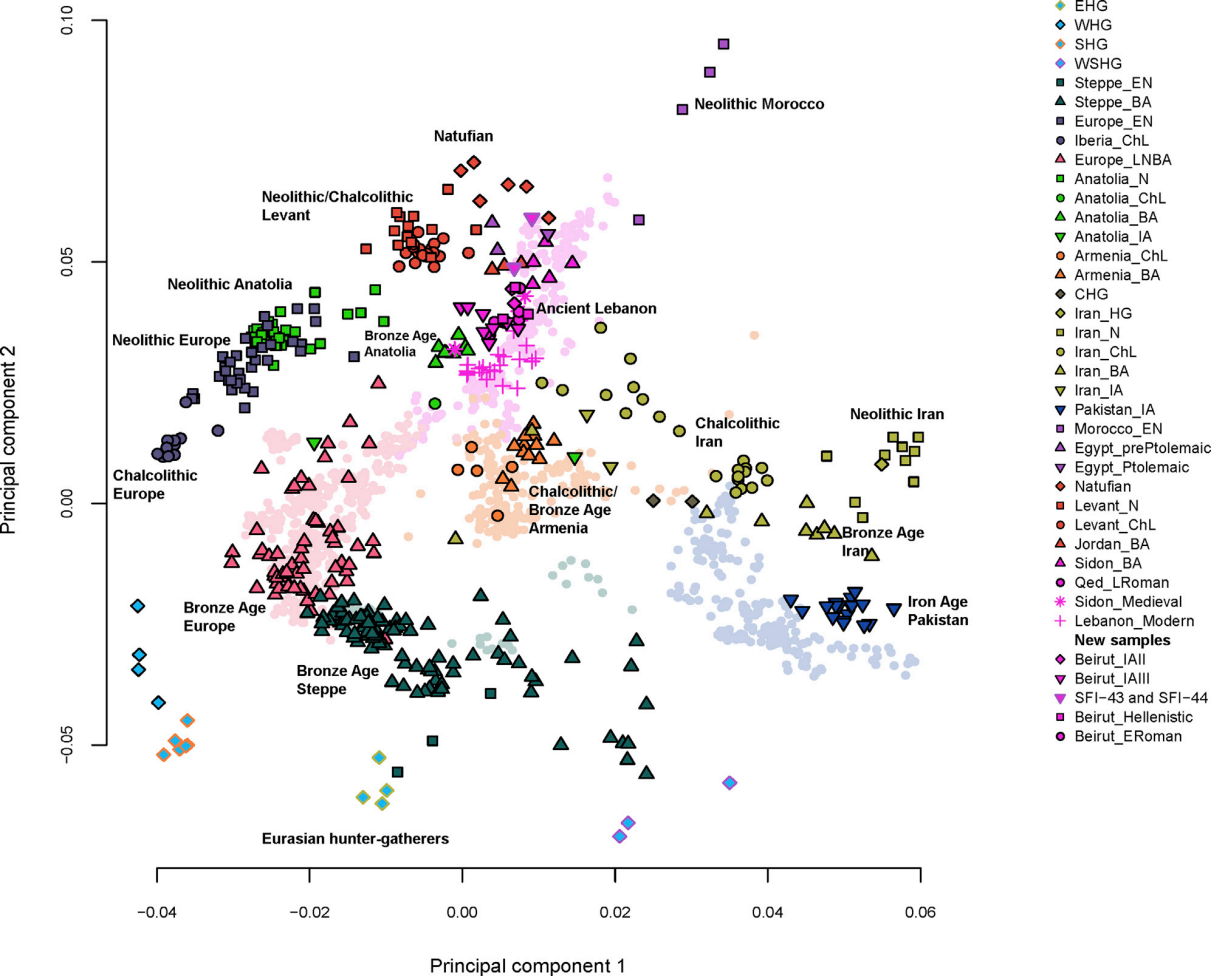
Principal Components Analysis of West, Central, and South Eurasians Eigenvectors were inferred with present-day populations (light-colored points in the background of the plot), and the ancient samples (colored solid shapes in the foreground of the plot) were projected onto the plot.

We then estimated kinship^40^ among our samples and found individuals SFI-43 (female) and SFI-44 (male), who lived around 500 BCE during the Iron Age III under the Persian rule, were first-degree relatives (Figure S4) and shared the same mtDNA haplogroup, T2C1 (Table S4). We kept these two individuals in the dataset for the following test and projected all ancient samples in set 2 onto a principal component analysis (PCA)^41^ plot based on variation in modern West, Central and South Eurasians (Figures 1 and S5). The plot differentiates between populations from the Near East, Europe, Caucasus, Russian Steppe, Central and South Asia. The ancient Lebanese (i.e., ancient individuals who lived in what is today known as Lebanon) clustered with the modern and ancient Near Easterners: the new samples clustered between the Bronze Age population (Sidon_BA) and modern Lebanese. The two first-degree relatives, SFI-43 and SFI-44, appeared as outliers and did not cluster with their contemporaries, but instead were positioned close to the Bronze Age samples. We wanted to test whether these two individuals had a genetic affinity to a population other than the ancient Lebanese. Thus, using *qpWave*,^42^^,^^43^ we selected 11 outgroups (see Supplemental Methods) that have different relationships with the populations found in set 1 and tested whether SFI-43 and SFI-44 formed a clade with any of the populations (including the ancient Lebanese) in our dataset. We found that SFI-43 only formed a clade with ancient Egyptians (Table S5), implying that she shared all of her ancestry with them or a genetically equivalent population. On the other hand, SFI-44’s ancestry appeared to be more complex because he did not form a clade with any population in our dataset, yet he appeared to share ancestry with SFI-43, ancient Egyptians, and ancient Levantines (Table S5). To better understand the relationship of SFI-43 and SFI-44 with the Lebanese and Egyptians, we projected the ancient Lebanese and ancient Egyptians onto a PCA constructed with the variation found in their modern populations. SFI-43 and SFI-44 clustered with the ancient Egyptians and were positioned between modern or ancient Lebanese and modern Egyptians, but SFI-44 was positioned closer than SFI-43 to the Lebanese (Figure S6). Because SFI-43 and SFI-44 are first-degree relatives but appear to have differences in their genetic ancestry, we tested whether SFI-44 can be modeled as a mixture of ancestries deriving from SFI-43 and any other individuals or populations in our dataset by using *qpAdm.*^42^ We found that SFI-43 could be modeled as deriving ∼70% of his ancestry from a population related to SFI-44 and ∼30% from a population related to ancient Levantines (Table S6). But these ancestry proportions do not reflect the first-degree relationship that the two individuals shared unless more than one mixture event had occurred in the family, so we created a simulated hybrid genome that represents a first-generation mixture between an ancient Egyptian and an ancient Lebanese and tested whether SFI-44 could be modeled as descending from a mixture between SFI-43 and the hybrid genome. The model showed that SFI-44 derived ∼50% of his ancestry from SFI-43 and ∼50% from an individual whose ancestry was similar to that of the hybrid genome (Table S6). Thus, these results suggest that SFI-43 was an Egyptian woman and SFI-44 was her son from a man who himself had both Egyptian and Lebanese ancestries. The structure of this family in Lebanon highlights population movements and the heterogeneous society that existed at that time, but additional sampling is needed if we are to understand whether this cross-cultural mixing was common or whether our samples were exceptional. We removed SFI-43 and SFI-44 from all following analyses in which local individuals were grouped to represent their respective time periods.

**Figure 2:**
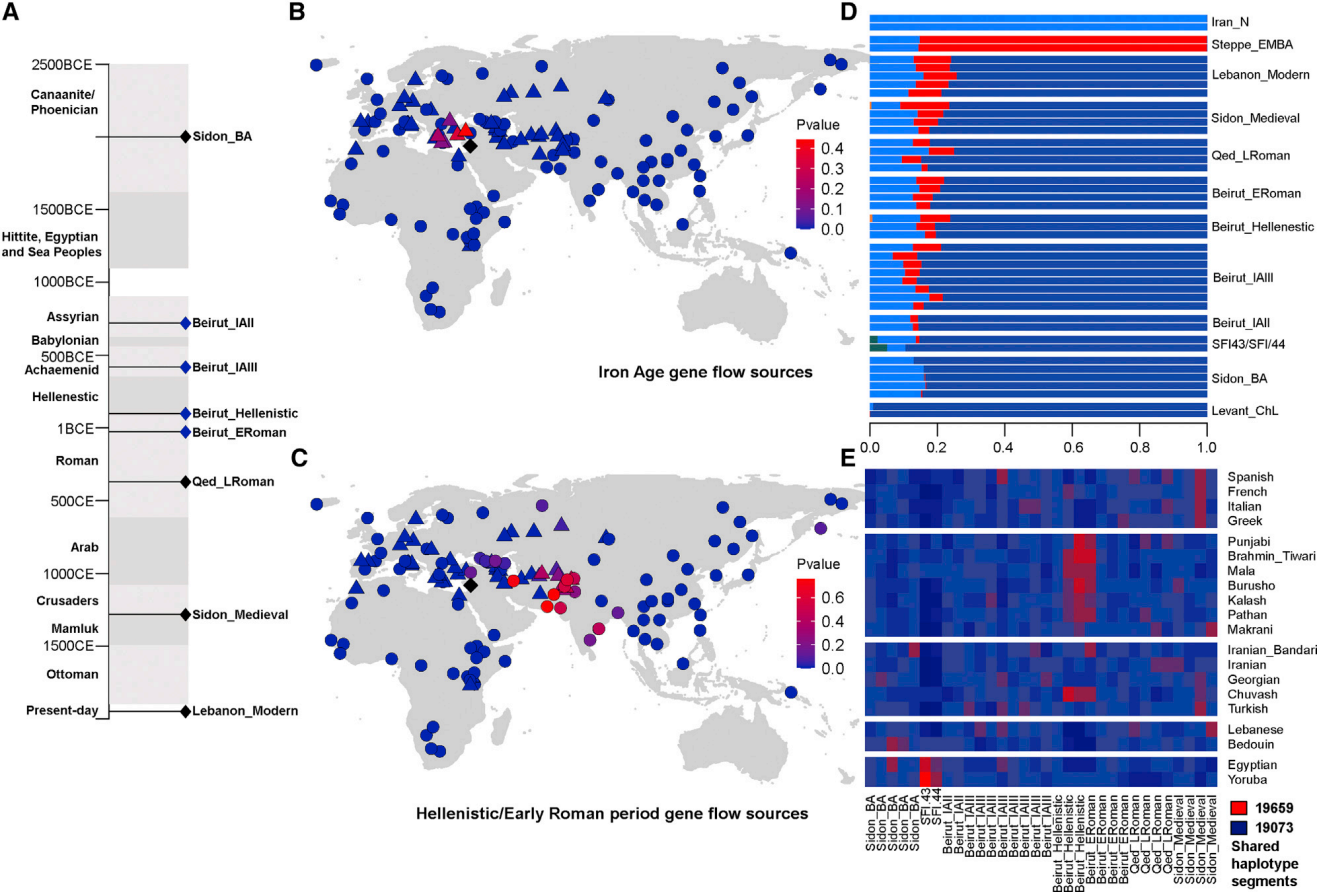
Admixture in Ancient Lebanon (A) Historical context of the studied samples. Horizontal lines indicate the time period of a sampled population, and the blue lozenges represent newly sequenced samples. (B and C) Locations of the source populations we used in *qpAdm* to test for admixture at the Iron Age (B) and the Hellenistic/early Roman period (C). The black lozenge on each map shows Lebanon’s location. Points represent modern populations in the dataset, whereas triangles represent ancient populations. Increased intensity of the red color indicates a higher p value for the model involving the source population (this should not be interpreted as an indication of the best model). We set the p values of the models that can be rejected to zero. (D) A DyStruct run with 166,693 transversions found in set 1 across nine time points. We show the plot of K = 6, which reveals an ancestral component (red) related to the Bronze Age Steppe population appearing in the Near East after the Bronze Age. (E) Haplotype segments shared between the ancient Lebanese and global modern populations. The heatmap is based on ChromoPainter’s co-ancestry matrix, and we averaged values from the modern populations over all individuals in the population. We scaled the heatmap by row to highlight the differences between the ancient individuals. Two Hellenistic individuals and one early Roman individual showed excess haplotype sharing with Central and South Asian populations compared with that of other ancient Lebanese individuals, whereas individuals SFI-43 and SFI-44 shared more segments with Africans and Egyptians. We counted between 19,073 (blue) and 19,659 (red) shared haplotype chunks in the dataset.

Having genetic representation from eight consecutive time periods (Figure 2A), we were able to test whether two populations that were successive in time formed a clade and derived all of their ancestry from a shared ancestral population or whether subsequent admixture had occurred and the two populations consequently lost their clade relationship. We started by computing *f4*-statistics of the form *f4(Lebanon Period1, Lebanon Period2; Ancient, Chimpanzee)*, in which a result significantly different from zero could indicate that genetic changes related to “Ancient” (an ancient population in our dataset) have occurred between two successive periods in Lebanon. We found that significant genetic changes that were marked by an increase in Eurasian ancestry related to ancient Europeans and ancient Central Asians occurred after the Bronze Age and starting from the Iron Age II (Figure S7A). We did not observe significant genetic differences between the Iron Age II and Iron Age III populations in this test (Figure S7B), and thus, we merged our samples from these two periods into one population (Figure S7C) and used *qpAdm* (see Supplemental Methods) to explore possible Iron Age admixture models (Tables 2 and S7). We found that the Lebanese Iron Age population can be modeled as a mixture of the local Bronze Age population (63%–88%) and a population related to ancient Anatolians or ancient South-Eastern Europeans (12%–37%) (Table 2 and Figure 2B). We replicated these results by running DyStruct^44^ with 166,693 transversions present in set 1 and showed that a Steppe-like ancestry, typically found in Europeans, appears in the Near East starting from the Iron Age II (Figure 2D). A potential source of this exogenous ancestry could be the Sea Peoples, a seafaring group of people with a disputed origin who attacked the Eastern Mediterranean and Egypt after the Bronze Age (1200–900 BCE). One of our successful models for admixture involved an ancestry source related to the Ashkelon (a city situated ∼170 miles south of the Beirut sites) Iron Age I population, which was previously identified as possibly descending from Sea-Peoples-related admixture.^18^ In addition, according to ancient Egyptian texts and archaeology, the Sea Peoples conquered the Levant but failed to conquer the Egyptians. Therefore, we tested whether the Eurasian gene flow to Lebanon during the Iron Age had also reached ancient Egypt by quantifying the Steppe ancestry in both regions at that time and found *f4(Sidon_BA, Beirut_IAII; Steppe_EMBA, Chimp)* is significantly negative (*Z* score = −4.13), but *f4(Sidon_BA, Egypt_prePtolemaic; Steppe_EMBA, Chimp)* has a value not significantly different from zero (*Z* score = 0.317), suggesting that either ancient Egypt did not receive the Eurasian gene flow that the Levant received during the Iron Age or that the Eurasian ancestry was replaced in Egypt as in Ashkelon, where in contrast to the Beirut_IAII, the European-related ancestry was no longer significant in the Ashkelon Iron Age II population.^18^ Additional Iron Age samples from the Levant coast and Egypt could reveal whether the Iron Age admixture had a north to south cline as a result of the location of the source populations or from differences in the scale of the successful migrations to the north or south of the Levant during this period.

**Table 2:** Modeling Populations from the Iron Age and Antiquity as a Mixture of the Preceding Population, A, and Any Global Ancient Population, B

**Test**	**A**	**B**	**p Value for Rank = 1**	**A**	**B**	**Std. Error**
				**Mixture Proportions**		
Beirut_IA	Sidon_BA	Anatolia_MLBA	4.44 × 10^−01^	0.63	0.37	0.06
Beirut_IA	Sidon_BA	Ashkelon_IAI	4.29 × 10^−01^	0.69	0.31	0.05
Beirut_IA	Sidon_BA	Anatolia_EBA	3.38 × 10^−01^	0.80	0.20	0.03
Beirut_IA	Sidon_BA	Mycenaean	2.17 × 10^−01^	0.77	0.23	0.04
Beirut_IA	Sidon_BA	Minoan_Odigitria	1.32 × 10^−01^	0.80	0.20	0.04
Beirut_HER	Beirut_IA	Butkara_H	4.93 × 10^−01^	0.92	0.08	0.01
Beirut_HER	Beirut_IA	Aligrama2_IA	4.46 × 10^−01^	0.93	0.07	0.01
Beirut_HER	Beirut_IA	Indus_Periphery	3.88 × 10^−01^	0.93	0.07	0.01
Beirut_HER	Beirut_IA	Swat_H	3.24 × 10^−01^	0.92	0.08	0.01
Beirut_HER	Beirut_IA	SPGT_IA	2.65 × 10^−01^	0.93	0.07	0.01

We show the top five models for each test based on their p value for the rank = 1 matrix. A p value > 0.05 indicates the model cannot be rejected. We removed infeasible models with negative proportions from the table. Beirut_IA included individuals from the Iron Age II and Iron Age III periods and can be modeled as a mixture of the local Bronze Age population and a population related to ancient Anatolians or ancient South-Eastern Europeans. Beirut_HER included individuals from the Hellenistic and early Roman periods and can be modeled as a mixture of the local population Beirut_IA and an ancient Central and South Asian population.

The second genetic change in ancient Lebanon can be observed during the Hellenistic and early Roman periods. We merged individuals from these two periods into one population (Beirut_HER) because several individuals had overlapping radiocarbon dates and the *f4*-statistics showed symmetry between the Beirut_Hellenistic and Beirut_ERoman populations (Figure S8). We found that the Hellenistic and early Roman population can be modeled as a mixture of the local population, Beirut_IA (88%–94%), and a Central/South Asian population (6%–12%) (Tables 2 and S8 and Figure 2C). We then analyzed haplotype segments shared between the ancient Lebanese and modern populations in set 2 by using ChromoPainter^44^ on 2.5 million imputed SNPs and found that two Hellenistic individuals (SFI-5 and SFI-12) and one early Roman individual (SFI-11) had excess haplotype sharing with Central and South Asians (Figures 2E and S9), thus confirming the *qpAdm* results. The relationship of ancient Lebanon with Central and South Asia also manifests in the presence of haplogroup L1a1-M27 among the modern Lebanese Y chromosome lineages (Figure S10). Haplogroup L1a1-M27 is common today in Central and South Asia but rare elsewhere (in the 1000 Genomes Project,^45^ this lineage was found exclusively in Sri Lankan Tamil from the UK [STU], Punjabi from Lahore, Pakistan [PJL], Indian Telugu from the UK [ITU], Gujarati Indian from Houston, Texas [GIH], and Bengali from Bangladesh [BEB]). We tested^46^ (see Supplemental Methods) the coalescence of the five L1a1-M27 Lebanese chromosomes and found that they all derived from a man who lived around 450 BCE–50 CE, a time interval overlapping with the Hellenistic period (Figure S10). The presence of the Central/South Asian ancestry in Lebanon during the Hellenistic period mirrors the connected geography under the rule of Alexander the Great’s empire, which had also assimilated the Achaemenid Empire that preceded it and thus maintained a connection between the West and East for five centuries. These large contiguous empires thus facilitated the movement and mixture of people as seen directly by the Egyptian-Lebanese family and the admixed individuals reported here who lived in the Near East at that time.

We next tested the genetic changes between the Hellenistic/early Roman period and the late Roman period (Qed_LRoman) and found little genetic differences from the *f4*-statistics (Figure S11), which is notable because during this period there was significant population movement between the Near East and Europe, as identified from the genomes of ancient Near Easterners found in Rome at that time.^16^ When we model Qed_LRoman as a mixture of the Hellenestic/early Roman period population and another ancient population, we find successful models involving ancient Anatolians and South-Eastern Europeans (Table S9). However, because this ancestry was already present in Lebanon starting from the Iron Age, its excess in Qed_LRoman could be from population structure, especially because the Qed_LRoman samples were from a remote mountainous region, whereas the Hellenistic/early Roman samples were from the coast, and in addition, we found that the admixture models were not significant when Beirut_IA was used as the source of the local ancestry, showing that Qed_LRoman derived all of its ancestry from preceding local populations (Table S9).

From the late Roman period to the medieval period, we detect an increase in African ancestry (Figure S11B), but that increase remains slightly below statistical significance (*Z* score = −2.4) and accounts for ∼2.9% of Lebanon_Medieval’s ancestry when ancient East Africans are used in the admixture model (Table S10). The final genetic change observed in Lebanon occurred after the Crusaders’ period but, as we showed previously,^4^ was not related to the Crusaders themselves. We found^4^ an increase in ancestry related to populations from the Caucasus and Turks in the modern Lebanese population after the medieval period (Figure S11C and Table S11). Using admixture-induced linkage disequilibrium (LD) decay,^47^^,^^48^ we show that admixture occurred around 1640–1740 CE when Lebanon was under Ottoman rule (Figure S12). The LD-decay test also detects significant admixture that occurred during the Hellenistic period, which is consistent with our more direct inferences from the ancient individuals analyzed here (Figure S12).

**Figure 3:**
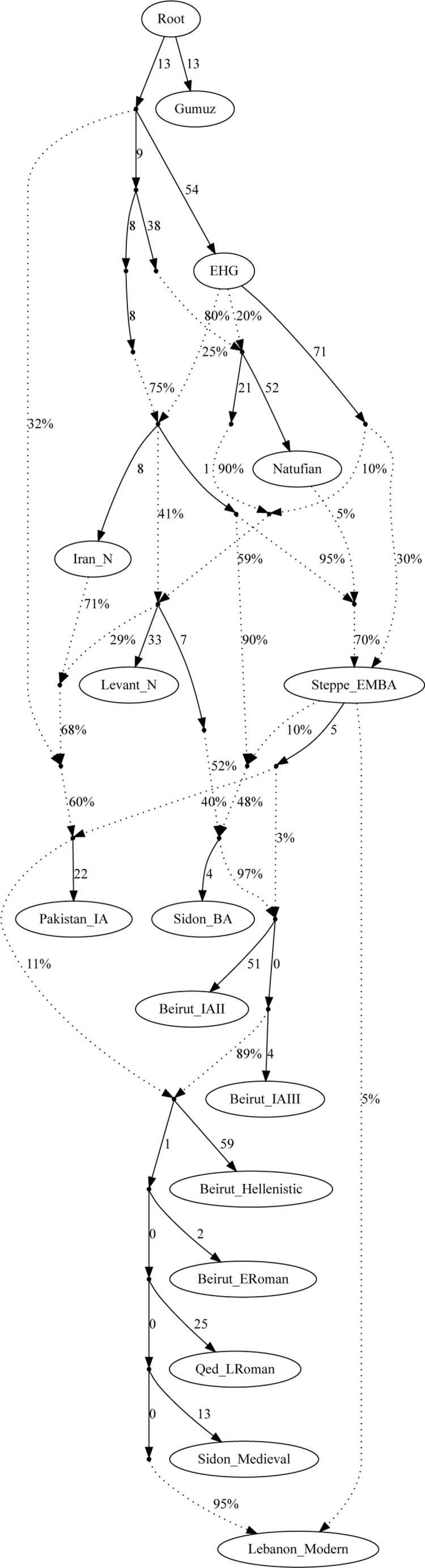
An Admixture Graph Model for Ancient Lebanon A graph model that fits our data showing the relationship between the ancient Lebanon populations and the admixture events that contributed to the population until modern times. Worst *f4*-statistics, Iran_N,Levant_N;EHG,Qed_LRoman; *Z* score = 3.0. See Figure S13 for alternative graph models.

Finally, we fit all the ancient and modern Lebanese data into an admixture graph model showing their relationship with other ancient populations by using data in set 2*.* The graph supports the results reported here, showing substantial genetic continuity in Lebanon since the Bronze Age interrupted by three significant admixture events during the Iron Age, Hellenistic period, and Ottoman period, each contributing 3%–11% of non-local ancestry to the admixed population (Figures 3 and S13).

In this study, we present new whole-genome sequence data from ancient individuals who lived in the Near East between the Iron Age and the Roman period, spanning a time marked by major historical events and population movements. Our data capture the genetic outcome of some of these events but also show that the genetic composition of the general population was minimally affected and that great cultural transitions in the Near East were not in these cases matched by comparable genetic transitions. Yet, we show that the small genetic changes we detect when using ancient populations sampled from a time series have the power to provide information about past events with details that complement the available historical records.

## Declaration of Interests

The authors declare no competing interests.

## Data and Code Availability

Raw sequencing reads for the ancient individuals are available through the European Nucleotide Archive (ENA) under accession number ENA: ERP121575. Aligned sequences, genotypes, and imputed genotypes can be obtained from the corresponding author M.H.

## Web Resources

European Nucleotide Archive (ENA), https://www.ebi.ac.uk/ena
International Society of Genetic Genealogy (ISOGG), https://isogg.org

## Supplemental Information

Document S1. Figures S1–S13, Tables S1–S11, and Supplemental Methods

Table S12 Information on Ancient Individuals Analyzed in this Study

Document S2. Article plus Supplemental Information
