# Supplementary material for: A Genetic History of the Near East from an aDNA Time Course Sampling Eight Points in the Past 4,000 Years
Source: Am J Hum Genet. 2020 May 28;107(1):149–57. doi: 10.1016/j.ajhg.2020.05.008 (PMC7332655; doi:10.1016/j.ajhg.2020.05.008)
Supplement: Document S1. Figures S1–S13, Tables S1–S11, and Supplemental Methods [file mmc1.pdf]

The American Journal of Human Genetics, Volume *107*

Supplemental Data

A Genetic History of the Near East from an aDNA Time Course Sampling Eight Points in the Past 4,000 Years

Marc Haber, Joyce Nassar, Mohamed A. Almarri, Tina Saupe, Lehti Saag, Samuel J. Griffith, Claude Doumet-Serhal, Julien Chanteau, Muntaha Saghieh-Beydoun, Yali Xue, Christiana L. Scheib, and Chris Tyler-Smith

Supplemental Figures

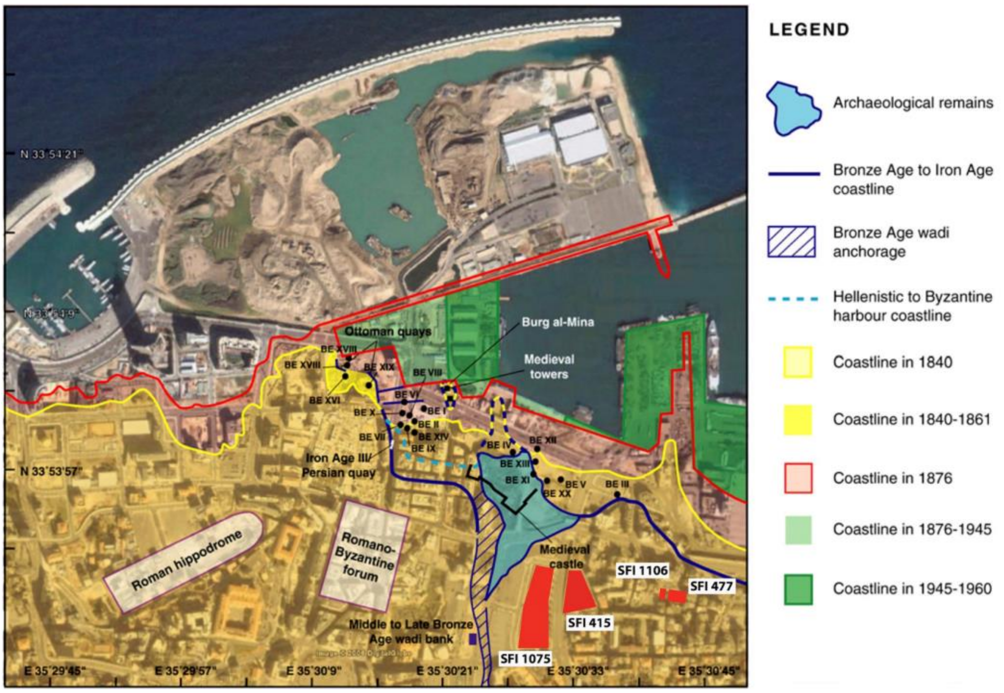

Figure S1. Archaeological parcels location. Saifi 477, 1106, 1075 and 514 are parcels located east of the Central District of modern Beirut which revealed burial sites from the 9th century BCE (Iron Age II) till the 2nd century CE (Roman period). These sites belong to the vast cemeteries of ancient Beirut extending to the south and east and revealing thousands of burials excavated since the 1990's. Iron Age II burials (9th-7th century BCE) excavated in Saifi 415 with samples SFI-55 and SFI-56 extracted from two individuals: one adult (7854) and one subadult (7855) buried in the same burial (T43). Iron Age III burials (6th- 5th BCE) were found partly in Saifi 1075, located few hundred meters to the east of where the settlement was found (west of the Martyrs' square).[1] Ten samples were taken from nine burials: SFI-34 (T5), SFI-35 (T6), SFI-36 (T8), SFI-39 (T12), SFI-42 (T21), SFI-43 (T24), SFI-44 (context 1593), SFI-45 (T25), SFI-47 (T27), SFI-50 (T38). Seven burials ranging from the 2nd BCE till the 2nd CE (Hellenistic-Roman periods) were excavated in two adjacent sites (Saifi 477 and Saifi 1106) 300m east of Saifi 1075. Figure modified from Marriner et al.[2]

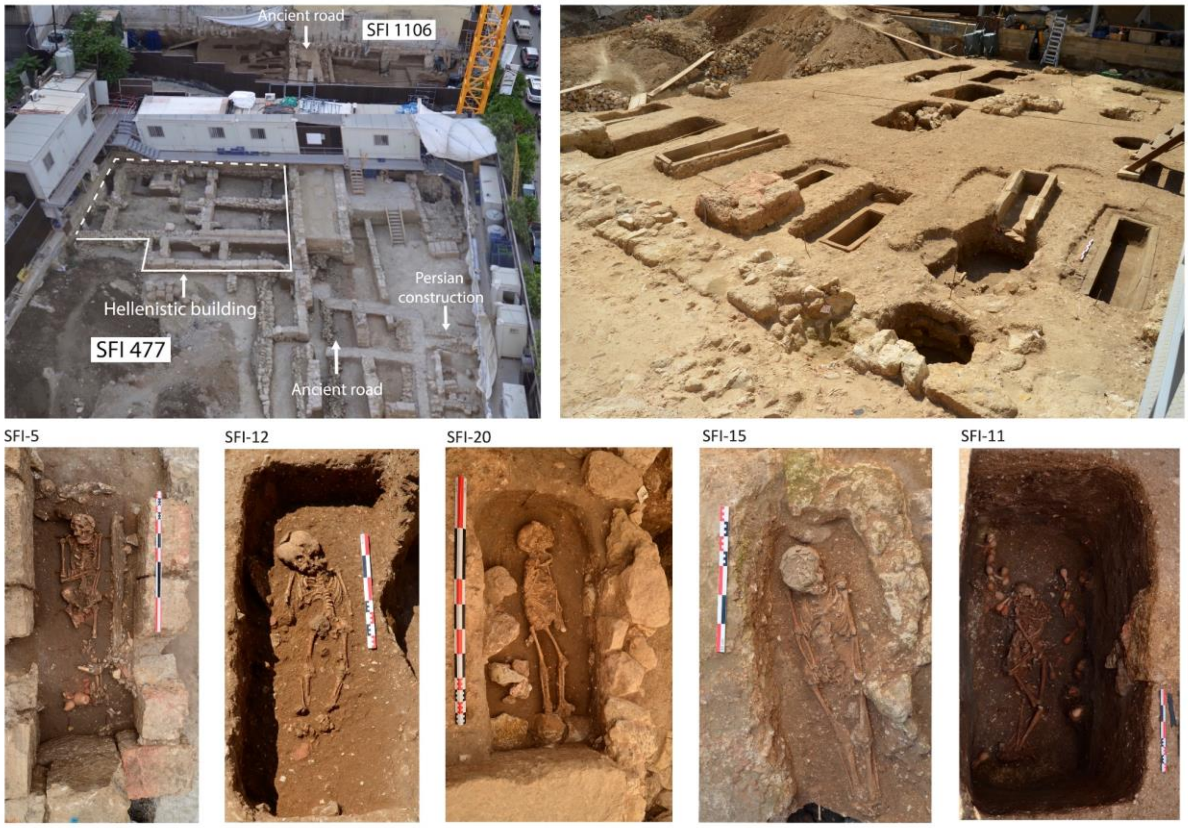

Figure S2. View of parcels Saifi 477 and Saifi 1106. Seven burials ranging from the 2nd BCE till the 2nd CE (Hellenistic-Roman periods) were excavated in two adjacent sites (Saifi 477 and Saifi 1106) 300m east of Saifi 1075. On those sites, the burials were dug along a road used from the Iron Age III (Persian period) till the late Roman period where architectures from the corresponding periods were found. The burials' architecture varies between simple pits dug in soil, simple pits with aligned stones on the edges, or stone built burials. They contain one individual placed on the back and in an extended position, either on a north-south/south-north axis east or on an east-west/west-east axis. In some cases, the deceased is accompanied by funerary material composed of pottery vessels or/and ornamental objects (earrings). The use of wooden containers is evident from conserved remains or iron nails.

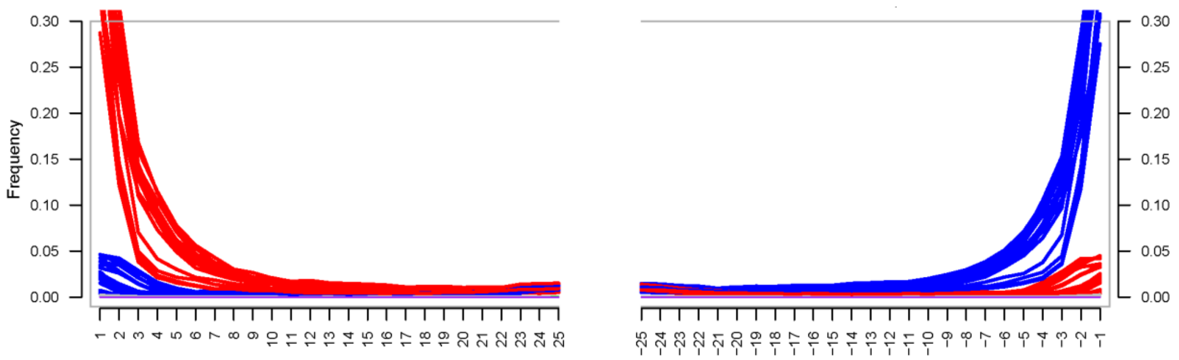

Figure S3. Post-mortem damage patterns.[3] Base substitutions C>T from the 5’ (left) and G>A from the 3’ end (right) show patterns typical of ancient DNA damage for all samples sequenced in this study.

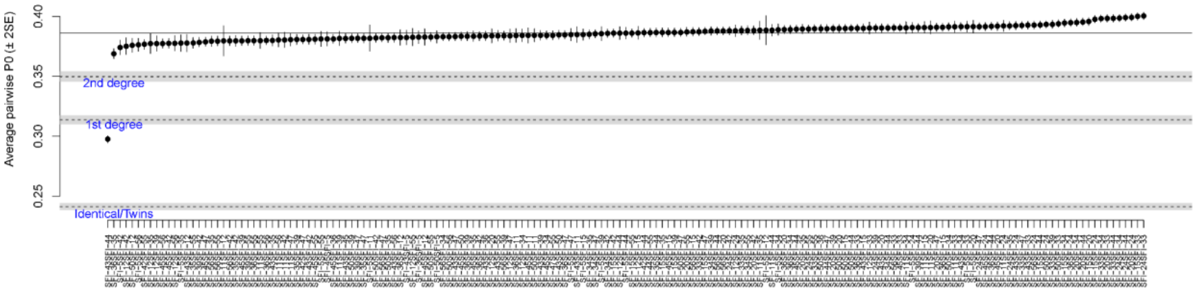

Figure S4. Relationship estimation for the ancient Lebanon samples. We used READ[4] with default parameters on the ancient Lebanon samples in the *Set1* dataset. Two individuals SFI-43 and SFI-44 were identified as first degree relatives.

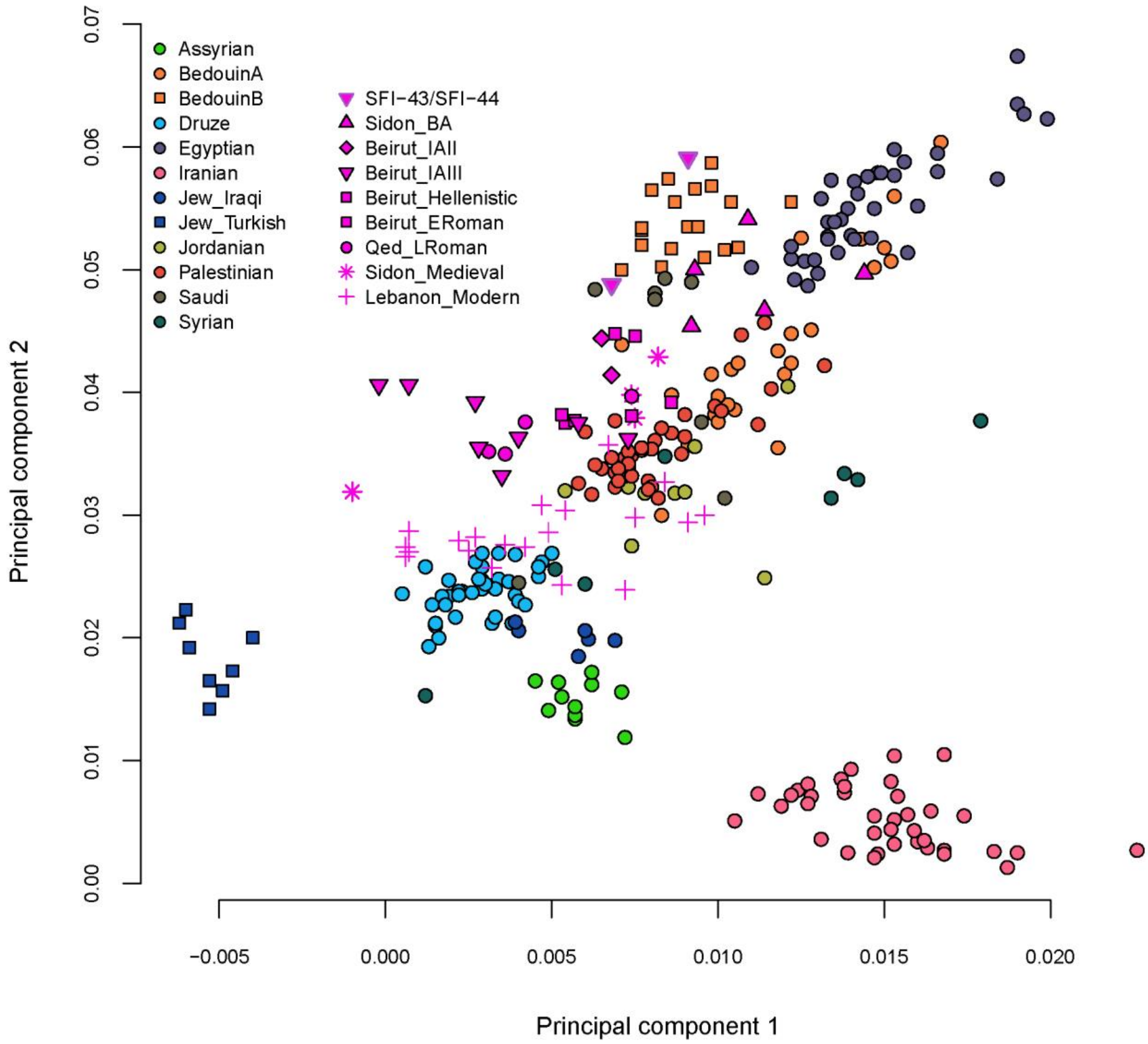

Figure S5. Principal Components Analysis (PCA) showing ancient Lebanon in the context of present-day Near Easterners. PC values for the Near Easterners were extracted from the PCA shown in Figure 1.

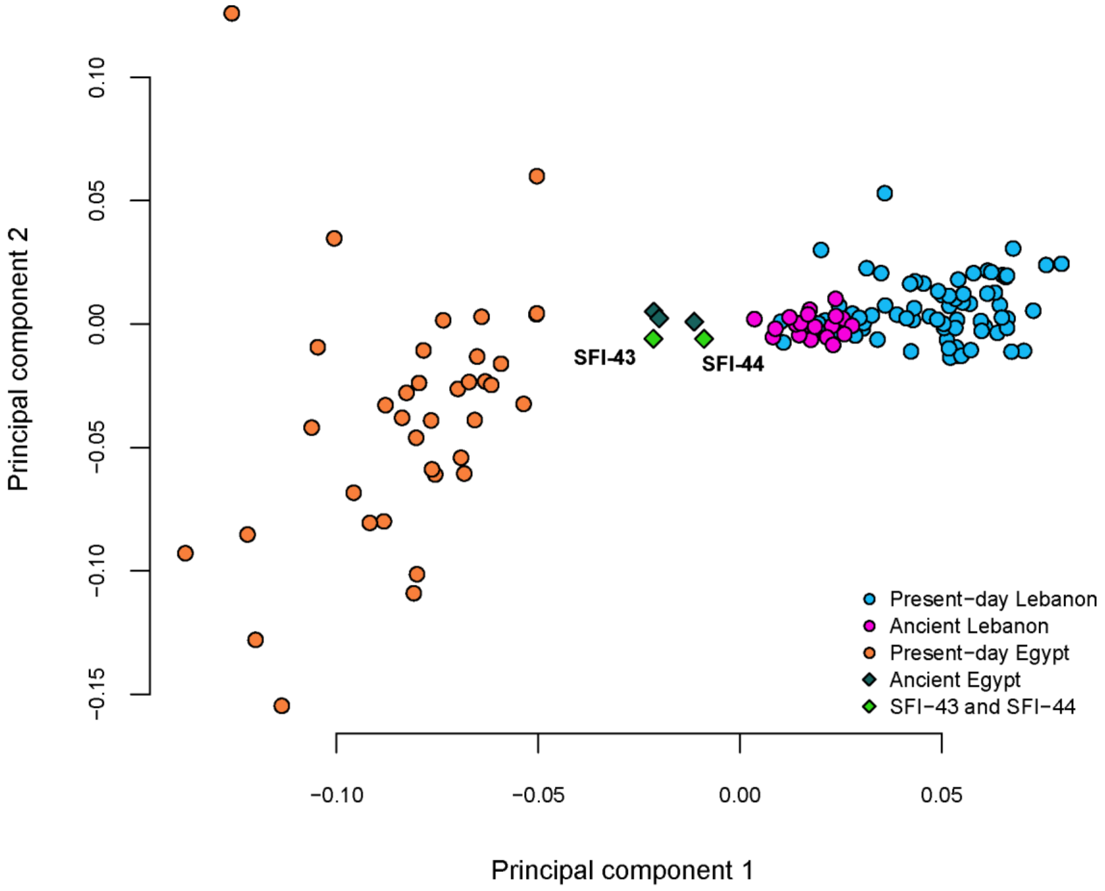

Figure S6. PCA of Egyptians and Lebanese. Individuals SFI-43 and SFI-44 cluster with ancient Egyptians but SFI-44 is closer to ancient individuals from Lebanon.

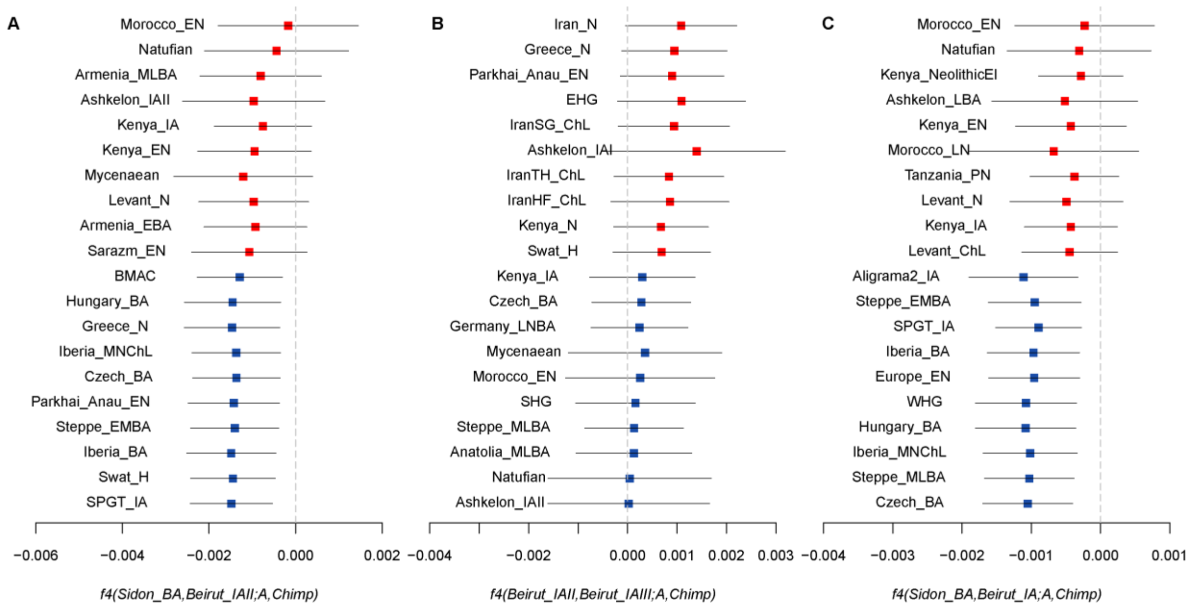

Figure S7. Genetic changes in Lebanon between the Bronze Age and the Iron Age. (A) Comparing the Bronze Age population with the Iron Age II population, (B) the Iron Age II population with the Iron Age III population, and (C) the Bronze Age population with the Iron Age II/Iron Age III populations merged into one group. In this figure and the following we plot the statistic *f4*(Period1,Period2;Ancient,Chimpanzee) and ±3 standard errors from results with the 10 lowest (blue) and 10 highest (red) Z-scores.

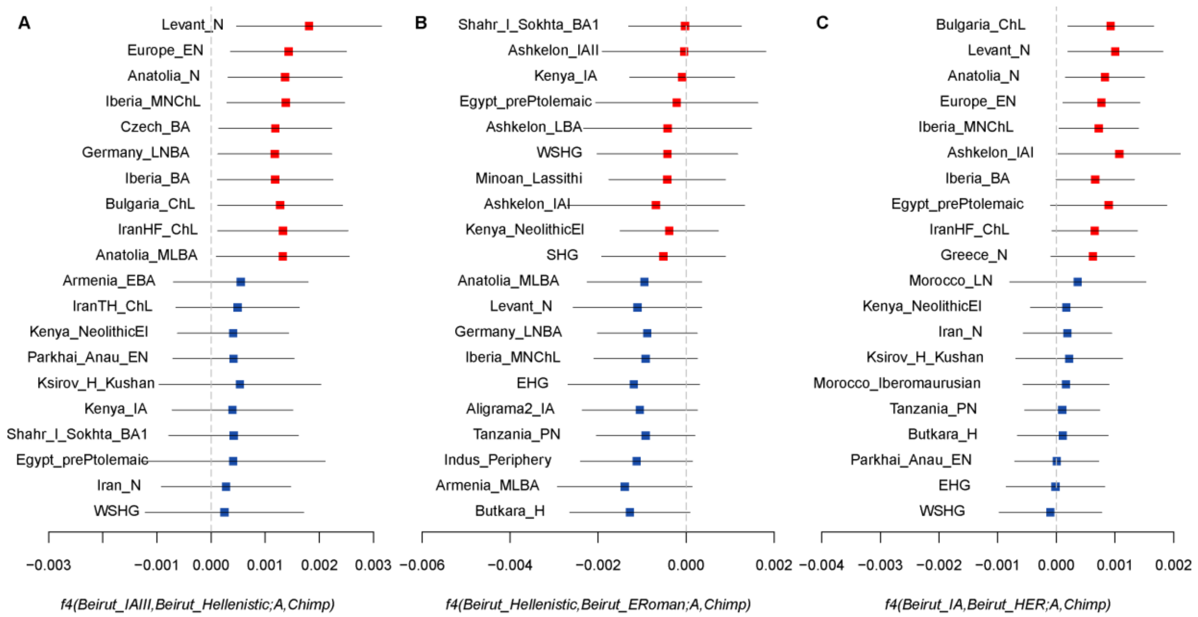

Figure S8. Genetic changes in Lebanon between the Iron Age and the Hellenistic/Early Roman periods. (A) Comparing the Iron Age III population with the Hellenistic period population, (B) the Hellenistic with the Early Roman period, and (C) the Iron Age (merged populations of Beirut_IAII and Beirut_IAIII) with Beirut_HER (merged populations of Beirut_Hellenistic and Beirut_ERoman).

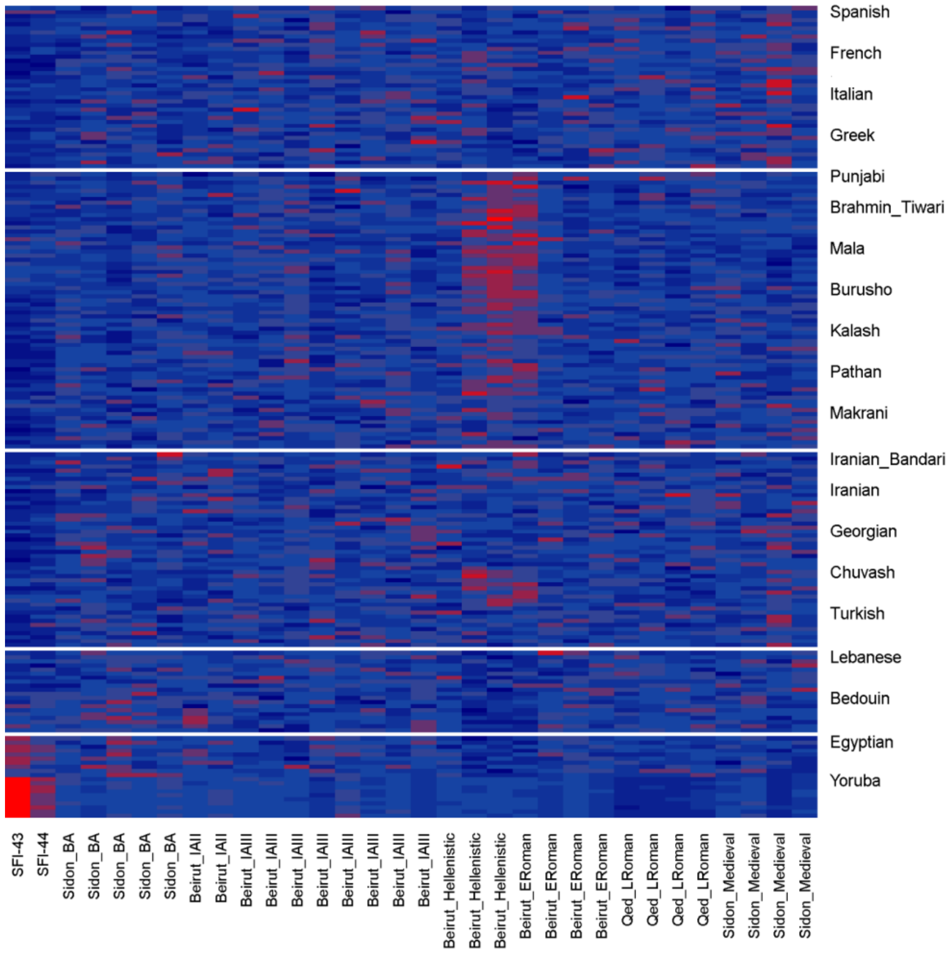

Figure S9. Haplotype sharing between ancient Lebanese and present-day populations. Plot similar to Figure 2E but showing results from the individuals in the reference populations rather than the population average.

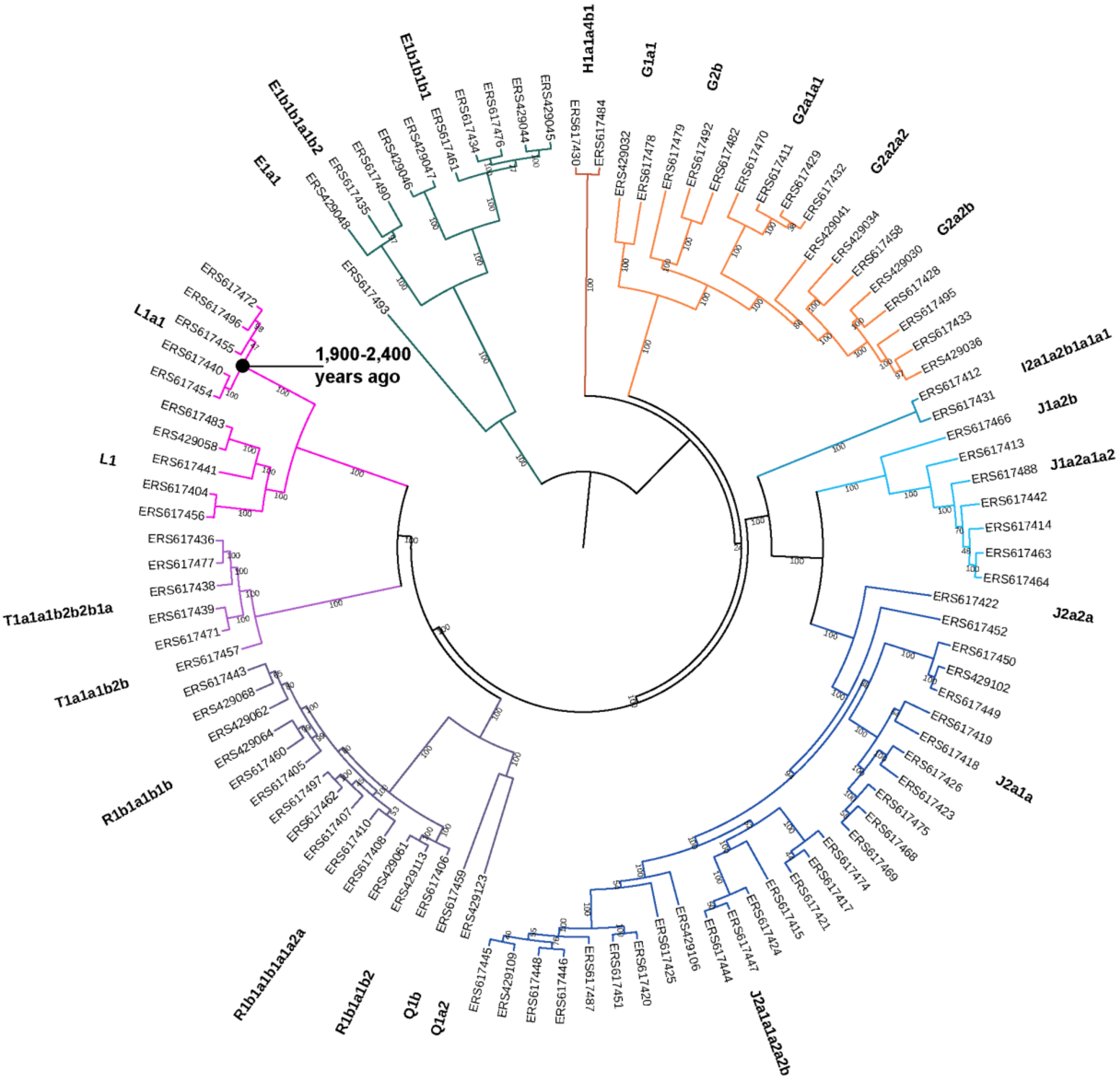

Figure S10. Y Chromosome phylogenetic tree from Lebanese males. A maximum-likelihood tree of 99 Y chromosome sequences from Lebanon. Different branch colors represent different haplogroups annotated according to the International Society of Genetic Genealogy (ISOGG V 14.255). We found that the Central/South Asian Y Chromosomes L1a1 present in Lebanon coalesced 1,900-2,400 years ago during the Hellenistic period when admixture with a Central/South Asian population can also be detected from genome-wide inferences.

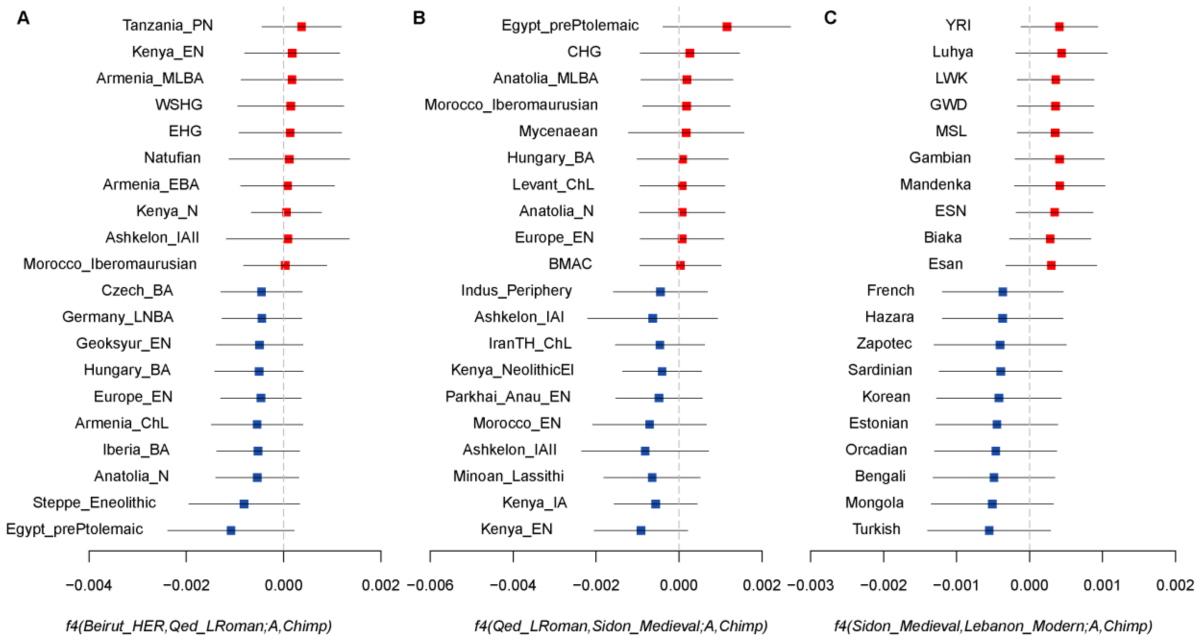

Figure S11. Genetic changes in Lebanon between the Hellenistic/Early Roman periods and the present-day. (A) Comparing the Hellenistic/Early Roman period population with the Late Roman period population, (B) the Late Roman period with the medieval period, and (C) changes between the medieval period and the present-day.

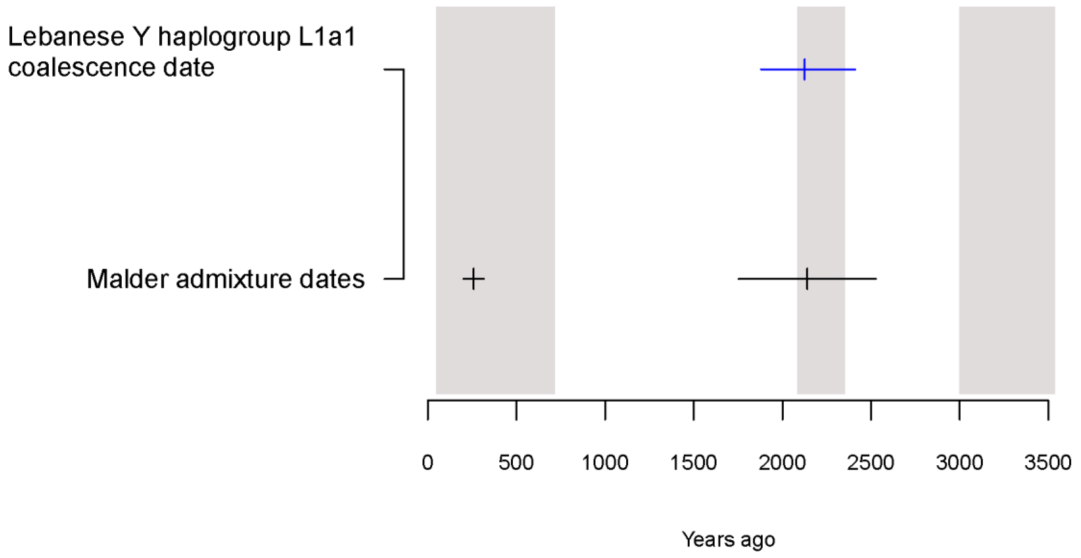

Figure S12. Admixture signals using modern and ancient Lebanese. Grey shades indicate the time period when admixture is directly detected (Table 2 and S11) from a sampled population. Blue cross shows coalescence time of the L1a1 Y chromosomes in Lebanon. Black crosses show admixture time estimated from decay of linkage disequilibrium (LD).

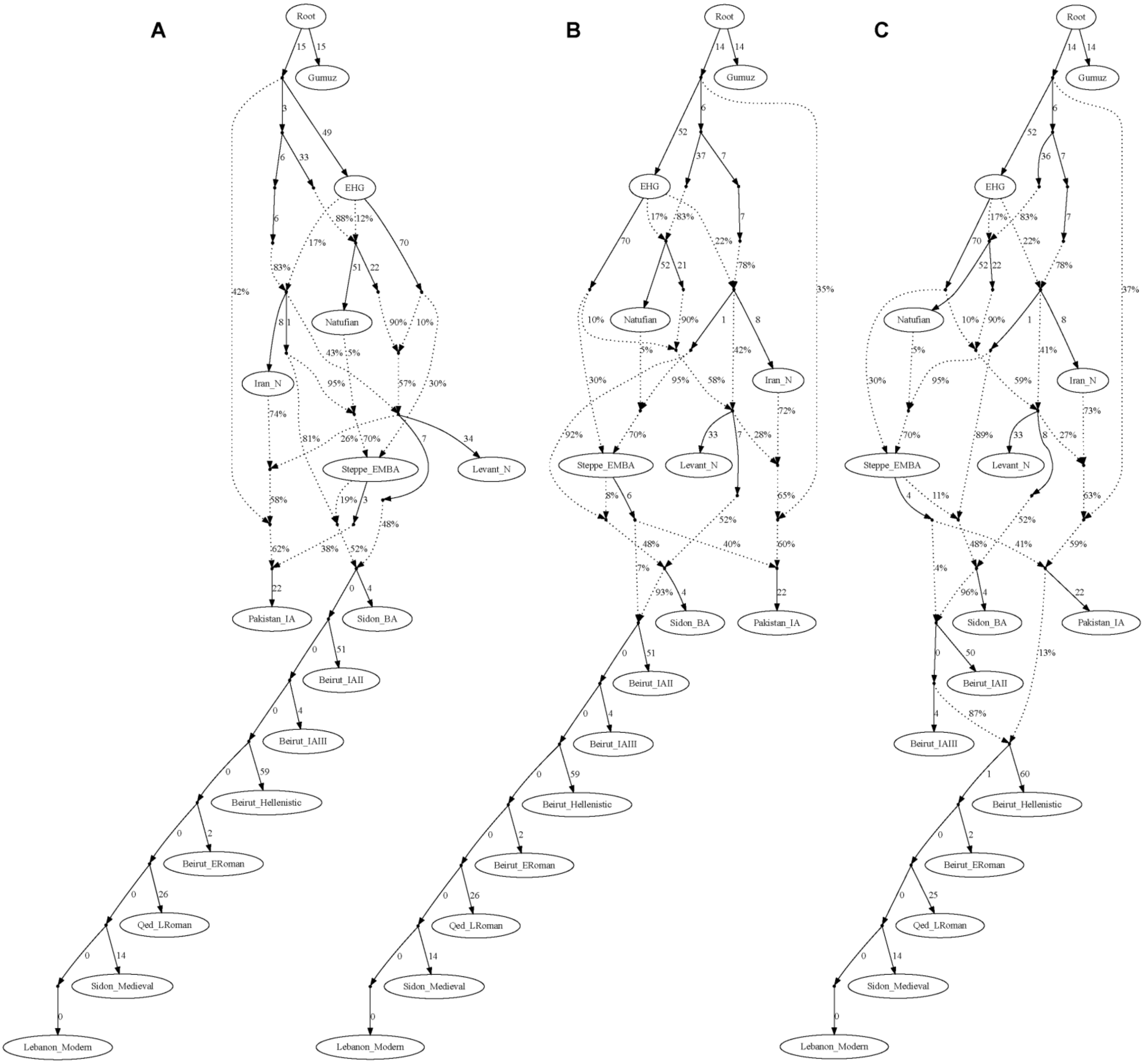

Figure S13. qpGraph models showing possible population relationships in ancient Lebanon. The admixture graph model with three limited admixtures in ancient Lebanon (main text Figure 3) has a worst Z-score=3.0 for the difference between estimated and fitted f-statistics. (A) A model with full genetic continuity in Lebanon after the Bronze Age has worst Z-score=9.5. (B) Adding an admixture event during the Iron Age II reduces the Z-score to 8.2. (C) Adding a second admixture event during the Hellenistic period reduces the Z-score to 4.0.

Supplemental Tables

ENA number	ID	Analyzis ID	Excavation Site	Burial ID	Period	Date (cal)	Civilization	Study
ERS1790729	SI-23	Sidon_BA	Sidon	46	Middle Bronze Age	-	Canaanite	Haber et al. 2017[5]
ERS1790730	SI-09	Sidon_BA	Sidon	65	Middle Bronze Age	-	Canaanite	Haber et al. 2017
ERS1790731	SI-21	Sidon_BA	Sidon	75	Middle Bronze Age	-	Canaanite	Haber et al. 2017
ERS1790732	SI-12	Sidon_BA	Sidon	63	Middle Bronze Age	-	Canaanite	Haber et al. 2017
ERS1790733	SI-22	Sidon_BA	Sidon	54	Middle Bronze Age	1950-1730BCE	Canaanite	Haber et al. 2017
ERS4542976	SFI-56	Beirut_IAII	Beirut SFI-415	T43 cxt 7854	Iron Age II	-	Assyrian	This study
ERS4542991	SFI-55	Beirut_IAII	Beirut SFI-415	T43 cxt 7855	Iron Age II	-	Assyrian	This study
ERS4542962	SFI-43	Beirut_IAIII	Beirut SFI-1075	T24 cxt 1592	Iron Age III	-	Achaemenid	This study
ERS4542967	SFI-50	Beirut_IAIII	Beirut SFI-1075	T38 cxt 5534	Iron Age III	-	Achaemenid	This study
ERS4542969	SFI-36	Beirut_IAIII	Beirut SFI-1075	T8	Iron Age III	-	Achaemenid	This study
ERS4542989	SFI-42	Beirut_IAIII	Beirut SFI-1075	T21	Iron Age III	540BCE-396BCE	Achaemenid	This study
ERS4542964	SFI-45	Beirut_IAIII	Beirut SFI-1075	T25 cxt 5249	Iron Age III	-	Achaemenid	This study
ERS4542984	SFI-34	Beirut_IAIII	Beirut SFI-1075	T5 cxt 1062	Iron Age III	-	Achaemenid	This study
ERS4542983	SFI-35	Beirut_IAIII	Beirut SFI-1075	T6 cxt 1055	Iron Age III	-	Achaemenid	This study
ERS4542988	SFI-39	Beirut_IAIII	Beirut SFI-1075	T12	Iron Age III	-	Achaemenid	This study
ERS4542990	SFI-44	Beirut_IAIII	Beirut SFI-1075	cxt 1593	Iron Age III	-	Achaemenid	This study
ERS4542987	SFI-47	Beirut_IAIII	Beirut SFI-1075	T27 cxt 2542	Iron Age III	-	Achaemenid	This study
ERS4542979	SFI-20	Beirut_Hellenistic	Beirut SFI-477	T42	Hellenistic	199BCE-37BCE	Hellenistic	This study
ERS4542972	SFI-5	Beirut_Hellenistic	Beirut SFI-477	T9	Hellenistic	234BCE-92BCE	Hellenistic	This study
ERS4542974	SFI-12	Beirut_Hellenistic	Beirut SFI-477	T27	Hellenistic	209BCE-89BCE	Hellenistic	This study
ERS4542980	SFI-24	Beirut_ERoman	Beirut SFI-1106	T8	Early Roman	55BCE-58CE	Roman	This study
ERS4542982	SFI-33	Beirut_ERoman	Beirut SFI-1106	T18	Early Roman	48CE-222CE	Roman	This study
ERS4542973	SFI-11	Beirut_ERoman	Beirut SFI-477	T24	Early Roman	119BCE-27CE	Roman	This study
ERS4542977	SFI-15	Beirut_ERoman	Beirut SFI-477	T32	Early Roman	176BCE-3CE	Roman	This study
ERS3189333	QED-2	Qed_LRoman	Qornet ed-Deir	QED	Late Roman	244-400CE	Roman	Haber et al.2019[6]
ERS3189335	QED-4	Qed_LRoman	Qornet ed-Deir	QED	Late Roman	426-632CE	Roman	Haber et al.2019
ERS3189338	QED-7	Qed_LRoman	Qornet ed-Deir	QED	Late Roman	237-389CE	Roman	Haber et al.2019
ERS3189342	QED-12	Qed_LRoman	Qornet ed-Deir	QED	Late Roman	-	Roman	Haber et al.2019
ERS3189349	SI-38	Sidon_Medieval	Sidon	110	Medieval	-	Crusaders	Haber et al.2019
ERS3189353	SI-42	Sidon_Medieval	Sidon	110	Medieval	1154-1281CE	Crusaders	Haber et al.2019
ERS3189348	SI-44	Sidon_Medieval	Sidon	101	Medieval	-	Crusaders	Haber et al.2019
ERS3189355	SI-45	Sidon_Medieval	Sidon	110	Medieval	1219-1278CE	Crusaders	Haber et al.2019

Table S1. Ancient samples from Lebanon analyzed in this study

ID	Contamination % estimated from Male X
SFI-55	2.32±1.1
SFI-42	1.6±1.1
SFI-45	0.61±0.8
SFI-34	0.86±0.3
SFI-35	1.56±1.3
SFI-39	1.09±0.7
SFI-44	1.32±0.4
SFI-47	1.37±0.4
SFI-5	2.78±3.9
SFI-12	3.81±3.8
SFI-11	2.37±3
SFI-15	0.93±0.3

Table S2. Contamination estimates from males' X chromosome. Using ANGSD[7] with options -b 2700000 -c 154900000 -d 2 -m 0 -f 1. Showing results from Method1 new llh version.[8]

ID	Contamination estimate (low-high) from mtDNA
SFI-56	0.01 (0-0.02)
SFI-55	0.01 (0-0.02)
SFI-43	0.01 (0-0.02)
SFI-50	0.01 (0-0.02)
SFI-36	0.01 (0-0.02)
SFI-42	0 (0-0.01)
SFI-45	0 (0-0.01)
SFI-34	0.01 (0-0.02)
SFI-35	0.01 (0-0.02)
SFI-39	0.01 (0-0.02)
SFI-44	0 (0-0.01)
SFI-47	0.01 (0-0.02)
SFI-20	0.01 (0-0.02)
SFI-5	0.01 (0-0.02)
SFI-12	0.01 (0-0.02)
SFI-24	0.01 (0-0.02)
SFI-33	0.01 (0-0.02)
SFI-11	0.01 (0-0.02)
SFI-15	0.01 (0-0.02)

Table S3. Contamination estimates from mtDNA. Using schmutzi[9] with the --uselength option.

ID	Period	Sex (genetic)	Y Haplogroup	MT Haplogroup
SFI-56	Iron Age II	female	-	U1a1a
SFI-55	Iron Age II	male	J	H2a
SFI-43	Iron Age III	female	-	T2c1+146
SFI-50	Iron Age III	female	-	U1a
SFI-36	Iron Age III	female	-	R0a1a
SFI-42	Iron Age III	male	J1-M267	H2a
SFI-45	Iron Age III	male	J-M304	T2a1b1
SFI-34	Iron Age III	male	J1-M267	T1a2
SFI-35	Iron Age III	male	I2a1b-M436	R0a1a
SFI-39	Iron Age III	male	H2-P96	I1b
SFI-44	Iron Age III	male	J1a2a1a2-P58	T2c1+146
SFI-47	Iron Age III	male	G2a2a1a2-L91	W6
SFI-20	Hellenistic	female	-	H41
SFI-5	Hellenistic	male	Q1b-M346	K1a5a
SFI-12	Hellenistic	male	E1b1b1a1a2-V65	H14a
SFI-24	Early Roman	female	-	H8b
SFI-33	Early Roman	female	-	T1
SFI-11	Early Roman	male	G2a2b-L30	N1b1
SFI-15	Early Roman	male	G2a2b1a2-M3302	I1c1

Table S4. Ancient samples' sex and uniparental haplogroups.

A	B	P value for rank=0
SFI-43	Egypt_prePtolemaic	7.63E-02
SFI-43	SFI-44	1.24E-02
SFI-43	Egypt_Ptolemaic	1.14E-03
SFI-43	Ashkelon_IAII	1.03E-09
SFI-43	Beirut_Hellenistic	5.71E-10
SFI-44	SFI-43	1.24E-02
SFI-44	Egypt_Ptolemaic	2.12E-03
SFI-44	Ashkelon_IAII	1.84E-03
SFI-44	Egypt_prePtolemaic	2.09E-04
SFI-44	Beirut_Hellenistic	1.73E-04

Table S5. Test for affinity of individuals SFI-43 and SFI-44 to a population *B* in the dataset. We show the top 5 results for each individual based on the P value for rank=0 and highlight in red the instances where the P value is >0.05 indicating *A* forms a clade with *B*.

				Mixture proportions		
**Test**	**A**	**B**	**P value for rank=1**	**A**	**B**	**Std. Error**
SFI-44	SFI-43	Beirut_IAIII	6.87E-01	0.73	0.27	0.07
SFI-44	SFI-43	Sidon_BA	6.48E-01	0.71	0.29	0.08
SFI-44	SFI-43	Ashkelon_IAII	6.23E-01	0.60	0.39	0.10
SFI-44	SFI-43	Qed_LRoman	5.67E-01	0.73	0.27	0.07
SFI-44	SFI-43	Beirut_IAII	4.99E-01	0.68	0.32	0.09
SFI-44	SFI-43	*hybrid*	5.98E-01	0.49	0.51	0.1

Table S6. Modelling SFI-44 as a mixture of SFI-43 and a population *B*. We report the top 5 models and the model involving a *hybrid* genome constructed by mixing an ancient Egyptian (JK2888) and an individual (SFI-34) from the Beirut_IAIII population. A P value > 0.05 (red) indicates the model cannot be rejected.

Test	A	B	P value for rank=1	Mixture proportions A	Mixture proportions B	Std. Error
Beirut_IA	Sidon_BA	Sardinian	1.23E-02	0.91	0.09	0.02
Beirut_IA	Sidon_BA	Tuscan	3.83E-03	0.90	0.10	0.03
Beirut_IA	Sidon_BA	Basque	3.18E-03	0.93	0.07	0.02
Beirut_IA	Sidon_BA	Cretan	1.87E-03	0.87	0.14	0.04
Beirut_IA	Sidon_BA	French	1.85E-03	0.94	0.06	0.02

Table S7. Iron Age admixture proportions when *B* is a modern population. In this table and following tables we show the top 5 models based on the P value for rank=1 and highlight in red the instances where the P value is >0.05 indicating the model cannot be rejected.

Test	A	B	P value for rank=1	Mixture proportions A	Mixture proportions B	Std. Error
Beirut_HER	Beirut_IA	Makrani	7.61E-01	0.89	0.11	0.02
Beirut_HER	Beirut_IA	Brahui	7.59E-01	0.90	0.10	0.02
Beirut_HER	Beirut_IA	Iranian	7.15E-01	0.84	0.17	0.03
Beirut_HER	Beirut_IA	Balochi	7.02E-01	0.91	0.10	0.02
Beirut_HER	Beirut_IA	Kalash	6.04E-01	0.92	0.08	0.02

Table S8. Hellenistic/Early Roman period admixture proportions when *B* is a modern population.

Test	A	B	P value for rank=1	Mixture proportions		
				A	B	Std. Error
Qed_LRoman	Beirut_HER	Anatolia_EBA	9.89E-02	0.86	0.14	0.06
Qed_LRoman	Beirut_HER	Greece_Minoan_Lassithi	9.42E-02	0.91	0.10	0.04
Qed_LRoman	Beirut_HER	Anatolia_MLBA	8.54E-02	0.74	0.26	0.12
Qed_LRoman	Beirut_HER	Greece_Mycenaean	7.53E-02	0.85	0.15	0.06
Qed_LRoman	Beirut_HER	Greece_N	6.92E-02	0.93	0.07	0.03
Qed_LRoman	Beirut_Iron	Anatolia_EBA	1.05E-02	1.01	-0.01	0.07
Qed_LRoman	Beirut_Iron	Greece_Minoan_Lassithi	2.18E-02	1.07	-0.07	0.04
Qed_LRoman	Beirut_Iron	Anatolia_MLBA.SG	1.23E-02	1.08	-0.08	0.21
Qed_LRoman	Beirut_Iron	Greece_Mycenaean	3.09E-02	1.07	-0.07	0.03
Qed_LRoman	Beirut_Iron	Greece_N	1.29E-02	1.07	-0.07	0.09

Table S9. Modelling the Late Roman period population as a mixture of the local populations preceding in time and an ancient population *B*.

Test	A	B	P value for rank=1	Mixture proportions		
				A	B	Std. Error
Sidon_Medieval	Qed_LRoman	Kenya_N	3.47E-02	0.98	0.03	0.01
Sidon_Medieval	Qed_LRoman	Kenya_NeolithicEl	3.40E-02	0.98	0.02	0.01
Sidon_Medieval	Qed_LRoman	Kenya_EN	3.38E-02	0.97	0.03	0.01
Sidon_Medieval	Qed_LRoman	Tanzania_PN	3.09E-02	0.98	0.02	0.01
Sidon_Medieval	Qed_LRoman	Kenya_IA	2.41E-02	0.98	0.02	0.01

Table S10. Modelling the Lebanon medieval population as a mixture of the local Late Roman period population and an ancient population *B*.

Test	A	B	P value for rank=1	Mixture proportions		
				A	B	Std. Error
Lebanon_Modern	Sidon_Medieval	Abkhasian	1.81E-01	0.90	0.10	0.03
Lebanon_Modern	Sidon_Medieval	Armenian	1.68E-01	0.83	0.17	0.06
Lebanon_Modern	Sidon_Medieval	North Ossetian	1.12E-01	0.93	0.07	0.03
Lebanon_Modern	Sidon_Medieval	Adygei	9.29E-02	0.93	0.07	0.03
Lebanon_Modern	Sidon_Medieval	Turkish	7.43E-02	0.91	0.09	0.04

Table S11. Modelling the Lebanon modern population as a mixture of the local medieval period population and a modern population *B*.

Supplemental Methods

Sequences processing and genotyping

We processed the new sequences using the PALEOMIX[10] pipeline retaining reads ≥30 bp and collapsing pairs with minimum overlap of 15 bp while allowing a mismatch rate of 1/3 between the pairs. We mapped the merged sequences with BWA-backtrack v0.7.15[11] to the hs37d5 reference sequence, removed duplicates, and removed bases from the end of the reads until the frequency of nucleotide misincorporation estimated with mapDamage v2.0.6-2-g6507525[3] dropped to below 5%. We used ANGSD v0.925-21-g5de79b5[7] to randomly sample a single sequence with a minimum base quality of ≥20 to represent each SNP.

Datasets

We created two datasets for the analysis by merging the new data with published data. *Set1* included ancient individuals (Table S12) extracted from a previously merged dataset of genomes available from the Reich lab https://reich.hms.harvard.edu/downloadable-genotypes-present-day-and-ancient-dna-data-compiled-published-papers (v42.4). We additionally extracted from this dataset modern genomes from South Asia,[12] from worldwide populations described in the 1000 Genomes Project,[13] and from the Simons Genome Diversity Project (SGDP).[14] We added individuals from ancient and modern Lebanon[5; 6] and modern Egypt and Ethiopia.[15] From Medieval Lebanon, we only analysed individuals who represented the local ancestry:[6] SI-38, SI-42, SI-44, and SI-45 and removed the outlier SI-44 when samples where pooled into the Sidon_Medieval group.

We merged the datasets using the mergeit program available from the EIGENSOFT package v7.2.1[16] with options docheck: YES and strandcheck: YES. We filtered out sex-linked and triallelilic SNPs and sites that were outside the 1000 Genomes Project's strict mask resulting in a dataset of 2012 modern humans and 914 ancient individuals with 815,791 SNPs. In addition, we created *Set2* which consisted of modern individuals from worldwide populations genotyped on the Human Origins array[17-19] merged with the ancient individuals to obtain a dataset of 2788 modern humans and 914 ancient individuals with 539,766 SNPs.

Ancient samples' sex and uniparental haplogroups

We determined the sex of the samples from the ratio of sequences aligning to the X and Y chromosomes.[20] We genotyped the Y chromosome of the ancient males jointly with the 1000 Genomes Project[13] and modern Lebanese[5] males using freebayes v1.3.1[21] with options --report-monomorphic --ploidy 1 --min-base-quality 20 --min-mapping-quality 30 and restricted the calling to 10.3 Mb of the Y chromosome previously determined to be accessible to short-read sequencing.[22] We

determined the Y haplogroup using yHaplo[23] and according to ISOGG v14.255 annotations. We determined the mtDNA haplogroup by uploading the BAM files the mtDNA server.[24]

Y Chromosome phylogeny and dating

We extracted Y chromosome genotypes of modern Lebanese from the jointly called dataset described in the previous section. We then inferred a maximum likelihood phylogeny using RAxML v8.2.10[25] with arguments -m ASC_GTRGAMMA and --asc-corr=stamatakis, using variable sites with QUAL ≥1. We selected the tree with the best likelihood from 100 runs and replicated it 1000 times for bootstrap values. We determined the nodes' ages using the ρ statistic[26] and defined the ancestral state of a site according to our previous study[27] by assigning alleles as ancestral when they were monomorphic in the nine samples belonging to the A and B haplogroups in the SGDP dataset. We then determined the age of the L1a1 node as follows: The ancestral node comprised two clades, we select one sample from each clade (for example ERS617454 and ERS617455) and divide the number of derived variants found in the first sample but absent from the second by the total number of sites having the ancestral state in both samples. We repeat for all possible pairs under the L1a1 node and report the average value of divergence times in units of years by applying a point mutation rate of 0.76×10^{-9} ($0.67\text{-}0.86 \times 10^{-9}$ 95%CI) mutations per site per year.[28]

Principal Component analysis and DyStruct

We used smartpca v16000 from the EIGENSOFT package[16] to compute a PCA using parameters numoutlieriter: 0, lsqproject: YES, autoshrink: YES and using only variation in modern populations selected to represent genetic diversity in Central Asia, the Near East, and Europe.

We run DyStruct with default arguments and using 166,693 transversions found in *Set1* across nine time points binned as follows (in years ago): 14,500-10,000; 10,000-8000; 8000-6000; 6000-5200; 5200-5000; 5000-3000; 3000-1400;1400-200; and present-day.

***f4* statistics**

We used *qpDstat* v755 from the ADMIXTOOLS package[17] with parameter f4mode: YES to test genetic continuity in Lebanon using significant (±3 standard errors) deviation from zero in the statistic *f4*(Period1,Period2,A,Chimpanzee) as indicating a possible genetic change between two subsequent periods related to A (any ancient population in our Set1 dataset). We exclude from the test populations with single individuals or with overlapping number of SNPs <200,000. When testing genetic change in Lebanon_Modern we choose A to be any modern population in our Set1 dataset.

qpWave/qpAdm analysis

SFI-43 and SFI-44

We used *qpWave* v410 and *qpAdm* v810[29; 30] from the ADMIXTOOLS package[17] with option allsnps:YES to determine if SFI-43 and SFI-44 formed a clade with any ancient population in *Set1* and if SFI-44 can be modelled as a mixture of ancestries related to SFI-43 and any other ancient individual or population in our dataset. We selected 11 outgroups that are related differently to the ancient population in our dataset: Ust'-Ishim (a 45,000-year-old Siberian), Eastern hunter-gatherers from Russia (EHG), Sweden hunter-gatherers (SHG), Caucasus hunter-gatherers (CHG), Morocco Iberomaurusian, Levant Natufian, Levant Chalcolithic, Anatolia Neolithic, Iran Neolithic, in addition to modern populations Han and Mbuti.

Admixture in ancient Lebanon

We used *qpAdm* to model ancient Lebanon populations as a mixture of two streams of ancestries, the first source related to the local population which preceded the tested population in time and the second source deriving from a population found in our *Set1* dataset. We used 19 outgroups:
Ust'-Ishim, Mota (a 4,500-year-old from Ethiopia), EHG, SHG, CHG, Western hunter-gatherers from Europe (WHG), West Siberian hunter-gatherers (WSHG), Morocco Early Neolithic, Levant Natufian, Levant Chalcolithic, Anatolia Neolithic, Europe Early Neolithic, Germany Late Neolithic/Bronze Age, Iberia Middle Neolithic/Chalcolithic, Iran Neolithic, Iran Chalcolithic (Tepe Hissar), Turkmenistan Neolithic (Geoksyur), Mbuti and Han. We also rotate these outgroups and test them as a second source of ancestry while keeping the remaining 18 populations in the outgroup set.

Genotype imputation and ChromoPainter analysis

We used ChromoPainter[31] and followed the method described by Antonio et al.[32] who showed that common variants (>1% MAF in the 1000 Genomes Project) in low-coverage genomes (as low as 0.1x) can be imputed with relatively high accuracy in ancient European and Near Eastern samples using the 1000 Genomes Project reference panels. We used GATK UnifiedGenotyper[33] to estimate genotypes likelihoods from the ancient samples for common variants (>1% minor allele frequency) found in the 1000 Genomes Project reference panel[13] using parameters: min_base_quality_score 30 --output_mode EMIT_ALL_SITES --allSitePLs –alleles <reference_panel> --genotyping_mode GENOYTPE_GIVEN_ALLELES -R <hg19 reference fasta>. We then used Beagle v4.0[34] for imputation with parameters: gprobs=true, impute=true, gl=<UnifiedGenotyper output>, ref=<Beagle imputation reference panel>, map <GRCh37 recombination map>.
The Reference panel and recombination maps were downloaded from the Beagle website (https://faculty.washington.edu/browning/beagle/b4_1.html). We extracted a subset of 196 modern

human samples relevant to our study from the Human Origins dataset[17-19] and imputed them with the Michigan server (https://imputationserver.sph.umich.edu/index.html) using the 1000 Genomes Project phase 3 reference panel.[13] We then ran the ChromoPainter/finestructure v4.0.1[31] inference pipeline with default settings outputting a co-ancestry matrix with values depicting copies of haplotype segments shared between individuals.

MALDER

We used MALDER v1.0[35; 36] with parameters mindis: 0.005, binsize: 0.0005 and a generation time of 30 years to estimate admixture time in the modern population from decay of linkage disequilibrium (LD). We used the modern populations in *Set2* as proxies for the admixing ancestry sources.

qpGraph

We used *qpGraph* v6450[17] to draw a phylogenetic model that tests the models obtained from the *qpAdm* results. We started by a basic graph similar to the one we used to explain genetic relationships in the Near East[37] but using populations: Gumuz, Iran_N, EHG, Natufian, Levant_N, Pakistan_IA (Aligrama2_IA), Steppe_EMBA, Sidon_BA and Lebanon_Modern. This graph captures some of the previous knowledge on these population relationships, for example the Sidon_BA population deriving ~50% of its ancestry from the local population and ~50% from a population related to Iran_N.[5] However, we warn that our admixture graph is not intended to model the deep relationships between the ancient reference populations used here and is not a full representation of their complex admixture history. We used the *qpGraph* models to highlight a substantial genetic continuity in Lebanon interrupted by few admixture episodes and showed that it fits the models obtained from *qpAdm*. We added to the above basic set of populations Beirut_IAII, Beirut_IAIII, Beirut_Hellenistic, Beirut_ERoman, Qed_LRoman, and Sidon_Medieval. We found that the graph showing complete genetic continuity in Lebanon and without admixture after the Bronze Age has several outlier f-statistic results from unaccounted-for relationships between the Lebanese populations and other ancient populations (Z-score=9.5) (Figure S13A). We added the first admixture event observed in the *qpAdm* results during the Iron Age II from a Steppe-related population, this slightly improved the Z-scores (Figure S13B). We then added a Central Asian related admixture to the Hellenistic population and further improved the Z-scores (Figure S13C) but remained with a worst Z-score=4 from an unaccounted-for relationship between the Steppe and Lebanon_Modern. We finally added an admixture edge to Lebanon_Modern and obtained a worst f-statistic Z-score=3.0 (main text Figure 3).

Supplemental References

1. Elayi, J., and Sayegh, H. (1998). Un quartier du port phénicien de Beyrouth au Fer III - Perse.(Paris: Gabalda).
2. Marriner, N., Morhange, C., and Saghieh-Beydoun, M. (2008). Geoarchaeology of Beirut's ancient harbour, Phoenicia. Journal of Archaeological Science 35, 2495-2516.
3. Jonsson, H., Ginolhac, A., Schubert, M., Johnson, P.L., and Orlando, L. (2013). mapDamage2.0: fast approximate Bayesian estimates of ancient DNA damage parameters. Bioinformatics 29, 1682-1684.
4. Monroy Kuhn, J.M., Jakobsson, M., and Gunther, T. (2018). Estimating genetic kin relationships in prehistoric populations. PLoS One 13, e0195491.
5. Haber, M., Doumet-Serhal, C., Scheib, C., Xue, Y., Danecek, P., Mezzavilla, M., Youhanna, S., Martiniano, R., Prado-Martinez, J., Szpak, M., et al. (2017). Continuity and Admixture in the Last Five Millennia of Levantine History from Ancient Canaanite and Present-Day Lebanese Genome Sequences. Am J Hum Genet 101, 274-282.
6. Haber, M., Doumet-Serhal, C., Scheib, C.L., Xue, Y., Mikulski, R., Martiniano, R., Fischer-Genz, B., Schutkowski, H., Kivisild, T., and Tyler-Smith, C. (2019). A Transient Pulse of Genetic Admixture from the Crusaders in the Near East Identified from Ancient Genome Sequences. Am J Hum Genet 104, 977-984.
7. Korneliussen, T.S., Albrechtsen, A., and Nielsen, R. (2014). ANGSD: Analysis of Next Generation Sequencing Data. BMC Bioinformatics 15, 356.
8. Rasmussen, M., Guo, X., Wang, Y., Lohmueller, K.E., Rasmussen, S., Albrechtsen, A., Skotte, L., Lindgreen, S., Metspalu, M., Jombart, T., et al. (2011). An Aboriginal Australian genome reveals separate human dispersals into Asia. Science 334, 94-98.
9. Renaud, G., Slon, V., Duggan, A.T., and Kelso, J. (2015). Schmutzi: estimation of contamination and endogenous mitochondrial consensus calling for ancient DNA. Genome Biol 16, 224.
10. Schubert, M., Ermini, L., Der Sarkissian, C., Jonsson, H., Ginolhac, A., Schaefer, R., Martin, M.D., Fernandez, R., Kircher, M., McCue, M., et al. (2014). Characterization of ancient and modern genomes by SNP detection and phylogenomic and metagenomic analysis using PALEOMIX. Nat Protoc 9, 1056-1082.
11. Li, H., and Durbin, R. (2009). Fast and accurate short read alignment with Burrows-Wheeler transform. Bioinformatics 25, 1754-1760.
12. Mondal, M., Casals, F., Xu, T., Dall'Olio, G.M., Pybus, M., Netea, M.G., Comas, D., Laayouni, H., Li, Q., Majumder, P.P., et al. (2016). Genomic analysis of Andamanese provides insights into ancient human migration into Asia and adaptation. Nat Genet 48, 1066-1070.
13. The 1000 Genomes Project Consortium. (2015). A global reference for human genetic variation. Nature 526, 68-74.
14. Mallick, S., Li, H., Lipson, M., Mathieson, I., Gymrek, M., Racimo, F., Zhao, M., Chennagiri, N., Nordenfelt, S., Tandon, A., et al. (2016). The Simons Genome Diversity Project: 300 genomes from 142 diverse populations. Nature 538, 201-206.
15. Pagani, L., Schiffels, S., Gurdasani, D., Danecek, P., Scally, A., Chen, Y., Xue, Y., Haber, M., Ekong, R., Oljira, T., et al. (2015). Tracing the route of modern humans out of Africa by using 225 human genome sequences from Ethiopians and Egyptians. Am J Hum Genet 96, 986-991.
16. Patterson, N., Price, A.L., and Reich, D. (2006). Population structure and eigenanalysis. PLoS Genet 2, e190.
17. Patterson, N., Moorjani, P., Luo, Y., Mallick, S., Rohland, N., Zhan, Y., Genschoreck, T., Webster, T., and Reich, D. (2012). Ancient admixture in human history. Genetics 192, 1065-1093.
18. Lazaridis, I., Patterson, N., Mittnik, A., Renaud, G., Mallick, S., Kirsanow, K., Sudmant, P.H., Schraiber, J.G., Castellano, S., Lipson, M., et al. (2014). Ancient human genomes suggest three ancestral populations for present-day Europeans. Nature 513, 409-413.

19. Lazaridis, I., Nadel, D., Rollefson, G., Merrett, D.C., Rohland, N., Mallick, S., Fernandes, D., Novak, M., Gamarra, B., Sirak, K., et al. (2016). Genomic insights into the origin of farming in the ancient Near East. Nature 536, 419-424.
20. Skoglund, P., Stora, J., Gotherstrom, A., and Jakobsson, M. (2013). Accurate sex identification of ancient human remains using DNA shotgun sequencing. Journal of Archaeological Science 40, 4477-4482.
21. Garrison, E., and Marth, G. (2012). Haplotype-based variant detection from short-read sequencing. arXiv preprint arXiv:1207.3907 [q-bio.GN].
22. Poznik, G.D., Henn, B.M., Yee, M.C., Sliwerska, E., Euskirchen, G.M., Lin, A.A., Snyder, M., Quintana-Murci, L., Kidd, J.M., Underhill, P.A., et al. (2013). Sequencing Y chromosomes resolves discrepancy in time to common ancestor of males versus females. Science 341, 562-565.
23. Poznik, G.D. (2016). Identifying Y-chromosome haplogroups in arbitrarily large samples of sequenced or genotyped men. bioRxiv.
24. Weissensteiner, H., Forer, L., Fuchsberger, C., Schopf, B., Kloss-Brandstatter, A., Specht, G., Kronenberg, F., and Schonherr, S. (2016). mtDNA-Server: next-generation sequencing data analysis of human mitochondrial DNA in the cloud. Nucleic Acids Res 44, W64-69.
25. Stamatakis, A. (2014). RAxML version 8: a tool for phylogenetic analysis and post-analysis of large phylogenies. Bioinformatics 30, 1312-1313.
26. Forster, P., Harding, R., Torroni, A., and Bandelt, H.J. (1996). Origin and evolution of Native American mtDNA variation: a reappraisal. Am J Hum Genet 59, 935-945.
27. Haber, M., Jones, A.L., Connell, B.A., Asan, Arciero, E., Yang, H., Thomas, M.G., Xue, Y., and Tyler-Smith, C. (2019). A Rare Deep-Rooting D0 African Y-Chromosomal Haplogroup and Its Implications for the Expansion of Modern Humans out of Africa. Genetics.
28. Fu, Q., Li, H., Moorjani, P., Jay, F., Slepchenko, S.M., Bondarev, A.A., Johnson, P.L., Aximu-Petri, A., Prufer, K., de Filippo, C., et al. (2014). Genome sequence of a 45,000-year-old modern human from western Siberia. Nature 514, 445-449.
29. Haak, W., Lazaridis, I., Patterson, N., Rohland, N., Mallick, S., Llamas, B., Brandt, G., Nordenfelt, S., Harney, E., Stewardson, K., et al. (2015). Massive migration from the Steppe was a source for Indo-European languages in Europe. Nature 522, 207-211.
30. Reich, D., Patterson, N., Campbell, D., Tandon, A., Mazieres, S., Ray, N., Parra, M.V., Rojas, W., Duque, C., Mesa, N., et al. (2012). Reconstructing Native American population history. Nature 488, 370-374.
31. Lawson, D.J., Hellenthal, G., Myers, S., and Falush, D. (2012). Inference of population structure using dense haplotype data. PLoS Genet 8, e1002453.
32. Antonio, M.L., Gao, Z., Moots, H.M., Lucci, M., Candilio, F., Sawyer, S., Oberreiter, V., Calderon, D., Devitofranceschi, K., Aikens, R.C., et al. (2019). Ancient Rome: A genetic crossroads of Europe and the Mediterranean. Science 366, 708-714.
33. DePristo, M.A., Banks, E., Poplin, R., Garimella, K.V., Maguire, J.R., Hartl, C., Philippakis, A.A., del Angel, G., Rivas, M.A., Hanna, M., et al. (2011). A framework for variation discovery and genotyping using next-generation DNA sequencing data. Nat Genet 43, 491-498.
34. Browning, S.R., and Browning, B.L. (2007). Rapid and accurate haplotype phasing and missing-data inference for whole-genome association studies by use of localized haplotype clustering. Am J Hum Genet 81, 1084-1097.
35. Loh, P.R., Lipson, M., Patterson, N., Moorjani, P., Pickrell, J.K., Reich, D., and Berger, B. (2013). Inferring admixture histories of human populations using linkage disequilibrium. Genetics 193, 1233-1254.
36. Pickrell, J.K., Patterson, N., Loh, P.R., Lipson, M., Berger, B., Stoneking, M., Pakendorf, B., and Reich, D. (2014). Ancient west Eurasian ancestry in southern and eastern Africa. Proc Natl Acad Sci U S A 111, 2632-2637.

37. Haber, M., Saif-Ali, R., Al-Habori, M., Chen, Y., Platt, D.E., Tyler-Smith, C., and Xue, Y. (2019). Insight into the genomic history of the Near East from whole-genome sequences and genotypes of Yemenis. bioRxiv, 749341.
