# Supplementary material for: A Genetic History of the Near East from an aDNA Time Course Sampling Eight Points in the Past 4,000 Years
Source: Am J Hum Genet. 2020 May 28;107(1):149–57. doi: 10.1016/j.ajhg.2020.05.008 (PMC7332655; doi:10.1016/j.ajhg.2020.05.008)
Supplement: Document S2. Article plus Supplemental Information [file mmc3.pdf]

A Genetic History of the Near East from an aDNA Time Course Sampling Eight Points in the Past 4,000 Years

Marc Haber,[1,2,3,*] Joyce Nassar,[4] Mohamed A. Almarri,[3] Tina Saupe,[5,6] Lehti Saag,[5] Samuel J. Griffith,[5] Claude Doumet-Serhal,[7] Julien Chanteau,[8] Muntaha Saghieh-Beydoun,[9] Yali Xue,[3] Christiana L. Scheib,[5] and Chris Tyler-Smith[3,*]

The Iron and Classical Ages in the Near East were marked by population expansions carrying cultural transformations that shaped human history, but the genetic impact of these events on the people who lived through them is little-known. Here, we sequenced the whole genomes of 19 individuals who each lived during one of four time periods between 800 BCE and 200 CE in Beirut on the Eastern Mediterranean coast at the center of the ancient world's great civilizations. We combined these data with published data to traverse eight archaeological periods and observed any genetic changes as they arose. During the Iron Age (~1000 BCE), people with Anatolian and South-East European ancestry admixed with people in the Near East. The region was then conquered by the Persians (539 BCE), who facilitated movement exemplified in Beirut by an ancient family with Egyptian-Lebanese admixed members. But the genetic impact at a population level does not appear until the time of Alexander the Great (beginning 330 BCE), when a fusion of Asian and Near Easterner ancestry can be seen, paralleling the cultural fusion that appears in the archaeological records from this period. The Romans then conquered the region (31 BCE) but had little genetic impact over their 600 years of rule. Finally, during the Ottoman rule (beginning 1516 CE), Caucasus-related ancestry penetrated the Near East. Thus, in the past 4,000 years, three limited admixture events detectably impacted the population, complementing the historical records of this culturally complex region dominated by the elite with genetic insights from the general population.

The ancient Near East has been at the center of interaction between the ancient world's civilizations and was ruled at different times by the Egyptians, Hittites, Assyrians, Babylonians, Persians, Greeks, Romans, Arabs, Crusaders, Mamluks, and Ottomans, most of whom left a permanent cultural impact on the local population. However, their genetic contribution is not as evident: our previous ancient DNA (aDNA) work showed that people who live in the Near East today derive ~90% of their ancestry from the local Bronze Age population that preceded all of the aforementioned historical conquests.[1] These results might appear to challenge the historical records of population movements, colonization, and admixture with the locals throughout history. For example, in 1307 CE, the Mamluks divided Lebanon's coast among 300 newly introduced Turkoman families, and a few centuries earlier the Romans had declared Beirut and Baalbek in Lebanon as colonies and garrison towns;[2] additionally the names of Hellenistic army soldiers and their descendants in Lebanon can still be read today from inscriptions on funerary stela found in Sidon.[3] Similarly, our analysis of aDNA from a Crusader burial site in Lebanon showed that immigration to the Near East and admixture with the locals was common, and for a period, a heterogeneous population of Europeans, locals, and their admixed descendants lived in the Near East.[4] However, this admixture appears to not have been widespread enough to leave a permanent genetic impact on the local population, and subsequent mixing with people carrying the local ancestry "diluted" the ancestry of the Crusaders in Near Eastern genomes to undetectable levels. The example of the Crusaders might illustrate why, even after numerous conquests and immigrations, the Near Eastern Bronze Age ancestry still dominates present-day Near Eastern genomes. Thus, two outstanding questions emerge from the previous aDNA studies: (1) were transient admixture events a common occurrence in the history of the Near East, or was the Crusaders period an exception, and (2) because present-day Near Easterners derive most but not all of their ancestry from the local Bronze Age population, which post-Bronze Age events contributed to the genetic diversity we observe today in the Near East.

To address these questions, we have now sequenced the genomes of ancient individuals who lived between 800 BCE and 200 CE at one of four different time periods: the Iron Age II (1000–539 BCE), the Iron Age III (539–330 BCE), the Hellenistic period (330–31 BCE), and the early Roman period (31 BCE–200 CE) (Table 1). These data, together with previous data we generated from individuals from the same region from the Middle Bronze Age (2100–1550 BCE), the late Roman period (200–634 CE), the Crusader period (1099–1291 CE), and the present-day

[1]Institute of Cancer and Genomic Sciences, University of Birmingham, Birmingham B15 2TT, UK; [2]Centre for Computational Biology, University of Birmingham, Birmingham B15 2TT, UK; [3]Wellcome Sanger Institute, Wellcome Genome Campus, Hinxton CB10 1SA, UK; [4]Institut Français du Proche-Orient, BP 11-1424, Beirut, Lebanon; [5]Institute of Genomics, University of Tartu, Riia 23b, 51010 Tartu, Estonia; [6]Department of Evolutionary Biology, Institute of Cell and Molecular Biology, University of Tartu, Tartu 51010, Estonia; [7]The Sidon Excavation, Saida, Lebanon; [8]Département des Antiquités Orientales, Musée du Louvre, France; [9]Université Libanaise, Rectorat, BP 14-6573, Place du Musée, Beirut, Lebanon
*Correspondence: m.haber@bham.ac.uk (M.H.), cts@sanger.ac.uk (C.T.-S.)
https://doi.org/10.1016/j.ajhg.2020.05.008.
© 2020 The Author(s). This is an open access article under the CC BY license (http://creativecommons.org/licenses/by/4.0/).

Table 1. **Samples Analyzed in this Study**

ENA Number	ID	Excavation Site	Period	Date (Calibrated)	Mapped Read %	Coverage Genomic	SNPs Overlapping with Published aDNA Data
ERS4542976	SFI-56	Beirut SFI-415	Iron Age II	–	15	0.7	568,628
ERS4542991	SFI-55	Beirut SFI-415	Iron Age II	–	8	0.4	408,015
ERS4542962	SFI-43	Beirut SFI-1075	Iron Age III	567 BCE–404 BCE	17	0.5	428,888
ERS4542967	SFI-50	Beirut SFI-1075	Iron Age III	–	31	1	703,041
ERS4542969	SFI-36	Beirut SFI-1075	Iron Age III	–	19	0.8	590,514
ERS4542989	SFI-42	Beirut SFI-1075	Iron Age III	540 BCE–396 BCE	13	0.5	440,585
ERS4542964	SFI-45	Beirut SFI-1075	Iron Age III	–	24	0.6	478,277
ERS4542984	SFI-34	Beirut SFI-1075	Iron Age III	–	27	1.7	933,032
ERS4542983	SFI-35	Beirut SFI-1075	Iron Age III	–	5	0.3	321,527
ERS4542988	SFI-39	Beirut SFI-1075	Iron Age III	–	13	0.7	567,178
ERS4542990	SFI-44	Beirut SFI-1075	Iron Age III	–	41	1.6	889,705
ERS4542987	SFI-47	Beirut SFI-1075	Iron Age III	–	23	1.1	747,390
ERS4542979	SFI-20	Beirut SFI-477	Hellenistic	199 BCE–37 BCE	13	0.8	691,379
ERS4542972	SFI-5	Beirut SFI-477	Hellenistic	234 BCE–92 BCE	3	0.1	140,660
ERS4542974	SFI-12	Beirut SFI-477	Hellenistic	209 BCE–89 BCE	2	0.1	106,051
ERS4542980	SFI-24	Beirut SFI-1106	early Roman	55 BCE–58 CE	39	3.3	1,093,459
ERS4542982	SFI-33	Beirut SFI-1106	early Roman	48 CE–222 CE	43	3.3	1,087,690
ERS4542973	SFI-11	Beirut SFI-477	early Roman	119 BCE–27 CE	2	0.1	132,450
ERS4542977	SFI-15	Beirut SFI-477	early Roman	176 BCE–3 CE	28	1.4	915,901

provide a genetic representation of the Near East in a time series spanning the past 4,000 years (Table S1).

We sampled the petrous portion of the temporal bones from 67 individuals buried in Beirut (Figures S1 and S2), a city on the Eastern Mediterranean coast that has had continuous settlement dating back 5,000 years and that is the capital of modern-day Lebanon. We extracted DNA and built double-stranded libraries according to published protocols[5–7] and sequenced the libraries on Illumina HiSeq 2500 and HiSeq 4000 platforms with 2 × 75 bp reads. We processed the sequences by using PALEOMIX[8] as described previously[4] and mapped the merged sequences to the hs37d5 reference sequence (see Supplemental Methods). We found 19 samples that had 2%–43% endogenous DNA with post-mortem damage patterns typical of ancient DNA (Figure S3), and subsequent sequencing of these libraries resulted in genomic coverage between 0.1× and 3.3× (Table 1). We estimated contamination from the X chromosomes of males and the mtDNA genome of all individuals[9,10] and found that the sequence data were minimally contaminated (Tables S2 and S3).

We combined the new data with published ancient and modern data, creating two datasets: set 1 included 2,012 modern humans[1,11–14] and 914 ancient individuals[6,15–37] with 815,791 SNPs, and set 2 consisted of 2,788 modern humans[24,38,39] and 914 ancient individuals with 539,766 SNPs (see Supplemental Methods).

We then estimated kinship[40] among our samples and found individuals SFI-43 (female) and SFI-44 (male), who lived around 500 BCE during the Iron Age III under the Persian rule, were first-degree relatives (Figure S4) and shared the same mtDNA haplogroup, T2C1 (Table S4). We kept these two individuals in the dataset for the following test and projected all ancient samples in set 2 onto a principal component analysis (PCA)[41] plot based on variation in modern West, Central and South Eurasians (Figures 1 and S5). The plot differentiates between populations from the Near East, Europe, Caucasus, Russian Steppe, Central and South Asia. The ancient Lebanese (i.e., ancient individuals who lived in what is today known as Lebanon) clustered with the modern and ancient Near Easterners: the new samples clustered between the Bronze Age population (Sidon_BA) and modern Lebanese. The two first-degree relatives, SFI-43 and SFI-44, appeared as outliers and did not cluster with their contemporaries, but instead were positioned close to the Bronze Age samples. We wanted to test whether these two individuals had a genetic affinity to a population other than the ancient Lebanese. Thus, using *qpWave*,[42,43] we selected 11 outgroups (see Supplemental Methods) that have different relationships with the populations found in set 1 and tested whether SFI-43 and SFI-44 formed a clade with any of the populations (including the ancient Lebanese) in our dataset. We found that SFI-43 only formed a clade with ancient Egyptians

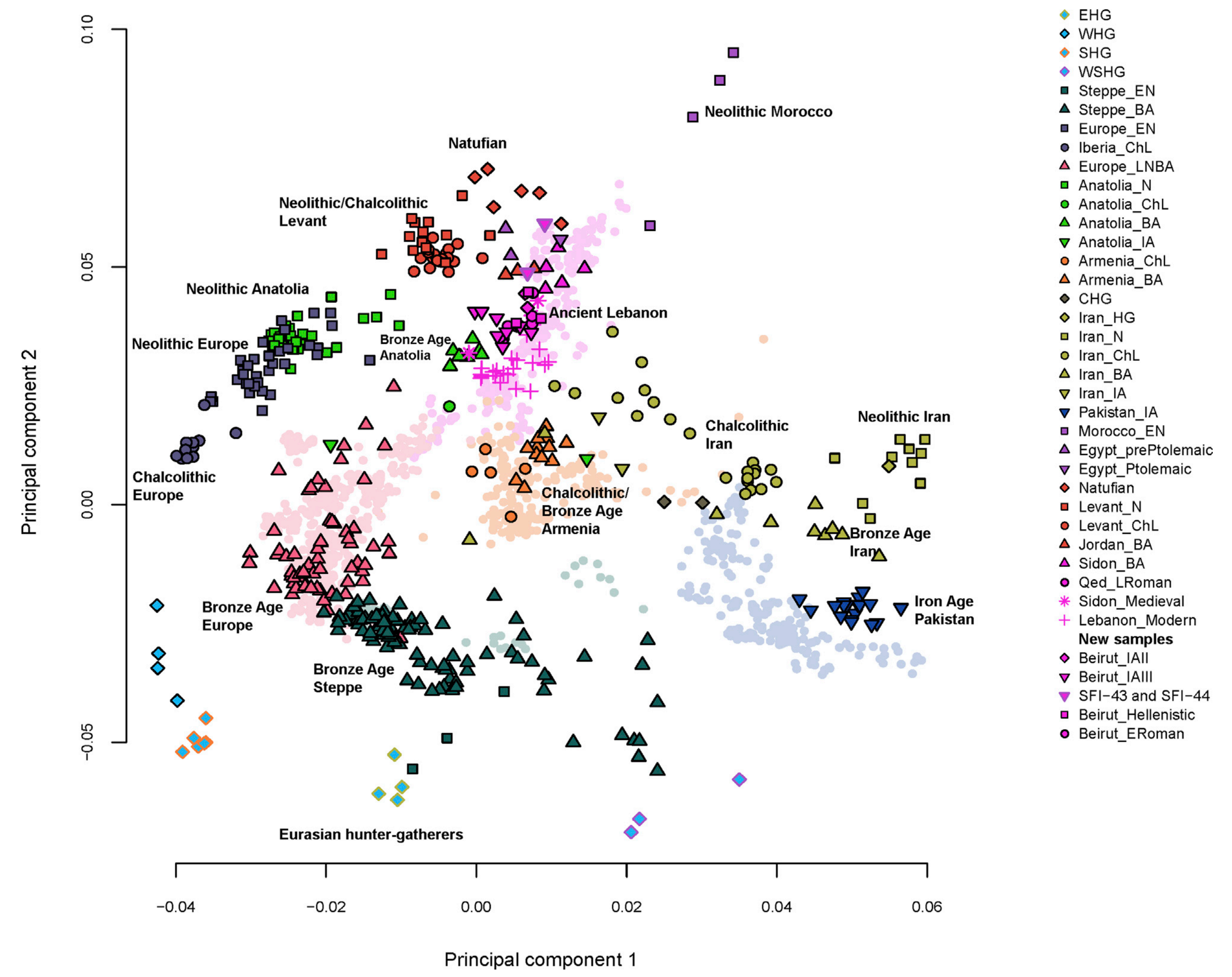

Figure 1. Principal Components Analysis of West, Central, and South Eurasians
Eigenvectors were inferred with present-day populations (light-colored points in the background of the plot), and the ancient samples (colored solid shapes in the foreground of the plot) were projected onto the plot.

(Table S5), implying that she shared all of her ancestry with them or a genetically equivalent population. On the other hand, SFI-44's ancestry appeared to be more complex because he did not form a clade with any population in our dataset, yet he appeared to share ancestry with SFI-43, ancient Egyptians, and ancient Levantines (Table S5). To better understand the relationship of SFI-43 and SFI-44 with the Lebanese and Egyptians, we projected the ancient Lebanese and ancient Egyptians onto a PCA constructed with the variation found in their modern populations. SFI-43 and SFI-44 clustered with the ancient Egyptians and were positioned between modern or ancient Lebanese and modern Egyptians, but SFI-44 was positioned closer than SFI-43 to the Lebanese (Figure S6). Because SFI-43 and SFI-44 are first-degree relatives but appear to have differences in their genetic ancestry, we tested whether SFI-44 can be modeled as a mixture of ancestries deriving from SFI-43 and any other individuals or populations in our dataset by using *qpAdm*.[42] We found that SFI-43 could be modeled as deriving ~70% of his ancestry from a population related to SFI-44 and ~30% from a population related to ancient Levantines (Table S6). But these ancestry proportions do not reflect the first-degree relationship that the two individuals shared unless more than one mixture event had occurred in the family, so we created a simulated hybrid genome that represents a first-generation mixture between an ancient Egyptian and an ancient Lebanese and tested whether SFI-44 could be modeled as descending from a mixture between SFI-43 and the hybrid genome. The model showed that SFI-44 derived ~50% of his ancestry from SFI-43 and ~50% from an individual whose ancestry was similar to that of the hybrid genome (Table S6). Thus, these results suggest that SFI-43 was an Egyptian woman and SFI-44 was her son from a man who himself had both Egyptian and Lebanese ancestries. The structure of this family in Lebanon highlights population movements and the heterogeneous society that existed at that time, but additional

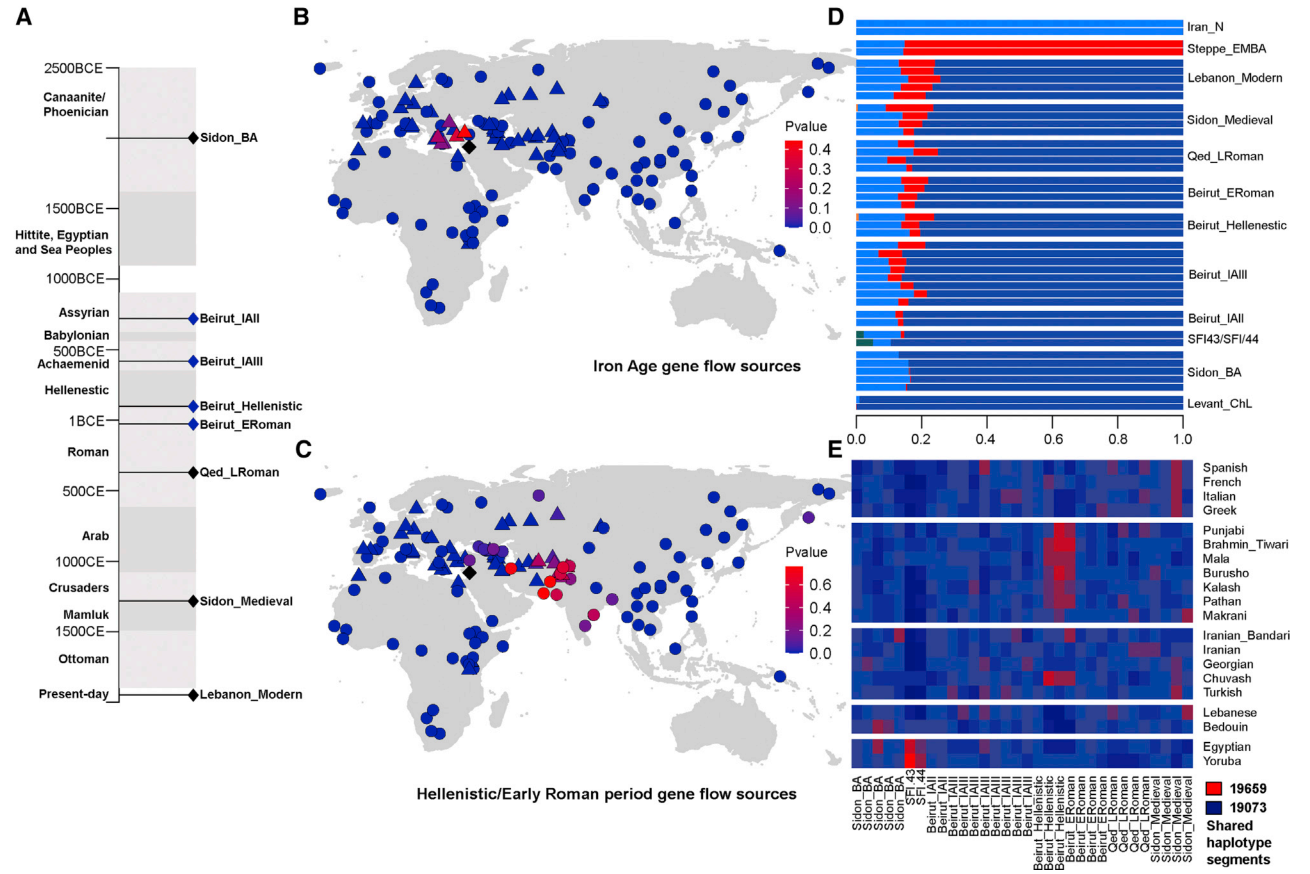

Figure 2. Admixture in Ancient Lebanon

(A) Historical context of the studied samples. Horizontal lines indicate the time period of a sampled population, and the blue lozenges represent newly sequenced samples.

(B and C) Locations of the source populations we used in *qpAdm* to test for admixture at the Iron Age (B) and the Hellenistic/early Roman period (C). The black lozenge on each map shows Lebanon's location. Points represent modern populations in the dataset, whereas triangles represent ancient populations. Increased intensity of the red color indicates a higher p value for the model involving the source population (this should not be interpreted as an indication of the best model). We set the p values of the models that can be rejected to zero.

(D) A DyStruct run with 166,693 transversions found in set 1 across nine time points. We show the plot of $K = 6$, which reveals an ancestral component (red) related to the Bronze Age Steppe population appearing in the Near East after the Bronze Age.

(E) Haplotype segments shared between the ancient Lebanese and global modern populations. The heatmap is based on ChromoPainter's co-ancestry matrix, and we averaged values from the modern populations over all individuals in the population. We scaled the heatmap by row to highlight the differences between the ancient individuals. Two Hellenistic individuals and one early Roman individual showed excess haplotype sharing with Central and South Asian populations compared with that of other ancient Lebanese individuals, whereas individuals SFI-43 and SFI-44 shared more segments with Africans and Egyptians. We counted between 19,073 (blue) and 19,659 (red) shared haplotype chunks in the dataset.

sampling is needed if we are to understand whether this cross-cultural mixing was common or whether our samples were exceptional. We removed SFI-43 and SFI-44 from all following analyses in which local individuals were grouped to represent their respective time periods.

Having genetic representation from eight consecutive time periods (Figure 2A), we were able to test whether two populations that were successive in time formed a clade and derived all of their ancestry from a shared ancestral population or whether subsequent admixture had occurred and the two populations consequently lost their clade relationship. We started by computing *f4*-statistics of the form *f4(Lebanon Period1, Lebanon Period2; Ancient, Chimpanzee)*, in which a result significantly different from zero could indicate that genetic changes related to "Ancient" (an ancient population in our dataset) have occurred between two successive periods in Lebanon. We found that significant genetic changes that were marked by an increase in Eurasian ancestry related to ancient Europeans and ancient Central Asians occurred after the Bronze Age and starting from the Iron Age II (Figure S7A). We did not observe significant genetic differences between the Iron Age II and Iron Age III populations in this test (Figure S7B), and thus, we merged our samples from these two periods into one population (Figure S7C) and used *qpAdm* (see Supplemental Methods) to explore possible Iron Age admixture models (Tables 2 and S7). We found that the Lebanese Iron Age population can be modeled as a mixture of the local Bronze Age

Table 2. Modeling Populations from the Iron Age and Antiquity as a Mixture of the Preceding Population, A, and Any Global Ancient Population, B

				A	B	
Test	A	B	p Value for Rank = 1	Mixture Proportions		Std. Error
Beirut_IA	Sidon_BA	Anatolia_MLBA	4.44×10^{-01}	0.63	0.37	0.06
Beirut_IA	Sidon_BA	Ashkelon_IAI	4.29×10^{-01}	0.69	0.31	0.05
Beirut_IA	Sidon_BA	Anatolia_EBA	3.38×10^{-01}	0.80	0.20	0.03
Beirut_IA	Sidon_BA	Mycenaean	2.17×10^{-01}	0.77	0.23	0.04
Beirut_IA	Sidon_BA	Minoan_Odigitria	1.32×10^{-01}	0.80	0.20	0.04
Beirut_HER	Beirut_IA	Butkara_H	4.93×10^{-01}	0.92	0.08	0.01
Beirut_HER	Beirut_IA	Aligrama2_IA	4.46×10^{-01}	0.93	0.07	0.01
Beirut_HER	Beirut_IA	Indus_Periphery	3.88×10^{-01}	0.93	0.07	0.01
Beirut_HER	Beirut_IA	Swat_H	3.24×10^{-01}	0.92	0.08	0.01
Beirut_HER	Beirut_IA	SPGT_IA	2.65×10^{-01}	0.93	0.07	0.01

We show the top five models for each test based on their p value for the rank = 1 matrix. A p value > 0.05 indicates the model cannot be rejected. We removed infeasible models with negative proportions from the table. Beirut_IA included individuals from the Iron Age II and Iron Age III periods and can be modeled as a mixture of the local Bronze Age population and a population related to ancient Anatolians or ancient South-Eastern Europeans. Beirut_HER included individuals from the Hellenistic and early Roman periods and can be modeled as a mixture of the local population Beirut_IA and an ancient Central and South Asian population.

population (63%–88%) and a population related to ancient Anatolians or ancient South-Eastern Europeans (12%–37%) (Table 2 and Figure 2B). We replicated these results by running DyStruct[44] with 166,693 transversions present in set 1 and showed that a Steppe-like ancestry, typically found in Europeans, appears in the Near East starting from the Iron Age II (Figure 2D). A potential source of this exogenous ancestry could be the Sea Peoples, a seafaring group of people with a disputed origin who attacked the Eastern Mediterranean and Egypt after the Bronze Age (1200–900 BCE). One of our successful models for admixture involved an ancestry source related to the Ashkelon (a city situated ~170 miles south of the Beirut sites) Iron Age I population, which was previously identified as possibly descending from Sea-Peoples-related admixture.[18] In addition, according to ancient Egyptian texts and archaeology, the Sea Peoples conquered the Levant but failed to conquer the Egyptians. Therefore, we tested whether the Eurasian gene flow to Lebanon during the Iron Age had also reached ancient Egypt by quantifying the Steppe ancestry in both regions at that time and found *f4(Sidon_BA, Beirut_IAII; Steppe_EMBA, Chimp)* is significantly negative (*Z* score = −4.13), but *f4(Sidon_BA, Egypt_prePtolemaic; Steppe_EMBA, Chimp)* has a value not significantly different from zero (*Z* score = 0.317), suggesting that either ancient Egypt did not receive the Eurasian gene flow that the Levant received during the Iron Age or that the Eurasian ancestry was replaced in Egypt as in Ashkelon, where in contrast to the Beirut_IAII, the European-related ancestry was no longer significant in the Ashkelon Iron Age II population.[18] Additional Iron Age samples from the Levant coast and Egypt could reveal whether the Iron Age admixture had a north to south cline as a result of the location of the source populations or from differences in the scale of the successful migrations to the north or south of the Levant during this period.

The second genetic change in ancient Lebanon can be observed during the Hellenistic and early Roman periods. We merged individuals from these two periods into one population (Beirut_HER) because several individuals had overlapping radiocarbon dates and the *f4*-statistics showed symmetry between the Beirut_Hellenistic and Beirut_ERoman populations (Figure S8). We found that the Hellenistic and early Roman population can be modeled as a mixture of the local population, Beirut_IA (88%–94%), and a Central/South Asian population (6%–12%) (Tables 2 and S8 and Figure 2C). We then analyzed haplotype segments shared between the ancient Lebanese and modern populations in set 2 by using ChromoPainter[44] on 2.5 million imputed SNPs and found that two Hellenistic individuals (SFI-5 and SFI-12) and one early Roman individual (SFI-11) had excess haplotype sharing with Central and South Asians (Figures 2E and S9), thus confirming the *qpAdm* results. The relationship of ancient Lebanon with Central and South Asia also manifests in the presence of haplogroup L1a1-M27 among the modern Lebanese Y chromosome lineages (Figure S10). Haplogroup L1a1-M27 is common today in Central and South Asia but rare elsewhere (in the 1000 Genomes Project,[45] this lineage was found exclusively in Sri Lankan Tamil from the UK [STU], Punjabi from Lahore, Pakistan [PJL], Indian Telugu from the UK [ITU], Gujarati Indian from Houston, Texas [GIH], and Bengali from Bangladesh [BEB]). We tested[46] (see Supplemental Methods) the coalescence of the five L1a1-M27 Lebanese chromosomes and found that they all derived from a man who lived around 450 BCE–50 CE, a time interval overlapping with the Hellenistic period (Figure S10). The presence of the Central/South Asian ancestry in

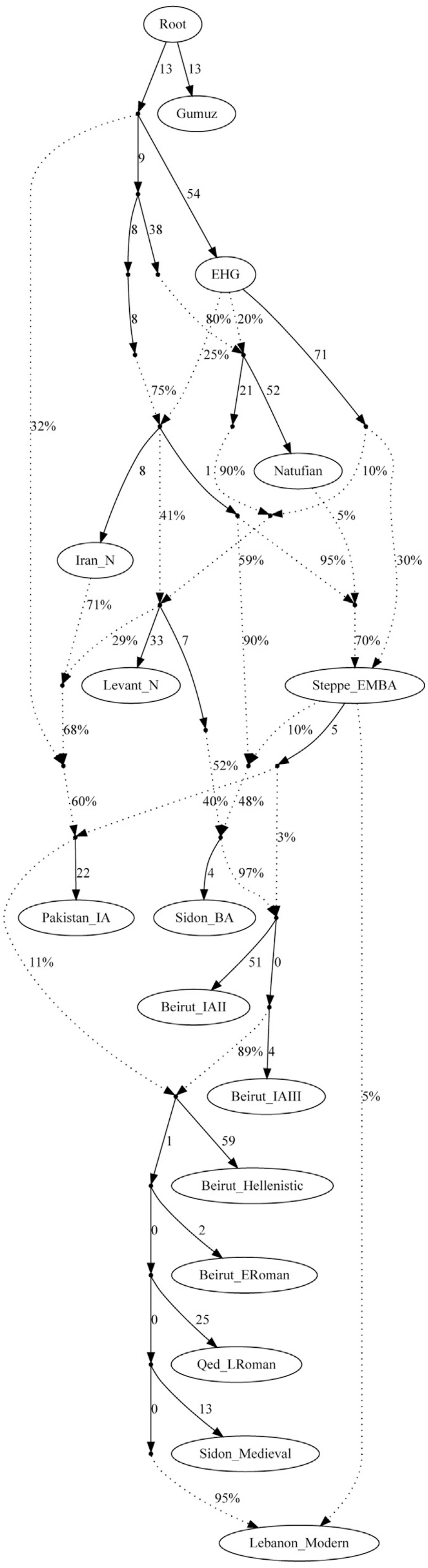

Figure 3. An Admixture Graph Model for Ancient Lebanon
A graph model that fits our data showing the relationship between the ancient Lebanon populations and the admixture events that contributed to the population until modern times. Worst *f4*-statistics, Iran_N,Levant_N;EHG,Qed_LRoman; *Z* score = 3.0. See Figure S13 for alternative graph models.

Lebanon during the Hellenistic period mirrors the connected geography under the rule of Alexander the Great's empire, which had also assimilated the Achaemenid Empire that preceded it and thus maintained a connection between the West and East for five centuries. These large contiguous empires thus facilitated the movement and mixture of people as seen directly by the Egyptian-Lebanese family and the admixed individuals reported here who lived in the Near East at that time.

We next tested the genetic changes between the Hellenistic/early Roman period and the late Roman period (Qed_LRoman) and found little genetic differences from the *f4*-statistics (Figure S11), which is notable because during this period there was significant population movement between the Near East and Europe, as identified from the genomes of ancient Near Easterners found in Rome at that time.[16] When we model Qed_LRoman as a mixture of the Hellenestic/early Roman period population and another ancient population, we find successful models involving ancient Anatolians and South-Eastern Europeans (Table S9). However, because this ancestry was already present in Lebanon starting from the Iron Age, its excess in Qed_LRoman could be from population structure, especially because the Qed_LRoman samples were from a remote mountainous region, whereas the Hellenistic/early Roman samples were from the coast, and in addition, we found that the admixture models were not significant when Beirut_IA was used as the source of the local ancestry, showing that Qed_LRoman derived all of its ancestry from preceding local populations (Table S9).

From the late Roman period to the medieval period, we detect an increase in African ancestry (Figure S11B), but that increase remains slightly below statistical significance (Z score = −2.4) and accounts for ~2.9% of Lebanon_Medieval's ancestry when ancient East Africans are used in the admixture model (Table S10). The final genetic change observed in Lebanon occurred after the Crusaders' period but, as we showed previously,[4] was not related to the Crusaders themselves. We found[4] an increase in ancestry related to populations from the Caucasus and Turks in the modern Lebanese population after the medieval period (Figure S11C and Table S11). Using admixture-induced linkage disequilibrium (LD) decay,[47,48] we show that admixture occurred around 1640–1740 CE when Lebanon was under Ottoman rule (Figure S12). The LD-decay test also detects significant admixture that occurred during the Hellenistic period, which is consistent with our more direct inferences from the ancient individuals analyzed here (Figure S12).

Finally, we fit all the ancient and modern Lebanese data into an admixture graph model showing their relationship with other ancient populations by using data in set 2. The graph supports the results reported here, showing substantial genetic continuity in Lebanon since the Bronze Age interrupted by three significant admixture events during the Iron Age, Hellenistic period, and Ottoman period, each contributing 3%–11% of non-local ancestry to the admixed population (Figures 3 and S13).

In this study, we present new whole-genome sequence data from ancient individuals who lived in the Near East between the Iron Age and the Roman period, spanning a time marked by major historical events and population movements. Our data capture the genetic outcome of some of these events but also show that the genetic composition of the general population was minimally affected and that great cultural transitions in the Near East were not in these cases matched by comparable genetic transitions. Yet, we show that the small genetic changes we detect when using ancient populations sampled from a time series have the power to provide information about past events with details that complement the available historical records.

Data and Code Availability

Raw sequencing reads for the ancient individuals are available through the European Nucleotide Archive (ENA) under accession number ENA: ERP121575. Aligned sequences, genotypes, and imputed genotypes can be obtained from the corresponding author M.H.

Supplemental Data

Supplemental Data can be found online at https://doi.org/10.1016/j.ajhg.2020.05.008.

Acknowledgments

We thank the Directorate General of Antiquities (DGA) in Lebanon for approving (reference: 3333) transfer and processing of bones from Lebanon. We thank Rui Martiniano for discussions related to the Y chromosome analysis. M.H., Y.X., and C.T.-S. were supported by The Wellcome Trust (098051). M.A.A. was supported by the government of Dubai (Dubai Police GHQ). The ancient DNA lab at the University of Tartu is supported by the European Union through the European Regional Development Fund (2014–2020.4.01.16–0030) (C.L.S., T.S., and S.J.G.) and the Estonian Research Council (PRG243) (C.L.S. and L.S.).

Declaration of Interests

The authors declare no competing interests.

Received: February 14, 2020
Accepted: May 11, 2020
Published: May 28, 2020

Web Resources

European Nucleotide Archive (ENA), https://www.ebi.ac.uk/ena
International Society of Genetic Genealogy (ISOGG), https://isogg.org

References

1. Haber, M., Doumet-Serhal, C., Scheib, C., Xue, Y., Danecek, P., Mezzavilla, M., Youhanna, S., Martiniano, R., Prado-Martinez, J., Szpak, M., et al. (2017). Continuity and admixture in the last five millennia of Levantine history from ancient Canaanite and present-day Lebanese genome sequences. Am. J. Hum. Genet. *101*, 274–282.
2. Hitti, P.K. (1965). A short history of Lebanon (London: Macmillan).
3. Launey, M. (1949). Recherches sur les armées hellénistiques (Paris: E. de Boccard).
4. Haber, M., Doumet-Serhal, C., Scheib, C.L., Xue, Y., Mikulski, R., Martiniano, R., Fischer-Genz, B., Schutkowski, H., Kivisild, T., and Tyler-Smith, C. (2019). A transient pulse of genetic admixture from the crusaders in the Near East identified from ancient genome sequences. Am. J. Hum. Genet. *104*, 977–984.
5. Dabney, J., Knapp, M., Glocke, I., Gansauge, M.T., Weihmann, A., Nickel, B., Valdiosera, C., García, N., Pääbo, S., Arsuaga, J.L., and Meyer, M. (2013). Complete mitochondrial genome sequence of a Middle Pleistocene cave bear reconstructed from ultrashort DNA fragments. Proc. Natl. Acad. Sci. USA *110*, 15758–15763.
6. Gamba, C., Jones, E.R., Teasdale, M.D., McLaughlin, R.L., Gonzalez-Fortes, G., Mattiangeli, V., Domboróczki, L., Kővári, I., Pap, I., Anders, A., et al. (2014). Genome flux and stasis in a five millennium transect of European prehistory. Nat. Commun. *5*, 5257.
7. Meyer, M., and Kircher, M. (2010). Illumina sequencing library preparation for highly multiplexed target capture and sequencing. Cold Spring Harb. Protoc. *2010*, t5448.
8. Schubert, M., Ermini, L., Der Sarkissian, C., Jónsson, H., Ginolhac, A., Schaefer, R., Martin, M.D., Fernández, R., Kircher, M., McCue, M., et al. (2014). Characterization of ancient and modern genomes by SNP detection and phylogenomic and metagenomic analysis using PALEOMIX. Nat. Protoc. *9*, 1056–1082.
9. Korneliussen, T.S., Albrechtsen, A., and Nielsen, R. (2014). ANGSD: analysis of next generation sequencing data. BMC Bioinformatics *15*, 356.
10. Rasmussen, M., Guo, X., Wang, Y., Lohmueller, K.E., Rasmussen, S., Albrechtsen, A., Skotte, L., Lindgreen, S., Metspalu, M., Jombart, T., et al. (2011). An Aboriginal Australian genome reveals separate human dispersals into Asia. Science *334*, 94–98.
11. Mallick, S., Li, H., Lipson, M., Mathieson, I., Gymrek, M., Racimo, F., Zhao, M., Chennagiri, N., Nordenfelt, S., Tandon, A., et al. (2016). The Simons Genome Diversity Project: 300 genomes from 142 diverse populations. Nature *538*, 201–206.
12. Mondal, M., Casals, F., Xu, T., Dall'Olio, G.M., Pybus, M., Netea, M.G., Comas, D., Laayouni, H., Li, Q., Majumder, P.P., and Bertranpetit, J. (2016). Genomic analysis of Andamanese provides insights into ancient human migration into Asia and adaptation. Nat. Genet. *48*, 1066–1070.
13. Pagani, L., Schiffels, S., Gurdasani, D., Danecek, P., Scally, A., Chen, Y., Xue, Y., Haber, M., Ekong, R., Oljira, T., et al. (2015). Tracing the route of modern humans out of Africa by using 225 human genome sequences from Ethiopians and Egyptians. Am. J. Hum. Genet. *96*, 986–991.
14. Auton, A., Brooks, L.D., Durbin, R.M., Garrison, E.P., Kang, H.M., Korbel, J.O., Marchini, J.L., McCarthy, S., McVean, G.A., Abecasis, G.R.; and 1000 Genomes Project Consortium (2015). A global reference for human genetic variation. Nature *526*, 68–74.
15. Allentoft, M.E., Sikora, M., Sjögren, K.G., Rasmussen, S., Rasmussen, M., Stenderup, J., Damgaard, P.B., Schroeder, H., Ahlström, T., Vinner, L., et al. (2015). Population genomics of Bronze Age Eurasia. Nature *522*, 167–172.

16. Antonio, M.L., Gao, Z., Moots, H.M., Lucci, M., Candilio, F., Sawyer, S., Oberreiter, V., Calderon, D., Devitofranceschi, K., Aikens, R.C., et al. (2019). Ancient Rome: A genetic crossroads of Europe and the Mediterranean. Science *366*, 708–714.
17. de Barros Damgaard, P., Martiniano, R., Kamm, J., Moreno-Mayar, J.V., Kroonen, G., Peyrot, M., Barjamovic, G., Rasmussen, S., Zacho, C., Baimukhanov, N., et al. (2018). The first horse herders and the impact of early Bronze Age steppe expansions into Asia. Science *360*, eaar7711.
18. Feldman, M., Master, D.M., Bianco, R.A., Burri, M., Stockhammer, P.W., Mittnik, A., Aja, A.J., Jeong, C., and Krause, J. (2019). Ancient DNA sheds light on the genetic origins of early Iron Age Philistines. Sci. Adv. *5*, eaax0061.
19. Fregel, R., Méndez, F.L., Bokbot, Y., Martín-Socas, D., Camalich-Massieu, M.D., Santana, J., Morales, J., Ávila-Arcos, M.C., Underhill, P.A., Shapiro, B., et al. (2018). Ancient genomes from North Africa evidence prehistoric migrations to the Maghreb from both the Levant and Europe. Proc. Natl. Acad. Sci. USA *115*, 6774–6779.
20. Fu, Q., Li, H., Moorjani, P., Jay, F., Slepchenko, S.M., Bondarev, A.A., Johnson, P.L., Aximu-Petri, A., Prüfer, K., de Filippo, C., et al. (2014). Genome sequence of a 45,000-year-old modern human from western Siberia. Nature *514*, 445–449.
21. Harney, É., May, H., Shalem, D., Rohland, N., Mallick, S., Lazaridis, I., Sarig, R., Stewardson, K., Nordenfelt, S., Patterson, N., et al. (2018). Ancient DNA from Chalcolithic Israel reveals the role of population mixture in cultural transformation. Nat. Commun. *9*, 3336.
22. Jones, E.R., Gonzalez-Fortes, G., Connell, S., Siska, V., Eriksson, A., Martiniano, R., McLaughlin, R.L., Gallego Llorente, M., Cassidy, L.M., Gamba, C., et al. (2015). Upper Palaeolithic genomes reveal deep roots of modern Eurasians. Nat. Commun. *6*, 8912.
23. Lazaridis, I., Mittnik, A., Patterson, N., Mallick, S., Rohland, N., Pfrengle, S., Furtwängler, A., Peltzer, A., Posth, C., Vasilakis, A., et al. (2017). Genetic origins of the Minoans and Mycenaeans. Nature *548*, 214–218.
24. Lazaridis, I., Nadel, D., Rollefson, G., Merrett, D.C., Rohland, N., Mallick, S., Fernandes, D., Novak, M., Gamarra, B., Sirak, K., et al. (2016). Genomic insights into the origin of farming in the ancient Near East. Nature *536*, 419–424.
25. Lipson, M., Szécsényi-Nagy, A., Mallick, S., Pósa, A., Stégmár, B., Keerl, V., Rohland, N., Stewardson, K., Ferry, M., Michel, M., et al. (2017). Parallel palaeogenomic transects reveal complex genetic history of early European farmers. Nature *551*, 368–372.
26. Gallego Llorente, M., Jones, E.R., Eriksson, A., Siska, V., Arthur, K.W., Arthur, J.W., Curtis, M.C., Stock, J.T., Coltorti, M., Pieruccini, P., et al. (2015). Ancient Ethiopian genome reveals extensive Eurasian admixture throughout the African continent. Science *350*, 820–822.
27. Mathieson, I., Lazaridis, I., Rohland, N., Mallick, S., Patterson, N., Roodenberg, S.A., Harney, E., Stewardson, K., Fernandes, D., Novak, M., et al. (2015). Genome-wide patterns of selection in 230 ancient Eurasians. Nature *528*, 499–503.
28. Mathieson, I., Alpaslan-Roodenberg, S., Posth, C., Szécsényi-Nagy, A., Rohland, N., Mallick, S., Olalde, I., Broomandkhoshbacht, N., Candilio, F., Cheronet, O., et al. (2018). The genomic history of southeastern Europe. Nature *555*, 197–203.
29. Narasimhan, V.M., Patterson, N., Moorjani, P., Rohland, N., Bernardos, R., Mallick, S., Lazaridis, I., Nakatsuka, N., Olalde, I., Lipson, M., et al. (2019). The formation of human populations in South and Central Asia. Science *365*, eaat7487.
30. Olalde, I., Brace, S., Allentoft, M.E., Armit, I., Kristiansen, K., Booth, T., Rohland, N., Mallick, S., Szécsényi-Nagy, A., Mittnik, A., et al. (2018). The Beaker phenomenon and the genomic transformation of northwest Europe. Nature *555*, 190–196.
31. Olalde, I., Mallick, S., Patterson, N., Rohland, N., Villalba-Mouco, V., Silva, M., Dulias, K., Edwards, C.J., Gandini, F., Pala, M., et al. (2019). The genomic history of the Iberian Peninsula over the past 8000 years. Science *363*, 1230–1234.
32. Prendergast, M.E., Lipson, M., Sawchuk, E.A., Olalde, I., Ogola, C.A., Rohland, N., Sirak, K.A., Adamski, N., Bernardos, R., Broomandkhoshbacht, N., et al. (2019). Ancient DNA reveals a multistep spread of the first herders into sub-Saharan Africa. Science *365*, eaaw6275.
33. van de Loosdrecht, M., Bouzouggar, A., Humphrey, L., Posth, C., Barton, N., Aximu-Petri, A., Nickel, B., Nagel, S., Talbi, E.H., El Hajraoui, M.A., et al. (2018). Pleistocene North African genomes link Near Eastern and sub-Saharan African human populations. Science *360*, 548–552.
34. Schuenemann, V.J., Peltzer, A., Welte, B., van Pelt, W.P., Molak, M., Wang, C.C., Furtwängler, A., Urban, C., Reiter, E., Nieselt, K., et al. (2017). Ancient Egyptian mummy genomes suggest an increase of Sub-Saharan African ancestry in post-Roman periods. Nat. Commun. *8*, 15694.
35. Villalba-Mouco, V., van de Loosdrecht, M.S., Posth, C., Mora, R., Martínez-Moreno, J., Rojo-Guerra, M., Salazar-García, D.C., Royo-Guillén, J.I., Kunst, M., Rougier, H., et al. (2019). Survival of Late Pleistocene Hunter-Gatherer Ancestry in the Iberian Peninsula. Curr. Biol. *29*, 1169–1177.e7.
36. Mittnik, A., Wang, C.C., Pfrengle, S., Daubaras, M., Zariņa, G., Hallgren, F., Allmäe, R., Khartanovich, V., Moiseyev, V., Tõrv, M., et al. (2018). The genetic prehistory of the Baltic Sea region. Nat. Commun. *9*, 442.
37. Günther, T., Valdiosera, C., Malmström, H., Ureña, I., Rodriguez-Varela, R., Sverrisdóttir, O.O., Daskalaki, E.A., Skoglund, P., Naidoo, T., Svensson, E.M., et al. (2015). Ancient genomes link early farmers from Atapuerca in Spain to modern-day Basques. Proc. Natl. Acad. Sci. USA *112*, 11917–11922.
38. Patterson, N., Moorjani, P., Luo, Y., Mallick, S., Rohland, N., Zhan, Y., Genschoreck, T., Webster, T., and Reich, D. (2012). Ancient admixture in human history. Genetics *192*, 1065–1093.
39. Lazaridis, I., Patterson, N., Mittnik, A., Renaud, G., Mallick, S., Kirsanow, K., Sudmant, P.H., Schraiber, J.G., Castellano, S., Lipson, M., et al. (2014). Ancient human genomes suggest three ancestral populations for present-day Europeans. Nature *513*, 409–413.
40. Monroy Kuhn, J.M., Jakobsson, M., and Günther, T. (2018). Estimating genetic kin relationships in prehistoric populations. PLoS ONE *13*, e0195491.
41. Patterson, N., Price, A.L., and Reich, D. (2006). Population structure and eigenanalysis. PLoS Genet. *2*, e190.
42. Haak, W., Lazaridis, I., Patterson, N., Rohland, N., Mallick, S., Llamas, B., Brandt, G., Nordenfelt, S., Harney, E., Stewardson, K., et al. (2015). Massive migration from the steppe was a source for Indo-European languages in Europe. Nature *522*, 207–211.
43. Reich, D., Patterson, N., Campbell, D., Tandon, A., Mazieres, S., Ray, N., Parra, M.V., Rojas, W., Duque, C., Mesa, N., et al. (2012). Reconstructing Native American population history. Nature *488*, 370–374.
44. Joseph, T.A., and Pe'er, I. (2019). Inference of population structure from time-series genotype data. Am. J. Hum. Genet. *105*, 317–333.

45. Poznik, G.D., Xue, Y., Mendez, F.L., Willems, T.F., Massaia, A., Wilson Sayres, M.A., Ayub, Q., McCarthy, S.A., Narechania, A., Kashin, S., et al.; 1000 Genomes Project Consortium (2016). Punctuated bursts in human male demography inferred from 1,244 worldwide Y-chromosome sequences. Nat. Genet. *48*, 593–599.
46. Haber, M., Jones, A.L., Connell, B.A., Asan, Arciero, E., Yang, H., Thomas, M.G., Xue, Y., and Tyler-Smith, C. (2019). A Rare Deep-Rooting D0 African Y-Chromosomal Haplogroup and Its Implications for the Expansion of Modern Humans Out of Africa. Genetics *212*, 1421–1428.
47. Loh, P.R., Lipson, M., Patterson, N., Moorjani, P., Pickrell, J.K., Reich, D., and Berger, B. (2013). Inferring admixture histories of human populations using linkage disequilibrium. Genetics *193*, 1233–1254.
48. Pickrell, J.K., Patterson, N., Loh, P.R., Lipson, M., Berger, B., Stoneking, M., Pakendorf, B., and Reich, D. (2014). Ancient west Eurasian ancestry in southern and eastern Africa. Proc. Natl. Acad. Sci. USA *111*, 2632–2637.

The American Journal of Human Genetics, Volume *107*

Supplemental Data

A Genetic History of the Near East from an aDNA Time Course Sampling Eight Points in the Past 4,000 Years

Marc Haber, Joyce Nassar, Mohamed A. Almarri, Tina Saupe, Lehti Saag, Samuel J. Griffith, Claude Doumet-Serhal, Julien Chanteau, Muntaha Saghieh-Beydoun, Yali Xue, Christiana L. Scheib, and Chris Tyler-Smith

Supplemental Figures

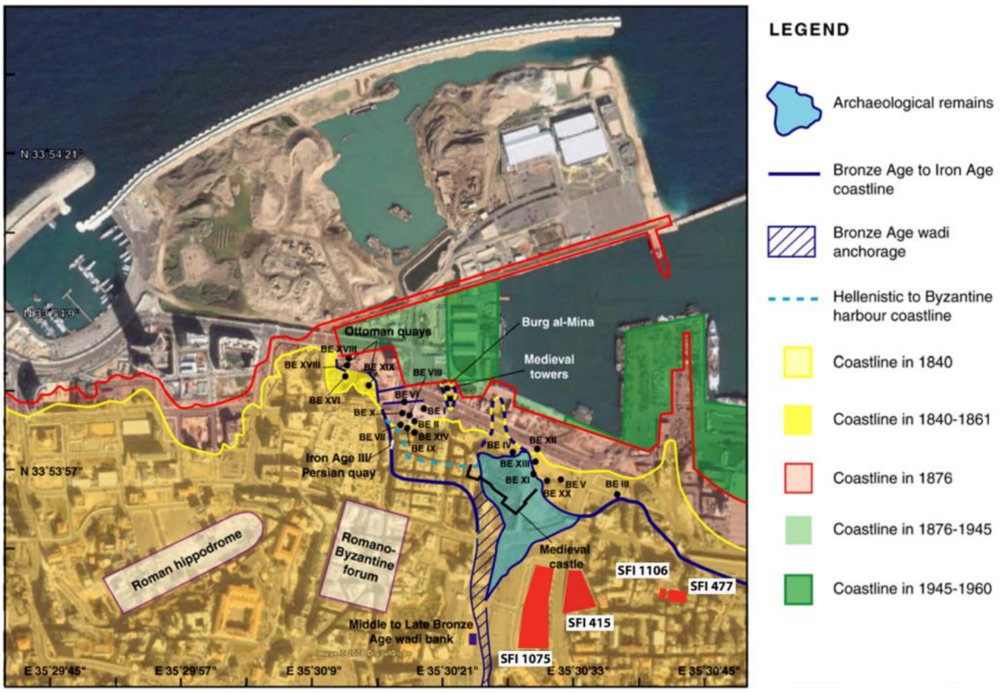

Figure S1. Archaeological parcels location. Saifi 477, 1106, 1075 and 514 are parcels located east of the Central District of modern Beirut which revealed burial sites from the 9th century BCE (Iron Age II) till the 2nd century CE (Roman period). These sites belong to the vast cemeteries of ancient Beirut extending to the south and east and revealing thousands of burials excavated since the 1990's. Iron Age II burials (9th-7th century BCE) excavated in Saifi 415 with samples SFI-55 and SFI-56 extracted from two individuals: one adult (7854) and one subadult (7855) buried in the same burial (T43). Iron Age III burials (6th- 5th BCE) were found partly in Saifi 1075, located few hundred meters to the east of where the settlement was found (west of the Martyrs' square).[1] Ten samples were taken from nine burials: SFI-34 (T5), SFI-35 (T6), SFI-36 (T8), SFI-39 (T12), SFI-42 (T21), SFI-43 (T24), SFI-44 (context 1593), SFI-45 (T25), SFI-47 (T27), SFI-50 (T38). Seven burials ranging from the 2nd BCE till the 2nd CE (Hellenistic-Roman periods) were excavated in two adjacent sites (Saifi 477 and Saifi 1106) 300m east of Saifi 1075. Figure modified from Marriner et al.[2]

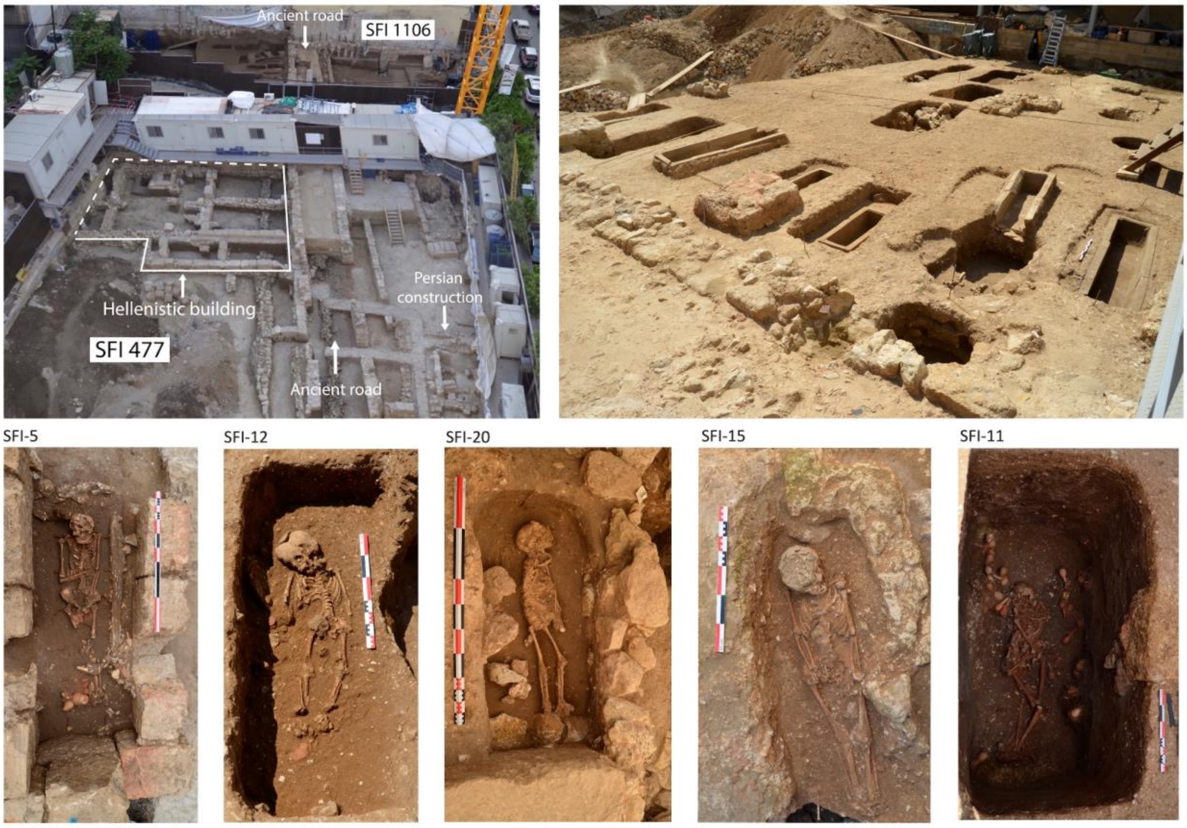

Figure S2. View of parcels Saifi 477 and Saifi 1106. Seven burials ranging from the 2nd BCE till the 2nd CE (Hellenistic-Roman periods) were excavated in two adjacent sites (Saifi 477 and Saifi 1106) 300m east of Saifi 1075. On those sites, the burials were dug along a road used from the Iron Age III (Persian period) till the late Roman period where architectures from the corresponding periods were found. The burials' architecture varies between simple pits dug in soil, simple pits with aligned stones on the edges, or stone built burials. They contain one individual placed on the back and in an extended position, either on a north-south/south-north axis east or on an east-west/west-east axis. In some cases, the deceased is accompanied by funerary material composed of pottery vessels or/and ornamental objects (earrings). The use of wooden containers is evident from conserved remains or iron nails.

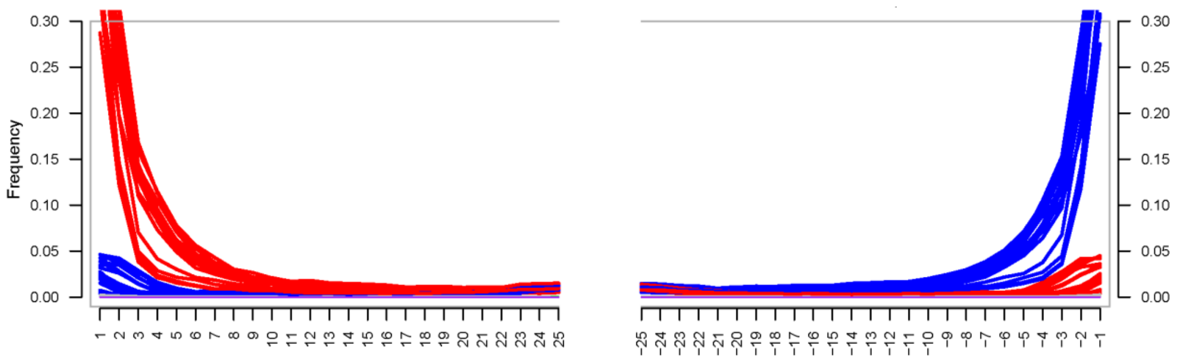

Figure S3. Post-mortem damage patterns.[3] Base substitutions C>T from the 5' (left) and G>A from the 3' end (right) show patterns typical of ancient DNA damage for all samples sequenced in this study.

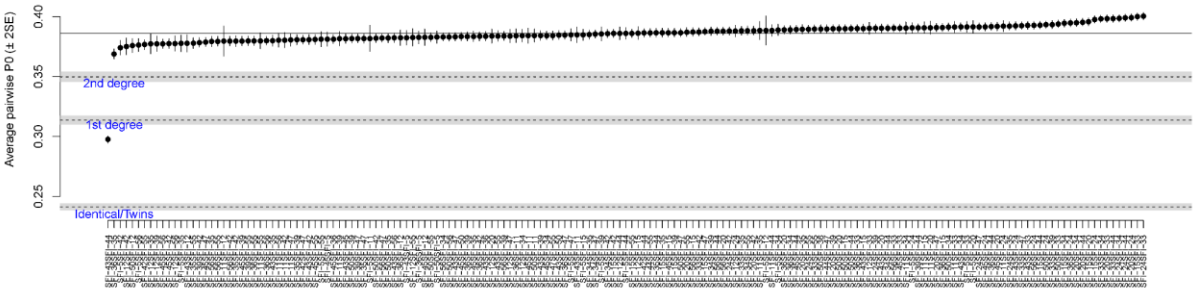

Figure S4. Relationship estimation for the ancient Lebanon samples. We used READ[4] with default parameters on the ancient Lebanon samples in the *Set1* dataset. Two individuals SFI-43 and SFI-44 were identified as first degree relatives.

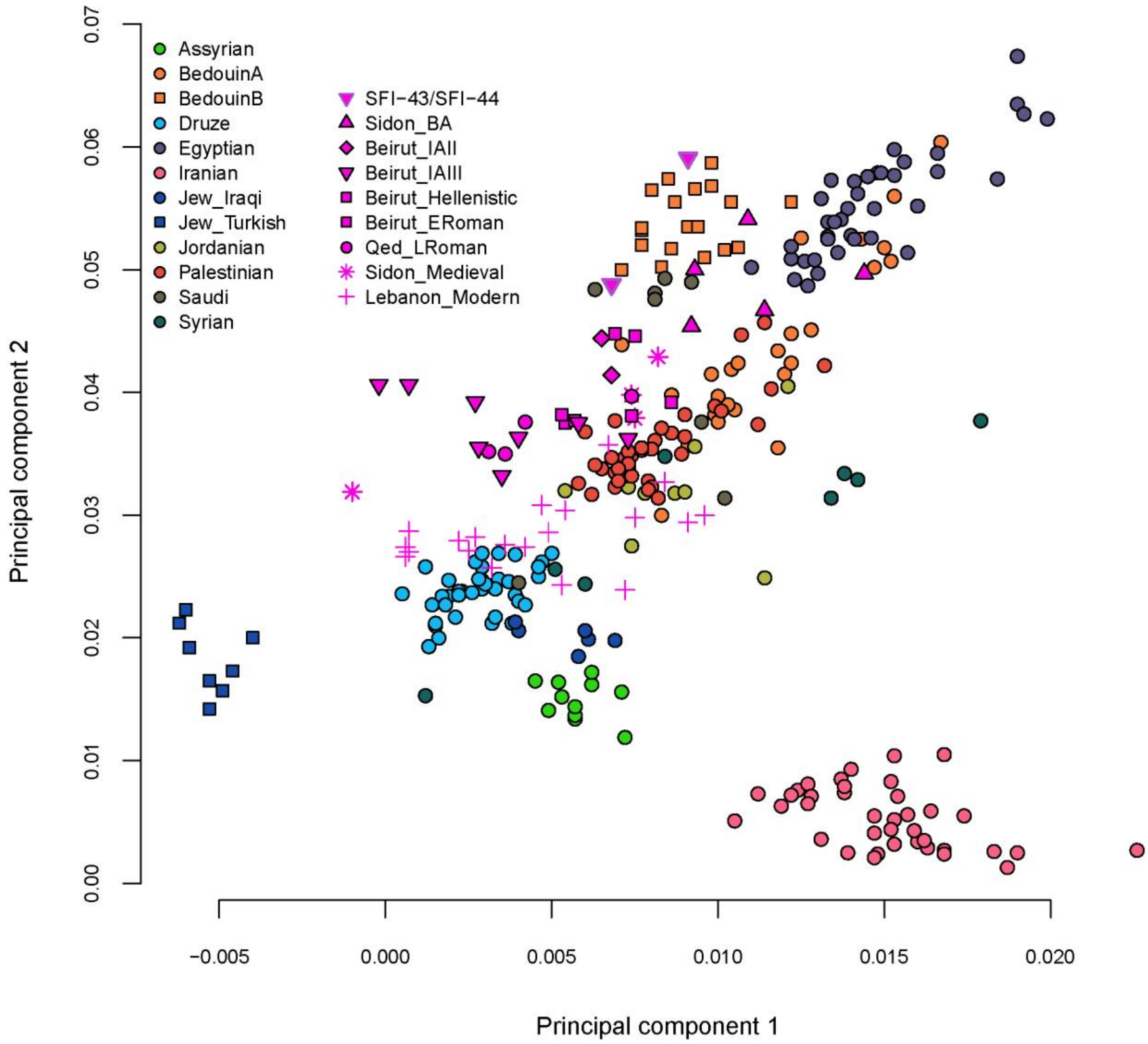

Figure S5. Principal Components Analysis (PCA) showing ancient Lebanon in the context of present-day Near Easterners. PC values for the Near Easterners were extracted from the PCA shown in Figure 1.

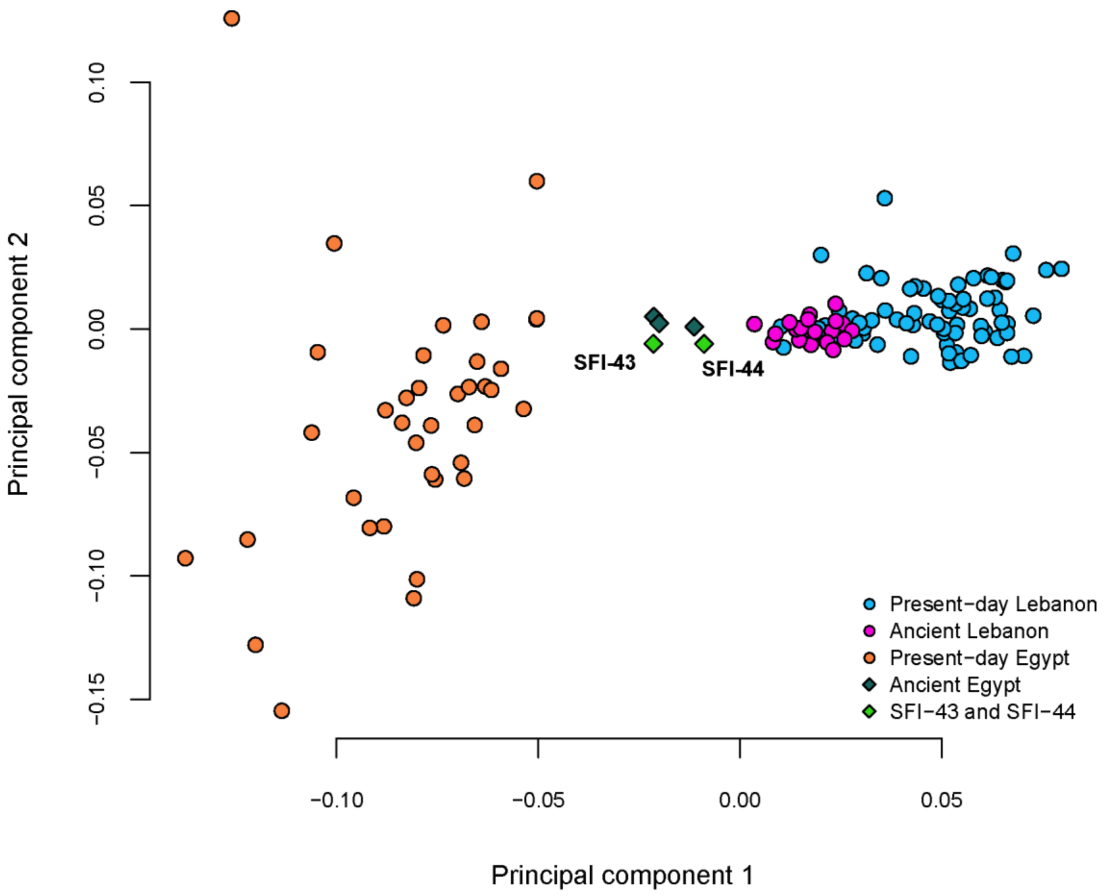

Figure S6. PCA of Egyptians and Lebanese. Individuals SFI-43 and SFI-44 cluster with ancient Egyptians but SFI-44 is closer to ancient individuals from Lebanon.

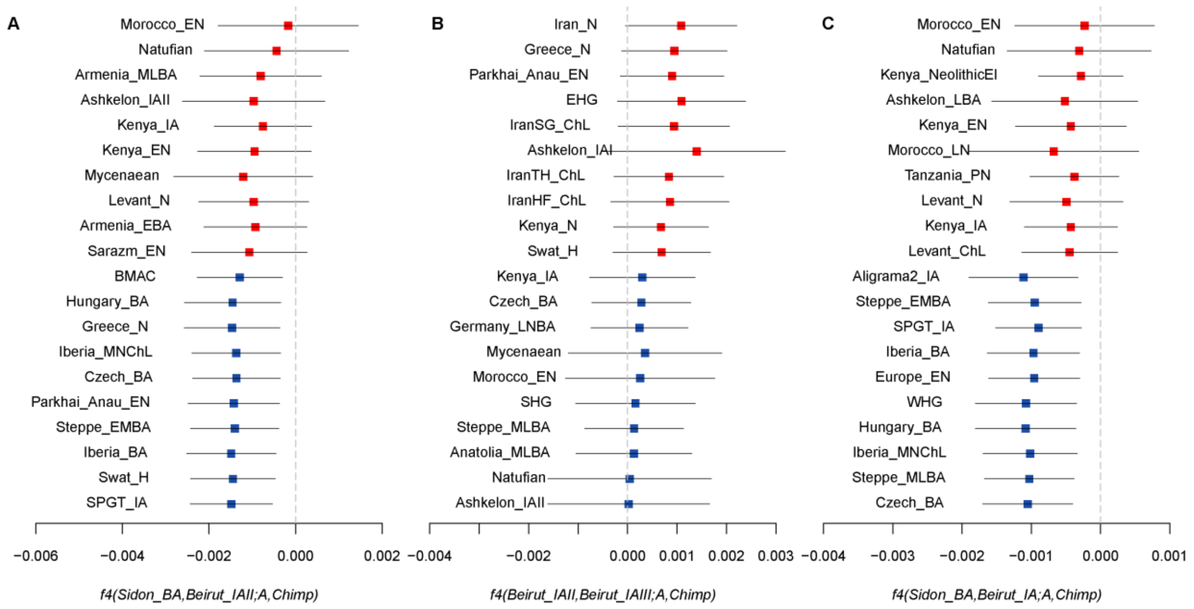

Figure S7. Genetic changes in Lebanon between the Bronze Age and the Iron Age. (A) Comparing the Bronze Age population with the Iron Age II population, (B) the Iron Age II population with the Iron Age III population, and (C) the Bronze Age population with the Iron Age II/Iron Age III populations merged into one group. In this figure and the following we plot the statistic *f4*(Period1,Period2;Ancient,Chimpanzee) and ±3 standard errors from results with the 10 lowest (blue) and 10 highest (red) Z-scores.

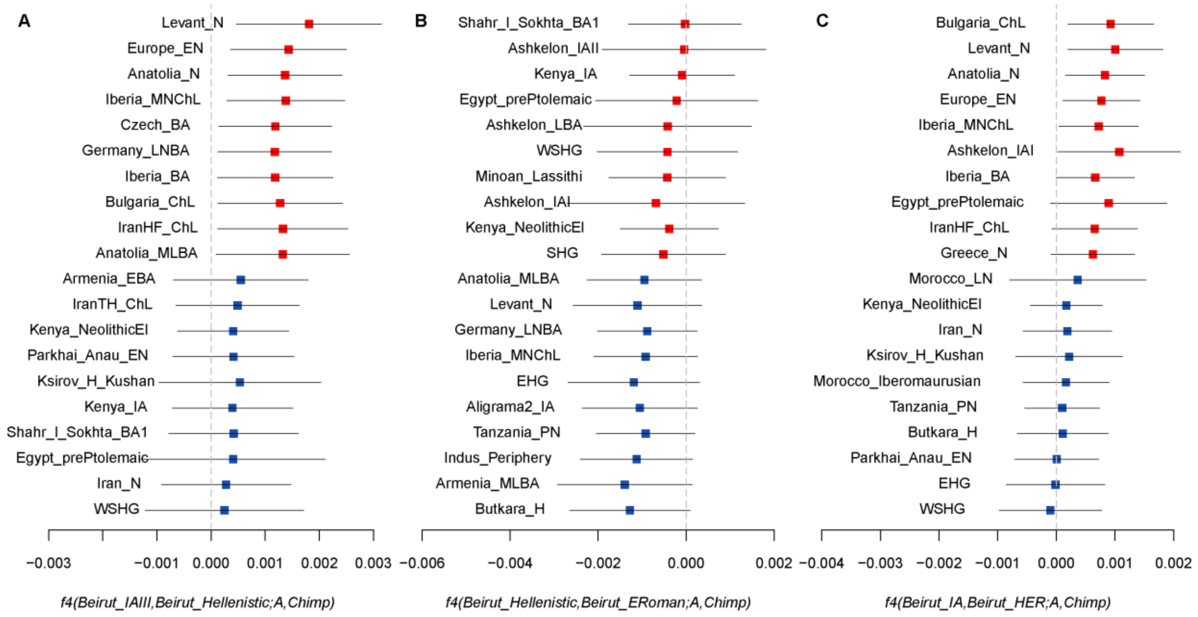

Figure S8. Genetic changes in Lebanon between the Iron Age and the Hellenistic/Early Roman periods. (A) Comparing the Iron Age III population with the Hellenistic period population, (B) the Hellenistic with the Early Roman period, and (C) the Iron Age (merged populations of Beirut_IAII and Beirut_IAIII) with Beirut_HER (merged populations of Beirut_Hellenistic and Beirut_ERoman).

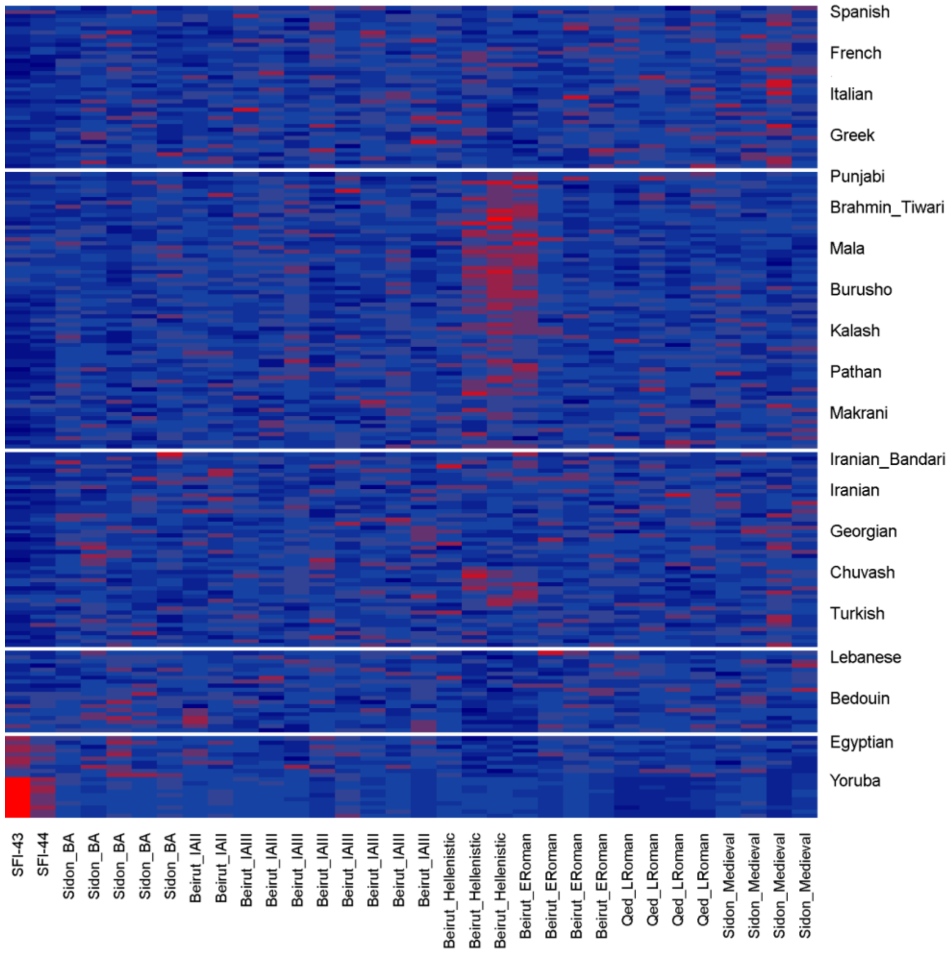

Figure S9. Haplotype sharing between ancient Lebanese and present-day populations. Plot similar to Figure 2E but showing results from the individuals in the reference populations rather than the population average.

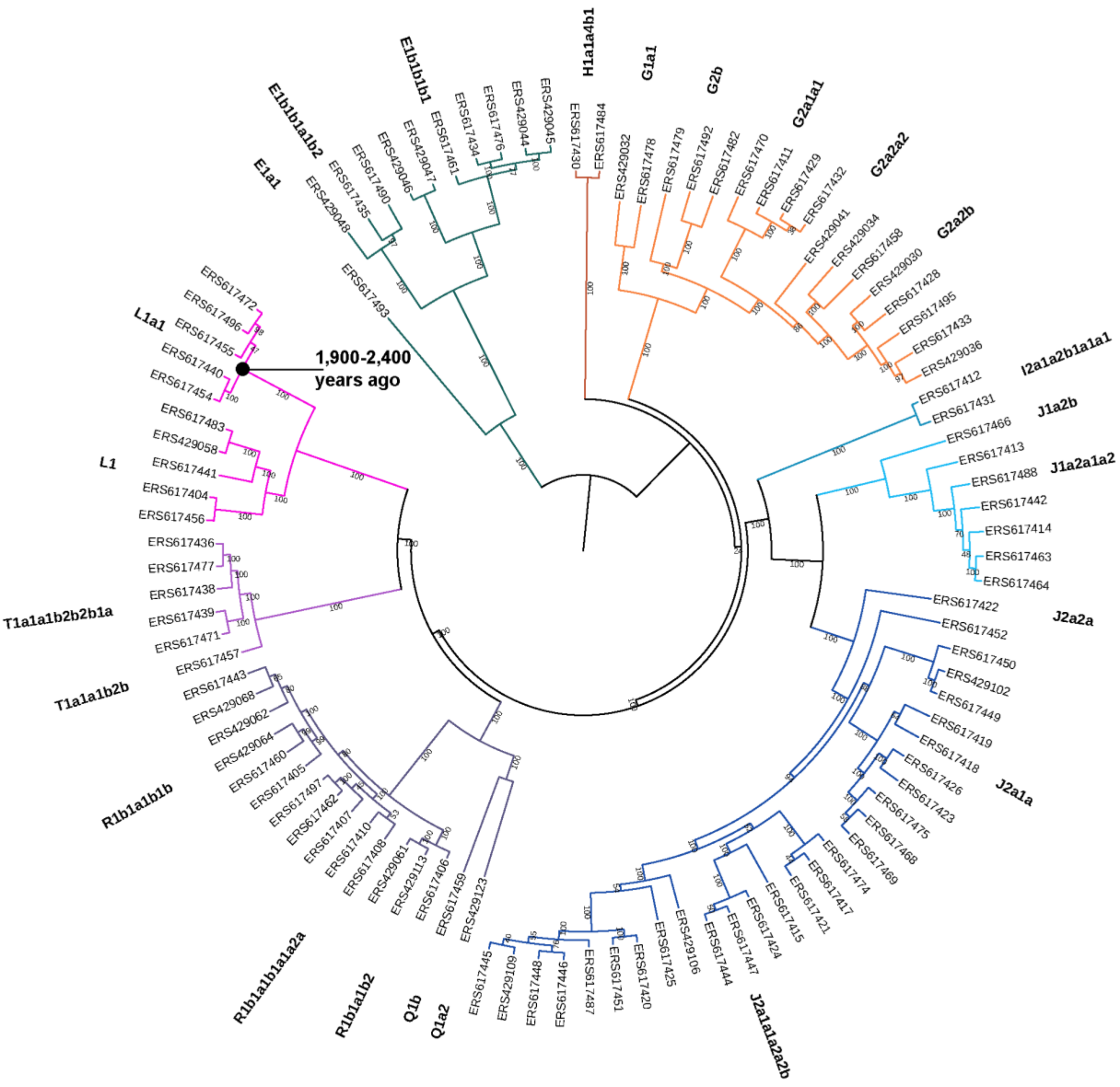

Figure S10. Y Chromosome phylogenetic tree from Lebanese males. A maximum-likelihood tree of 99 Y chromosome sequences from Lebanon. Different branch colors represent different haplogroups annotated according to the International Society of Genetic Genealogy (ISOGG V 14.255). We found that the Central/South Asian Y Chromosomes L1a1 present in Lebanon coalesced 1,900-2,400 years ago during the Hellenistic period when admixture with a Central/South Asian population can also be detected from genome-wide inferences.

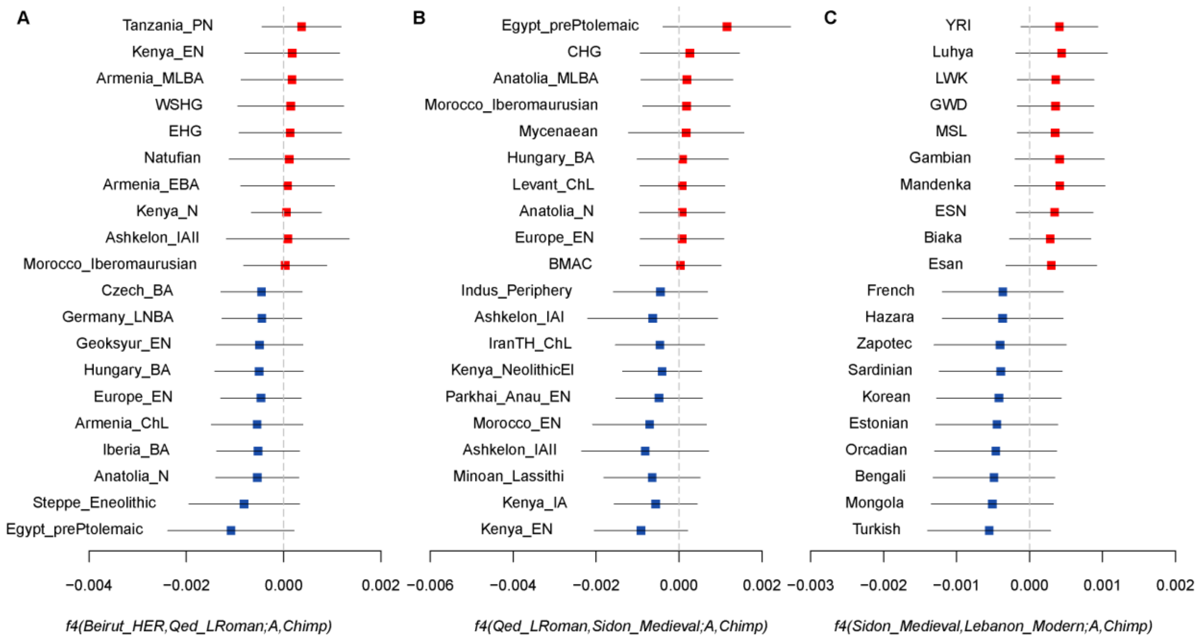

Figure S11. Genetic changes in Lebanon between the Hellenistic/Early Roman periods and the present-day. (A) Comparing the Hellenistic/Early Roman period population with the Late Roman period population, (B) the Late Roman period with the medieval period, and (C) changes between the medieval period and the present-day.

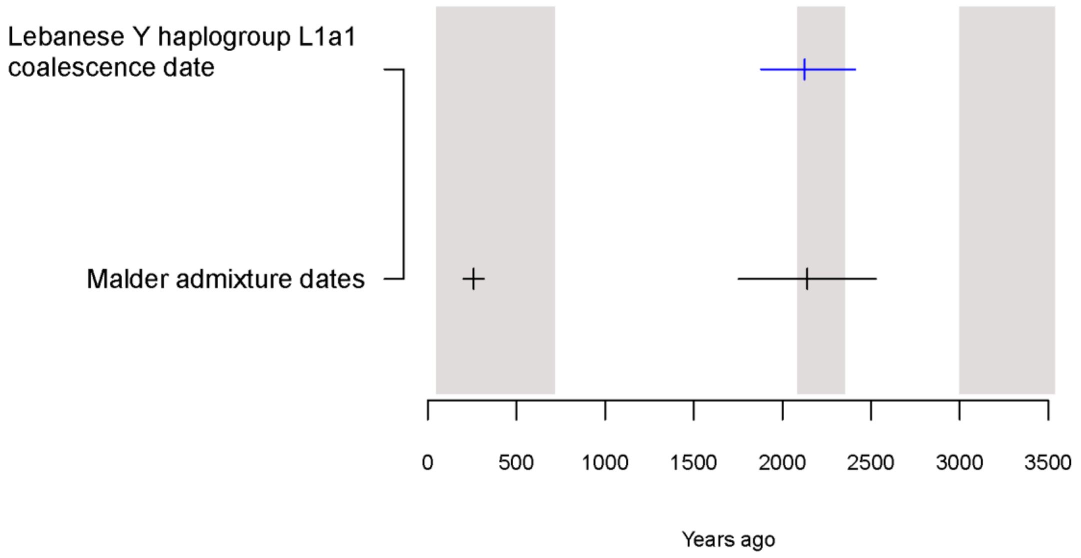

Figure S12. Admixture signals using modern and ancient Lebanese. Grey shades indicate the time period when admixture is directly detected (Table 2 and S11) from a sampled population. Blue cross shows coalescence time of the L1a1 Y chromosomes in Lebanon. Black crosses show admixture time estimated from decay of linkage disequilibrium (LD).

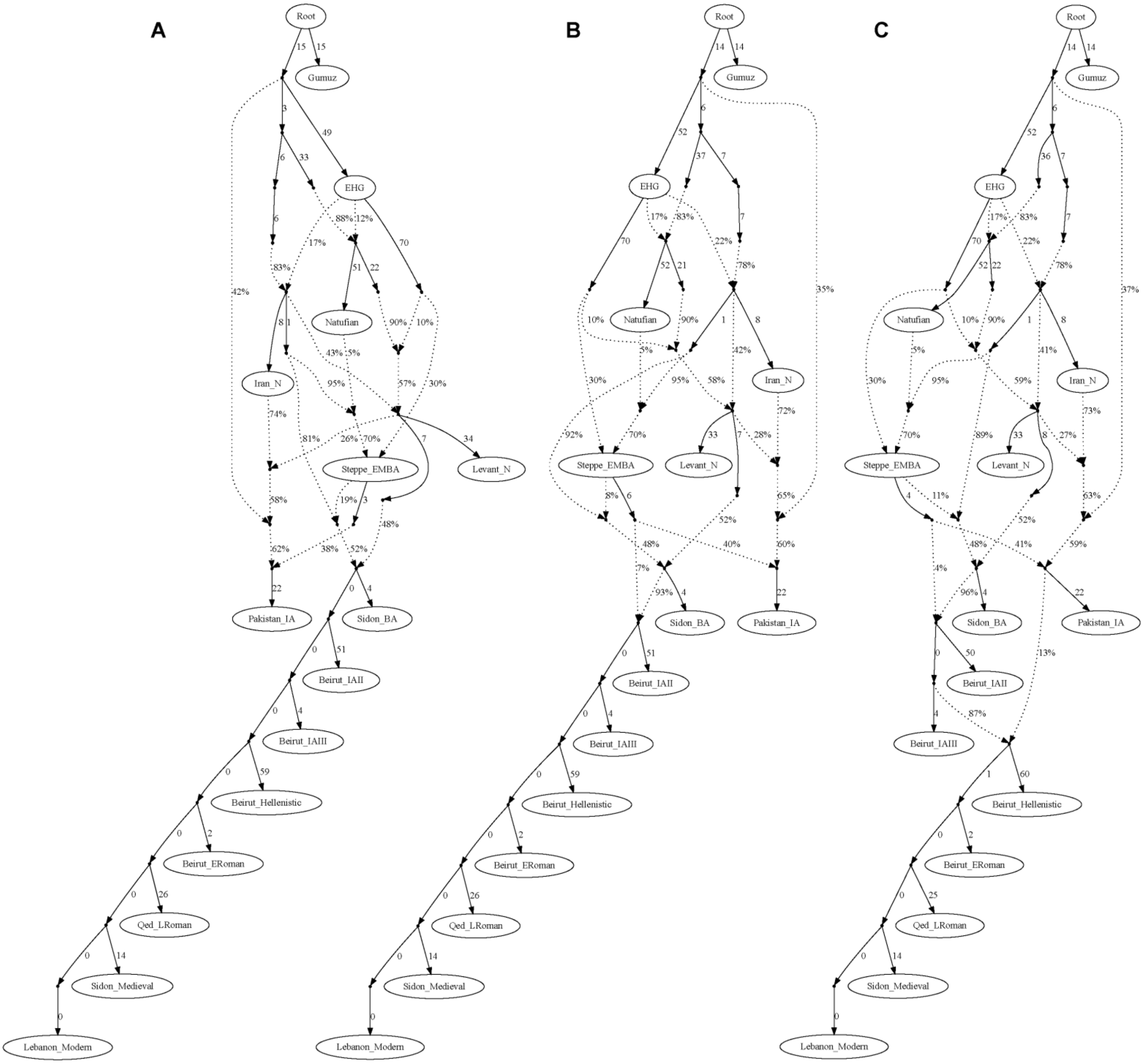

Figure S13. qpGraph models showing possible population relationships in ancient Lebanon. The admixture graph model with three limited admixtures in ancient Lebanon (main text Figure 3) has a worst Z-score=3.0 for the difference between estimated and fitted f-statistics. (A) A model with full genetic continuity in Lebanon after the Bronze Age has worst Z-score=9.5. (B) Adding an admixture event during the Iron Age II reduces the Z-score to 8.2. (C) Adding a second admixture event during the Hellenistic period reduces the Z-score to 4.0.

Supplemental Tables

ENA number	ID	Analyzis ID	Excavation Site	Burial ID	Period	Date (cal)	Civilization	Study
ERS1790729	SI-23	Sidon_BA	Sidon	46	Middle Bronze Age	-	Canaanite	Haber et al. 2017[5]
ERS1790730	SI-09	Sidon_BA	Sidon	65	Middle Bronze Age	-	Canaanite	Haber et al. 2017
ERS1790731	SI-21	Sidon_BA	Sidon	75	Middle Bronze Age	-	Canaanite	Haber et al. 2017
ERS1790732	SI-12	Sidon_BA	Sidon	63	Middle Bronze Age	-	Canaanite	Haber et al. 2017
ERS1790733	SI-22	Sidon_BA	Sidon	54	Middle Bronze Age	1950-1730BCE	Canaanite	Haber et al. 2017
ERS4542976	SFI-56	Beirut_IAII	Beirut SFI-415	T43 cxt 7854	Iron Age II	-	Assyrian	This study
ERS4542991	SFI-55	Beirut_IAII	Beirut SFI-415	T43 cxt 7855	Iron Age II	-	Assyrian	This study
ERS4542962	SFI-43	Beirut_IAIII	Beirut SFI-1075	T24 cxt 1592	Iron Age III	-	Achaemenid	This study
ERS4542967	SFI-50	Beirut_IAIII	Beirut SFI-1075	T38 cxt 5534	Iron Age III	-	Achaemenid	This study
ERS4542969	SFI-36	Beirut_IAIII	Beirut SFI-1075	T8	Iron Age III	-	Achaemenid	This study
ERS4542989	SFI-42	Beirut_IAIII	Beirut SFI-1075	T21	Iron Age III	540BCE-396BCE	Achaemenid	This study
ERS4542964	SFI-45	Beirut_IAIII	Beirut SFI-1075	T25 cxt 5249	Iron Age III	-	Achaemenid	This study
ERS4542984	SFI-34	Beirut_IAIII	Beirut SFI-1075	T5 cxt 1062	Iron Age III	-	Achaemenid	This study
ERS4542983	SFI-35	Beirut_IAIII	Beirut SFI-1075	T6 cxt 1055	Iron Age III	-	Achaemenid	This study
ERS4542988	SFI-39	Beirut_IAIII	Beirut SFI-1075	T12	Iron Age III	-	Achaemenid	This study
ERS4542990	SFI-44	Beirut_IAIII	Beirut SFI-1075	cxt 1593	Iron Age III	-	Achaemenid	This study
ERS4542987	SFI-47	Beirut_IAIII	Beirut SFI-1075	T27 cxt 2542	Iron Age III	-	Achaemenid	This study
ERS4542979	SFI-20	Beirut_Hellenistic	Beirut SFI-477	T42	Hellenistic	199BCE-37BCE	Hellenistic	This study
ERS4542972	SFI-5	Beirut_Hellenistic	Beirut SFI-477	T9	Hellenistic	234BCE-92BCE	Hellenistic	This study
ERS4542974	SFI-12	Beirut_Hellenistic	Beirut SFI-477	T27	Hellenistic	209BCE-89BCE	Hellenistic	This study
ERS4542980	SFI-24	Beirut_ERoman	Beirut SFI-1106	T8	Early Roman	55BCE-58CE	Roman	This study
ERS4542982	SFI-33	Beirut_ERoman	Beirut SFI-1106	T18	Early Roman	48CE-222CE	Roman	This study
ERS4542973	SFI-11	Beirut_ERoman	Beirut SFI-477	T24	Early Roman	119BCE-27CE	Roman	This study
ERS4542977	SFI-15	Beirut_ERoman	Beirut SFI-477	T32	Early Roman	176BCE-3CE	Roman	This study
ERS3189333	QED-2	Qed_LRoman	Qornet ed-Deir	QED	Late Roman	244-400CE	Roman	Haber et al.2019[6]
ERS3189335	QED-4	Qed_LRoman	Qornet ed-Deir	QED	Late Roman	426-632CE	Roman	Haber et al.2019
ERS3189338	QED-7	Qed_LRoman	Qornet ed-Deir	QED	Late Roman	237-389CE	Roman	Haber et al.2019
ERS3189342	QED-12	Qed_LRoman	Qornet ed-Deir	QED	Late Roman	-	Roman	Haber et al.2019
ERS3189349	SI-38	Sidon_Medieval	Sidon	110	Medieval	-	Crusaders	Haber et al.2019
ERS3189353	SI-42	Sidon_Medieval	Sidon	110	Medieval	1154-1281CE	Crusaders	Haber et al.2019
ERS3189348	SI-44	Sidon_Medieval	Sidon	101	Medieval	-	Crusaders	Haber et al.2019
ERS3189355	SI-45	Sidon_Medieval	Sidon	110	Medieval	1219-1278CE	Crusaders	Haber et al.2019

Table S1. Ancient samples from Lebanon analyzed in this study

ID	Contamination % estimated from Male X
SFI-55	2.32±1.1
SFI-42	1.6±1.1
SFI-45	0.61±0.8
SFI-34	0.86±0.3
SFI-35	1.56±1.3
SFI-39	1.09±0.7
SFI-44	1.32±0.4
SFI-47	1.37±0.4
SFI-5	2.78±3.9
SFI-12	3.81±3.8
SFI-11	2.37±3
SFI-15	0.93±0.3

Table S2. Contamination estimates from males' X chromosome. Using ANGSD[7] with options -b 2700000 -c 154900000 -d 2 -m 0 -f 1. Showing results from Method1 new llh version.[8]

ID	Contamination estimate (low-high) from mtDNA
SFI-56	0.01 (0-0.02)
SFI-55	0.01 (0-0.02)
SFI-43	0.01 (0-0.02)
SFI-50	0.01 (0-0.02)
SFI-36	0.01 (0-0.02)
SFI-42	0 (0-0.01)
SFI-45	0 (0-0.01)
SFI-34	0.01 (0-0.02)
SFI-35	0.01 (0-0.02)
SFI-39	0.01 (0-0.02)
SFI-44	0 (0-0.01)
SFI-47	0.01 (0-0.02)
SFI-20	0.01 (0-0.02)
SFI-5	0.01 (0-0.02)
SFI-12	0.01 (0-0.02)
SFI-24	0.01 (0-0.02)
SFI-33	0.01 (0-0.02)
SFI-11	0.01 (0-0.02)
SFI-15	0.01 (0-0.02)

Table S3. Contamination estimates from mtDNA. Using schmutzi[9] with the --uselength option.

ID	Period	Sex (genetic)	Y Haplogroup	MT Haplogroup
SFI-56	Iron Age II	female	-	U1a1a
SFI-55	Iron Age II	male	J	H2a
SFI-43	Iron Age III	female	-	T2c1+146
SFI-50	Iron Age III	female	-	U1a
SFI-36	Iron Age III	female	-	R0a1a
SFI-42	Iron Age III	male	J1-M267	H2a
SFI-45	Iron Age III	male	J-M304	T2a1b1
SFI-34	Iron Age III	male	J1-M267	T1a2
SFI-35	Iron Age III	male	I2a1b-M436	R0a1a
SFI-39	Iron Age III	male	H2-P96	I1b
SFI-44	Iron Age III	male	J1a2a1a2-P58	T2c1+146
SFI-47	Iron Age III	male	G2a2a1a2-L91	W6
SFI-20	Hellenistic	female	-	H41
SFI-5	Hellenistic	male	Q1b-M346	K1a5a
SFI-12	Hellenistic	male	E1b1b1a1a2-V65	H14a
SFI-24	Early Roman	female	-	H8b
SFI-33	Early Roman	female	-	T1
SFI-11	Early Roman	male	G2a2b-L30	N1b1
SFI-15	Early Roman	male	G2a2b1a2-M3302	I1c1

Table S4. Ancient samples' sex and uniparental haplogroups.

A	B	P value for rank=0
SFI-43	Egypt_prePtolemaic	7.63E-02
SFI-43	SFI-44	1.24E-02
SFI-43	Egypt_Ptolemaic	1.14E-03
SFI-43	Ashkelon_IAII	1.03E-09
SFI-43	Beirut_Hellenistic	5.71E-10
SFI-44	SFI-43	1.24E-02
SFI-44	Egypt_Ptolemaic	2.12E-03
SFI-44	Ashkelon_IAII	1.84E-03
SFI-44	Egypt_prePtolemaic	2.09E-04
SFI-44	Beirut_Hellenistic	1.73E-04

Table S5. Test for affinity of individuals SFI-43 and SFI-44 to a population *B* in the dataset. We show the top 5 results for each individual based on the P value for rank=0 and highlight in red the instances where the P value is >0.05 indicating *A* forms a clade with *B*.

				Mixture proportions		
Test	A	B	P value for rank=1	A	B	Std. Error
SFI-44	SFI-43	Beirut_IAIII	6.87E-01	0.73	0.27	0.07
SFI-44	SFI-43	Sidon_BA	6.48E-01	0.71	0.29	0.08
SFI-44	SFI-43	Ashkelon_IAII	6.23E-01	0.60	0.39	0.10
SFI-44	SFI-43	Qed_LRoman	5.67E-01	0.73	0.27	0.07
SFI-44	SFI-43	Beirut_IAII	4.99E-01	0.68	0.32	0.09
SFI-44	SFI-43	*hybrid*	5.98E-01	0.49	0.51	0.1

Table S6. Modelling SFI-44 as a mixture of SFI-43 and a population *B*. We report the top 5 models and the model involving a *hybrid* genome constructed by mixing an ancient Egyptian (JK2888) and an individual (SFI-34) from the Beirut_IAIII population. A P value > 0.05 (red) indicates the model cannot be rejected.

Test	A	B	P value for rank=1	Mixture proportions A	Mixture proportions B	Std. Error
Beirut_IA	Sidon_BA	Sardinian	1.23E-02	0.91	0.09	0.02
Beirut_IA	Sidon_BA	Tuscan	3.83E-03	0.90	0.10	0.03
Beirut_IA	Sidon_BA	Basque	3.18E-03	0.93	0.07	0.02
Beirut_IA	Sidon_BA	Cretan	1.87E-03	0.87	0.14	0.04
Beirut_IA	Sidon_BA	French	1.85E-03	0.94	0.06	0.02

Table S7. Iron Age admixture proportions when *B* is a modern population. In this table and following tables we show the top 5 models based on the P value for rank=1 and highlight in red the instances where the P value is >0.05 indicating the model cannot be rejected.

Test	A	B	P value for rank=1	Mixture proportions A	Mixture proportions B	Std. Error
Beirut_HER	Beirut_IA	Makrani	7.61E-01	0.89	0.11	0.02
Beirut_HER	Beirut_IA	Brahui	7.59E-01	0.90	0.10	0.02
Beirut_HER	Beirut_IA	Iranian	7.15E-01	0.84	0.17	0.03
Beirut_HER	Beirut_IA	Balochi	7.02E-01	0.91	0.10	0.02
Beirut_HER	Beirut_IA	Kalash	6.04E-01	0.92	0.08	0.02

Table S8. Hellenistic/Early Roman period admixture proportions when *B* is a modern population.

Test	A	B	P value for rank=1	Mixture proportions A	Mixture proportions B	Mixture proportions Std. Error
Qed_LRoman	Beirut_HER	Anatolia_EBA	9.89E-02	0.86	0.14	0.06
Qed_LRoman	Beirut_HER	Greece_Minoan_Lassithi	9.42E-02	0.91	0.10	0.04
Qed_LRoman	Beirut_HER	Anatolia_MLBA	8.54E-02	0.74	0.26	0.12
Qed_LRoman	Beirut_HER	Greece_Mycenaean	7.53E-02	0.85	0.15	0.06
Qed_LRoman	Beirut_HER	Greece_N	6.92E-02	0.93	0.07	0.03
Qed_LRoman	Beirut_Iron	Anatolia_EBA	1.05E-02	1.01	-0.01	0.07
Qed_LRoman	Beirut_Iron	Greece_Minoan_Lassithi	2.18E-02	1.07	-0.07	0.04
Qed_LRoman	Beirut_Iron	Anatolia_MLBA.SG	1.23E-02	1.08	-0.08	0.21
Qed_LRoman	Beirut_Iron	Greece_Mycenaean	3.09E-02	1.07	-0.07	0.03
Qed_LRoman	Beirut_Iron	Greece_N	1.29E-02	1.07	-0.07	0.09

Table S9. Modelling the Late Roman period population as a mixture of the local populations preceding in time and an ancient population *B*.

Test	A	B	P value for rank=1	Mixture proportions A	Mixture proportions B	Mixture proportions Std. Error
Sidon_Medieval	Qed_LRoman	Kenya_N	3.47E-02	0.98	0.03	0.01
Sidon_Medieval	Qed_LRoman	Kenya_NeolithicEl	3.40E-02	0.98	0.02	0.01
Sidon_Medieval	Qed_LRoman	Kenya_EN	3.38E-02	0.97	0.03	0.01
Sidon_Medieval	Qed_LRoman	Tanzania_PN	3.09E-02	0.98	0.02	0.01
Sidon_Medieval	Qed_LRoman	Kenya_IA	2.41E-02	0.98	0.02	0.01

Table S10. Modelling the Lebanon medieval population as a mixture of the local Late Roman period population and an ancient population *B*.

Test	A	B	P value for rank=1	Mixture proportions A	Mixture proportions B	Mixture proportions Std. Error
Lebanon_Modern	Sidon_Medieval	Abkhasian	1.81E-01	0.90	0.10	0.03
Lebanon_Modern	Sidon_Medieval	Armenian	1.68E-01	0.83	0.17	0.06
Lebanon_Modern	Sidon_Medieval	North Ossetian	1.12E-01	0.93	0.07	0.03
Lebanon_Modern	Sidon_Medieval	Adygei	9.29E-02	0.93	0.07	0.03
Lebanon_Modern	Sidon_Medieval	Turkish	7.43E-02	0.91	0.09	0.04

Table S11. Modelling the Lebanon modern population as a mixture of the local medieval period population and a modern population *B*.

Supplemental Methods

Sequences processing and genotyping

We processed the new sequences using the PALEOMIX[10] pipeline retaining reads ≥30 bp and collapsing pairs with minimum overlap of 15 bp while allowing a mismatch rate of 1/3 between the pairs. We mapped the merged sequences with BWA-backtrack v0.7.15[11] to the hs37d5 reference sequence, removed duplicates, and removed bases from the end of the reads until the frequency of nucleotide misincorporation estimated with mapDamage v2.0.6-2-g6507525[3] dropped to below 5%. We used ANGSD v0.925-21-g5de79b5[7] to randomly sample a single sequence with a minimum base quality of ≥20 to represent each SNP.

Datasets

We created two datasets for the analysis by merging the new data with published data. *Set1* included ancient individuals (Table S12) extracted from a previously merged dataset of genomes available from the Reich lab https://reich.hms.harvard.edu/downloadable-genotypes-present-day-and-ancient-dna-data-compiled-published-papers (v42.4). We additionally extracted from this dataset modern genomes from South Asia,[12] from worldwide populations described in the 1000 Genomes Project,[13] and from the Simons Genome Diversity Project (SGDP).[14] We added individuals from ancient and modern Lebanon[5; 6] and modern Egypt and Ethiopia.[15] From Medieval Lebanon, we only analysed individuals who represented the local ancestry:[6] SI-38, SI-42, SI-44, and SI-45 and removed the outlier SI-44 when samples where pooled into the Sidon_Medieval group.

We merged the datasets using the mergeit program available from the EIGENSOFT package v7.2.1[16] with options docheck: YES and strandcheck: YES. We filtered out sex-linked and triallelilic SNPs and sites that were outside the 1000 Genomes Project's strict mask resulting in a dataset of 2012 modern humans and 914 ancient individuals with 815,791 SNPs. In addition, we created *Set2* which consisted of modern individuals from worldwide populations genotyped on the Human Origins array[17-19] merged with the ancient individuals to obtain a dataset of 2788 modern humans and 914 ancient individuals with 539,766 SNPs.

Ancient samples' sex and uniparental haplogroups

We determined the sex of the samples from the ratio of sequences aligning to the X and Y chromosomes.[20] We genotyped the Y chromosome of the ancient males jointly with the 1000 Genomes Project[13] and modern Lebanese[5] males using freebayes v1.3.1[21] with options --report-monomorphic --ploidy 1 --min-base-quality 20 --min-mapping-quality 30 and restricted the calling to 10.3 Mb of the Y chromosome previously determined to be accessible to short-read sequencing.[22] We

determined the Y haplogroup using yHaplo[23] and according to ISOGG v14.255 annotations. We determined the mtDNA haplogroup by uploading the BAM files the mtDNA server.[24]

Y Chromosome phylogeny and dating

We extracted Y chromosome genotypes of modern Lebanese from the jointly called dataset described in the previous section. We then inferred a maximum likelihood phylogeny using RAxML v8.2.10[25] with arguments -m ASC_GTRGAMMA and --asc-corr=stamatakis, using variable sites with QUAL ≥1. We selected the tree with the best likelihood from 100 runs and replicated it 1000 times for bootstrap values. We determined the nodes' ages using the ρ statistic[26] and defined the ancestral state of a site according to our previous study[27] by assigning alleles as ancestral when they were monomorphic in the nine samples belonging to the A and B haplogroups in the SGDP dataset. We then determined the age of the L1a1 node as follows: The ancestral node comprised two clades, we select one sample from each clade (for example ERS617454 and ERS617455) and divide the number of derived variants found in the first sample but absent from the second by the total number of sites having the ancestral state in both samples. We repeat for all possible pairs under the L1a1 node and report the average value of divergence times in units of years by applying a point mutation rate of 0.76×10^{-9} ($0.67\text{-}0.86 \times 10^{-9}$ 95%CI) mutations per site per year.[28]

Principal Component analysis and DyStruct

We used smartpca v16000 from the EIGENSOFT package[16] to compute a PCA using parameters numoutlieriter: 0, lsqproject: YES, autoshrink: YES and using only variation in modern populations selected to represent genetic diversity in Central Asia, the Near East, and Europe.

We run DyStruct with default arguments and using 166,693 transversions found in *Set1* across nine time points binned as follows (in years ago): 14,500-10,000; 10,000-8000; 8000-6000; 6000-5200; 5200-5000; 5000-3000; 3000-1400;1400-200; and present-day.

***f4* statistics**

We used *qpDstat* v755 from the ADMIXTOOLS package[17] with parameter f4mode: YES to test genetic continuity in Lebanon using significant (±3 standard errors) deviation from zero in the statistic *f4*(Period1,Period2,A,Chimpanzee) as indicating a possible genetic change between two subsequent periods related to A (any ancient population in our Set1 dataset). We exclude from the test populations with single individuals or with overlapping number of SNPs <200,000. When testing genetic change in Lebanon_Modern we choose A to be any modern population in our Set1 dataset.

qpWave/qpAdm analysis

SFI-43 and SFI-44

We used *qpWave* v410 and *qpAdm* v810[29; 30] from the ADMIXTOOLS package[17] with option allsnps:YES to determine if SFI-43 and SFI-44 formed a clade with any ancient population in *Set1* and if SFI-44 can be modelled as a mixture of ancestries related to SFI-43 and any other ancient individual or population in our dataset. We selected 11 outgroups that are related differently to the ancient population in our dataset: Ust'-Ishim (a 45,000-year-old Siberian), Eastern hunter-gatherers from Russia (EHG), Sweden hunter-gatherers (SHG), Caucasus hunter-gatherers (CHG), Morocco Iberomaurusian, Levant Natufian, Levant Chalcolithic, Anatolia Neolithic, Iran Neolithic, in addition to modern populations Han and Mbuti.

Admixture in ancient Lebanon

We used *qpAdm* to model ancient Lebanon populations as a mixture of two streams of ancestries, the first source related to the local population which preceded the tested population in time and the second source deriving from a population found in our *Set1* dataset. We used 19 outgroups:
Ust'-Ishim, Mota (a 4,500-year-old from Ethiopia), EHG, SHG, CHG, Western hunter-gatherers from Europe (WHG), West Siberian hunter-gatherers (WSHG), Morocco Early Neolithic, Levant Natufian, Levant Chalcolithic, Anatolia Neolithic, Europe Early Neolithic, Germany Late Neolithic/Bronze Age, Iberia Middle Neolithic/Chalcolithic, Iran Neolithic, Iran Chalcolithic (Tepe Hissar), Turkmenistan Neolithic (Geoksyur), Mbuti and Han. We also rotate these outgroups and test them as a second source of ancestry while keeping the remaining 18 populations in the outgroup set.

Genotype imputation and ChromoPainter analysis

We used ChromoPainter[31] and followed the method described by Antonio et al.[32] who showed that common variants (>1% MAF in the 1000 Genomes Project) in low-coverage genomes (as low as 0.1x) can be imputed with relatively high accuracy in ancient European and Near Eastern samples using the 1000 Genomes Project reference panels. We used GATK UnifiedGenotyper[33] to estimate genotypes likelihoods from the ancient samples for common variants (>1% minor allele frequency) found in the 1000 Genomes Project reference panel[13] using parameters: min_base_quality_score 30 --output_mode EMIT_ALL_SITES --allSitePLs –alleles <reference_panel> --genotyping_mode GENOYTPE_GIVEN_ALLELES -R <hg19 reference fasta>. We then used Beagle v4.0[34] for imputation with parameters: gprobs=true, impute=true, gl=<UnifiedGenotyper output>, ref=<Beagle imputation reference panel>, map <GRCh37 recombination map>.
The Reference panel and recombination maps were downloaded from the Beagle website (https://faculty.washington.edu/browning/beagle/b4_1.html). We extracted a subset of 196 modern

human samples relevant to our study from the Human Origins dataset[17-19] and imputed them with the Michigan server (https://imputationserver.sph.umich.edu/index.html) using the 1000 Genomes Project phase 3 reference panel.[13] We then ran the ChromoPainter/finestructure v4.0.1[31] inference pipeline with default settings outputting a co-ancestry matrix with values depicting copies of haplotype segments shared between individuals.

MALDER

We used MALDER v1.0[35; 36] with parameters mindis: 0.005, binsize: 0.0005 and a generation time of 30 years to estimate admixture time in the modern population from decay of linkage disequilibrium (LD). We used the modern populations in *Set2* as proxies for the admixing ancestry sources.

qpGraph

We used *qpGraph* v6450[17] to draw a phylogenetic model that tests the models obtained from the *qpAdm* results. We started by a basic graph similar to the one we used to explain genetic relationships in the Near East[37] but using populations: Gumuz, Iran_N, EHG, Natufian, Levant_N, Pakistan_IA (Aligrama2_IA), Steppe_EMBA, Sidon_BA and Lebanon_Modern. This graph captures some of the previous knowledge on these population relationships, for example the Sidon_BA population deriving ~50% of its ancestry from the local population and ~50% from a population related to Iran_N.[5] However, we warn that our admixture graph is not intended to model the deep relationships between the ancient reference populations used here and is not a full representation of their complex admixture history. We used the *qpGraph* models to highlight a substantial genetic continuity in Lebanon interrupted by few admixture episodes and showed that it fits the models obtained from *qpAdm*. We added to the above basic set of populations Beirut_IAII, Beirut_IAIII, Beirut_Hellenistic, Beirut_ERoman, Qed_LRoman, and Sidon_Medieval. We found that the graph showing complete genetic continuity in Lebanon and without admixture after the Bronze Age has several outlier f-statistic results from unaccounted-for relationships between the Lebanese populations and other ancient populations (Z-score=9.5) (Figure S13A). We added the first admixture event observed in the *qpAdm* results during the Iron Age II from a Steppe-related population, this slightly improved the Z-scores (Figure S13B). We then added a Central Asian related admixture to the Hellenistic population and further improved the Z-scores (Figure S13C) but remained with a worst Z-score=4 from an unaccounted-for relationship between the Steppe and Lebanon_Modern. We finally added an admixture edge to Lebanon_Modern and obtained a worst f-statistic Z-score=3.0 (main text Figure 3).

Supplemental References

1. Elayi, J., and Sayegh, H. (1998). Un quartier du port phénicien de Beyrouth au Fer III - Perse.(Paris: Gabalda).
2. Marriner, N., Morhange, C., and Saghieh-Beydoun, M. (2008). Geoarchaeology of Beirut's ancient harbour, Phoenicia. Journal of Archaeological Science 35, 2495-2516.
3. Jonsson, H., Ginolhac, A., Schubert, M., Johnson, P.L., and Orlando, L. (2013). mapDamage2.0: fast approximate Bayesian estimates of ancient DNA damage parameters. Bioinformatics 29, 1682-1684.
4. Monroy Kuhn, J.M., Jakobsson, M., and Gunther, T. (2018). Estimating genetic kin relationships in prehistoric populations. PLoS One 13, e0195491.
5. Haber, M., Doumet-Serhal, C., Scheib, C., Xue, Y., Danecek, P., Mezzavilla, M., Youhanna, S., Martiniano, R., Prado-Martinez, J., Szpak, M., et al. (2017). Continuity and Admixture in the Last Five Millennia of Levantine History from Ancient Canaanite and Present-Day Lebanese Genome Sequences. Am J Hum Genet 101, 274-282.
6. Haber, M., Doumet-Serhal, C., Scheib, C.L., Xue, Y., Mikulski, R., Martiniano, R., Fischer-Genz, B., Schutkowski, H., Kivisild, T., and Tyler-Smith, C. (2019). A Transient Pulse of Genetic Admixture from the Crusaders in the Near East Identified from Ancient Genome Sequences. Am J Hum Genet 104, 977-984.
7. Korneliussen, T.S., Albrechtsen, A., and Nielsen, R. (2014). ANGSD: Analysis of Next Generation Sequencing Data. BMC Bioinformatics 15, 356.
8. Rasmussen, M., Guo, X., Wang, Y., Lohmueller, K.E., Rasmussen, S., Albrechtsen, A., Skotte, L., Lindgreen, S., Metspalu, M., Jombart, T., et al. (2011). An Aboriginal Australian genome reveals separate human dispersals into Asia. Science 334, 94-98.
9. Renaud, G., Slon, V., Duggan, A.T., and Kelso, J. (2015). Schmutzi: estimation of contamination and endogenous mitochondrial consensus calling for ancient DNA. Genome Biol 16, 224.
10. Schubert, M., Ermini, L., Der Sarkissian, C., Jonsson, H., Ginolhac, A., Schaefer, R., Martin, M.D., Fernandez, R., Kircher, M., McCue, M., et al. (2014). Characterization of ancient and modern genomes by SNP detection and phylogenomic and metagenomic analysis using PALEOMIX. Nat Protoc 9, 1056-1082.
11. Li, H., and Durbin, R. (2009). Fast and accurate short read alignment with Burrows-Wheeler transform. Bioinformatics 25, 1754-1760.
12. Mondal, M., Casals, F., Xu, T., Dall'Olio, G.M., Pybus, M., Netea, M.G., Comas, D., Laayouni, H., Li, Q., Majumder, P.P., et al. (2016). Genomic analysis of Andamanese provides insights into ancient human migration into Asia and adaptation. Nat Genet 48, 1066-1070.
13. The 1000 Genomes Project Consortium. (2015). A global reference for human genetic variation. Nature 526, 68-74.
14. Mallick, S., Li, H., Lipson, M., Mathieson, I., Gymrek, M., Racimo, F., Zhao, M., Chennagiri, N., Nordenfelt, S., Tandon, A., et al. (2016). The Simons Genome Diversity Project: 300 genomes from 142 diverse populations. Nature 538, 201-206.
15. Pagani, L., Schiffels, S., Gurdasani, D., Danecek, P., Scally, A., Chen, Y., Xue, Y., Haber, M., Ekong, R., Oljira, T., et al. (2015). Tracing the route of modern humans out of Africa by using 225 human genome sequences from Ethiopians and Egyptians. Am J Hum Genet 96, 986-991.
16. Patterson, N., Price, A.L., and Reich, D. (2006). Population structure and eigenanalysis. PLoS Genet 2, e190.
17. Patterson, N., Moorjani, P., Luo, Y., Mallick, S., Rohland, N., Zhan, Y., Genschoreck, T., Webster, T., and Reich, D. (2012). Ancient admixture in human history. Genetics 192, 1065-1093.
18. Lazaridis, I., Patterson, N., Mittnik, A., Renaud, G., Mallick, S., Kirsanow, K., Sudmant, P.H., Schraiber, J.G., Castellano, S., Lipson, M., et al. (2014). Ancient human genomes suggest three ancestral populations for present-day Europeans. Nature 513, 409-413.

19. Lazaridis, I., Nadel, D., Rollefson, G., Merrett, D.C., Rohland, N., Mallick, S., Fernandes, D., Novak, M., Gamarra, B., Sirak, K., et al. (2016). Genomic insights into the origin of farming in the ancient Near East. Nature 536, 419-424.
20. Skoglund, P., Stora, J., Gotherstrom, A., and Jakobsson, M. (2013). Accurate sex identification of ancient human remains using DNA shotgun sequencing. Journal of Archaeological Science 40, 4477-4482.
21. Garrison, E., and Marth, G. (2012). Haplotype-based variant detection from short-read sequencing. arXiv preprint arXiv:1207.3907 [q-bio.GN].
22. Poznik, G.D., Henn, B.M., Yee, M.C., Sliwerska, E., Euskirchen, G.M., Lin, A.A., Snyder, M., Quintana-Murci, L., Kidd, J.M., Underhill, P.A., et al. (2013). Sequencing Y chromosomes resolves discrepancy in time to common ancestor of males versus females. Science 341, 562-565.
23. Poznik, G.D. (2016). Identifying Y-chromosome haplogroups in arbitrarily large samples of sequenced or genotyped men. bioRxiv.
24. Weissensteiner, H., Forer, L., Fuchsberger, C., Schopf, B., Kloss-Brandstatter, A., Specht, G., Kronenberg, F., and Schonherr, S. (2016). mtDNA-Server: next-generation sequencing data analysis of human mitochondrial DNA in the cloud. Nucleic Acids Res 44, W64-69.
25. Stamatakis, A. (2014). RAxML version 8: a tool for phylogenetic analysis and post-analysis of large phylogenies. Bioinformatics 30, 1312-1313.
26. Forster, P., Harding, R., Torroni, A., and Bandelt, H.J. (1996). Origin and evolution of Native American mtDNA variation: a reappraisal. Am J Hum Genet 59, 935-945.
27. Haber, M., Jones, A.L., Connell, B.A., Asan, Arciero, E., Yang, H., Thomas, M.G., Xue, Y., and Tyler-Smith, C. (2019). A Rare Deep-Rooting D0 African Y-Chromosomal Haplogroup and Its Implications for the Expansion of Modern Humans out of Africa. Genetics.
28. Fu, Q., Li, H., Moorjani, P., Jay, F., Slepchenko, S.M., Bondarev, A.A., Johnson, P.L., Aximu-Petri, A., Prufer, K., de Filippo, C., et al. (2014). Genome sequence of a 45,000-year-old modern human from western Siberia. Nature 514, 445-449.
29. Haak, W., Lazaridis, I., Patterson, N., Rohland, N., Mallick, S., Llamas, B., Brandt, G., Nordenfelt, S., Harney, E., Stewardson, K., et al. (2015). Massive migration from the Steppe was a source for Indo-European languages in Europe. Nature 522, 207-211.
30. Reich, D., Patterson, N., Campbell, D., Tandon, A., Mazieres, S., Ray, N., Parra, M.V., Rojas, W., Duque, C., Mesa, N., et al. (2012). Reconstructing Native American population history. Nature 488, 370-374.
31. Lawson, D.J., Hellenthal, G., Myers, S., and Falush, D. (2012). Inference of population structure using dense haplotype data. PLoS Genet 8, e1002453.
32. Antonio, M.L., Gao, Z., Moots, H.M., Lucci, M., Candilio, F., Sawyer, S., Oberreiter, V., Calderon, D., Devitofranceschi, K., Aikens, R.C., et al. (2019). Ancient Rome: A genetic crossroads of Europe and the Mediterranean. Science 366, 708-714.
33. DePristo, M.A., Banks, E., Poplin, R., Garimella, K.V., Maguire, J.R., Hartl, C., Philippakis, A.A., del Angel, G., Rivas, M.A., Hanna, M., et al. (2011). A framework for variation discovery and genotyping using next-generation DNA sequencing data. Nat Genet 43, 491-498.
34. Browning, S.R., and Browning, B.L. (2007). Rapid and accurate haplotype phasing and missing-data inference for whole-genome association studies by use of localized haplotype clustering. Am J Hum Genet 81, 1084-1097.
35. Loh, P.R., Lipson, M., Patterson, N., Moorjani, P., Pickrell, J.K., Reich, D., and Berger, B. (2013). Inferring admixture histories of human populations using linkage disequilibrium. Genetics 193, 1233-1254.
36. Pickrell, J.K., Patterson, N., Loh, P.R., Lipson, M., Berger, B., Stoneking, M., Pakendorf, B., and Reich, D. (2014). Ancient west Eurasian ancestry in southern and eastern Africa. Proc Natl Acad Sci U S A 111, 2632-2637.

37. Haber, M., Saif-Ali, R., Al-Habori, M., Chen, Y., Platt, D.E., Tyler-Smith, C., and Xue, Y. (2019). Insight into the genomic history of the Near East from whole-genome sequences and genotypes of Yemenis. bioRxiv, 749341.
